# Supplementary material for: Nucleosome interaction of the CPC secures centromeric chromatin integrity and chromosome segregation fidelity
Source: EMBO J. 2025 Oct 27;44(22):6556–97. doi: 10.1038/s44318-025-00594-y (PMC12624148; doi:10.1038/s44318-025-00594-y)
Supplement: Supplementary file 2 — Movie EV1 [file 44318_2025_594_MOESM2_ESM.zip › Movie_EV1/Movie_EV1_Legend.docx]

**Movie_EV1**

**Legend**

**Swinging Motion of CPC Bound to H3T3ph Nucleosome.** The 3D variability analysis volume series was generated using the particles from Class 0, 1, and 2 represented in Fig. 2A (223,160 particles). The volume series was converted into a movie using Chimera 1.5.3. The map is displayed in grey against a black background. As the movie plays, the density corresponding to the CPC triple-helical bundle and Survivin BIR domain exhibits a swinging motion while remaining tethered to the NCP through the Borealin N-terminal tail-NCP acidic patch interaction. As the triple-helical bundle moves away from the DNA entry-exit site of the NCP, the DNA becomes more dynamic, adopting a partially open conformation.
