# Supplementary material for: Nucleosome interaction of the CPC secures centromeric chromatin integrity and chromosome segregation fidelity
Source: EMBO J. 2025 Oct 27;44(22):6556–97. doi: 10.1038/s44318-025-00594-y (PMC12624148; doi:10.1038/s44318-025-00594-y)
Supplement: Supplementary file 3 — Source data Fig. 1 [file 44318_2025_594_MOESM3_ESM.zip › Figure 1/Fig1B_C/D_1292150488_val-report-full_P1 (3).pdf]

# Full wwPDB EM Validation Report (i)

Aug 28, 2025 – 02:41 pm BST

| | | |
|---|---|---|
| PDB ID | : | 9SI3 / pdb_00009si3 |
| EMDB ID | : | EMD-54924 |
| Title | : | Chromosomal Passenger Complex in complex with H3T3ph Nucleosome |
| Deposited on | : | 2025-08-28 |
| Resolution | : | 2.83 Å(reported) |

**This wwPDB validation report is for manuscript review**

This is a Full wwPDB EM Validation Report.

This report is produced by the wwPDB biocuration pipeline after annotation of the structure.

We welcome your comments at *validation@mail.wwpdb.org*
A user guide is available at
https://www.wwpdb.org/validation/2017/EMValidationReportHelp
with specific help available everywhere you see the (i) symbol.

The types of validation reports are described at
http://www.wwpdb.org/validation/2017/FAQs#types.

For Manuscript Review

---

The following versions of software and data (see references (i)) were used in the production of this report:

| | | |
|---|---|---|
| EMDB validation analysis | : | 0.0.1.dev126 |
| MolProbity | : | 4-5-2 with Phenix2.0rc1 |
| Percentile statistics | : | 20231227.v01 (using entries in the PDB archive December 27th 2023) |
| MapQ | : | 1.9.13 |
| Ideal geometry (proteins) | : | Engh & Huber (2001) |
| Ideal geometry (DNA, RNA) | : | Parkinson et al. (1996) |
| Validation Pipeline (wwPDB-VP) | : | 2.45.1 |

# 1 Overall quality at a glance (i)

The following experimental techniques were used to determine the structure:
*ELECTRON MICROSCOPY*

The reported resolution of this entry is 2.83 Å.

Percentile scores (ranging between 0-100) for global validation metrics of the entry are shown in the following graphic. The table shows the number of entries on which the scores are based.

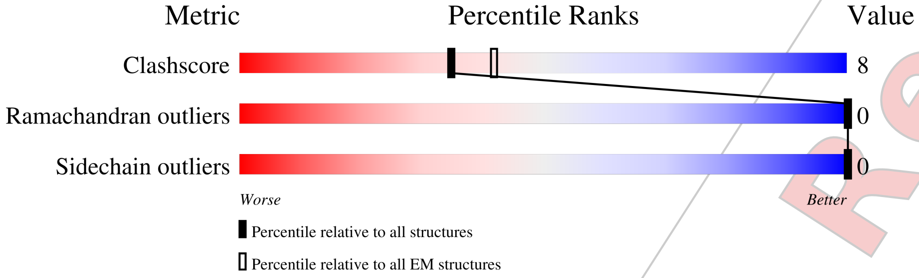


| Metric | Whole archive (#Entries) | EM structures (#Entries) |
|---|---|---|
| Clashscore | 210492 | 15764 |
| Ramachandran outliers | 207382 | 16835 |
| Sidechain outliers | 206894 | 16415 |

The table below summarises the geometric issues observed across the polymeric chains and their fit to the map. The red, orange, yellow and green segments of the bar indicate the fraction of residues that contain outliers for >=3, 2, 1 and 0 types of geometric quality criteria respectively. A grey segment represents the fraction of residues that are not modelled. The numeric value for each fraction is indicated below the corresponding segment, with a dot representing fractions <=5% The upper red bar (where present) indicates the fraction of residues that have poor fit to the EM map (all-atom inclusion < 40%). The numeric value is given above the bar.

| Mol | Chain | Length | Quality of chain |
|---|---|---|---|
| 1 | L | 280 | • • 95% |
| 1 | O | 280 | 22% • 73% |
| 2 | A | 135 | 62% 10% 27% |
| 2 | E | 135 | • / 65% 10% 25% |
| 3 | B | 102 | 65% 15% 21% |
| 3 | F | 102 | 70% 11% 20% |
| 4 | C | 129 | 76% 11% 13% |
| 4 | G | 129 | • / 83% 5% 12% |

*Continued on next page...*



For Manuscript Review

*Continued from previous page...*

| Mol | Chain | Length | Quality of chain |
|---|---|---|---|
| 5 | D | 125 | 65% 11% 24% |
| 5 | H | 125 | 69% 7% 24% |
| 6 | I | 147 | 58% 42% |
| 7 | J | 147 | 61% 39% |
| 8 | M | 142 | 13% 68% 28% • |
| 9 | N | 918 | • • 95% |

For Manuscript Review

# 2 Entry composition (i)

There are 9 unique types of molecules in this entry. The entry contains 14401 atoms, of which 0 are hydrogens and 0 are deuteriums.

In the tables below, the AltConf column contains the number of residues with at least one atom in alternate conformation and the Trace column contains the number of residues modelled with at most 2 atoms.

- Molecule 1 is a protein called Borealin.

| Mol | Chain | Residues | Atoms | AltConf | Trace |
|---|---|---|---|---|---|
| 1 | L | 14 | Total C N O<br>108 63 26 19 | 0 | 0 |
| 1 | O | 75 | Total C N O S<br>633 400 120 112 1 | 0 | 0 |

- Molecule 2 is a protein called Histone H3.2.

| Mol | Chain | Residues | Atoms | AltConf | Trace |
|---|---|---|---|---|---|
| 2 | A | 98 | Total C N O S<br>811 512 157 139 3 | 0 | 0 |
| 2 | E | 101 | Total C N O S<br>831 525 161 142 3 | 0 | 0 |

There are 2 discrepancies between the modelled and reference sequences:

| Chain | Residue | Modelled | Actual | Comment | Reference |
|---|---|---|---|---|---|
| A | 102 | ALA | GLY | conflict | UNP P84233 |
| E | 102 | ALA | GLY | conflict | UNP P84233 |

- Molecule 3 is a protein called Histone H4.

| Mol | Chain | Residues | Atoms | AltConf | Trace |
|---|---|---|---|---|---|
| 3 | B | 81 | Total C N O S<br>648 410 126 111 1 | 0 | 0 |
| 3 | F | 82 | Total C N O S<br>657 416 128 112 1 | 0 | 0 |

- Molecule 4 is a protein called Histone H2A type 1.

| Mol | Chain | Residues | Atoms | AltConf | Trace |
|---|---|---|---|---|---|
| 4 | C | 112 | Total C N O<br>862 545 169 148 | 0 | 0 |

*Continued on next page...*

For Manuscript Review

*Continued from previous page...*

| Mol | Chain | Residues | Atoms | | | | AltConf | Trace |
|---|---|---|---|---|---|---|---|---|
| 4 | G | 113 | Total 871 | C 550 | N 170 | O 151 | 0 | 0 |

- Molecule 5 is a protein called Histone H2B type 1-J.

| Mol | Chain | Residues | Atoms | | | | | AltConf | Trace |
|---|---|---|---|---|---|---|---|---|---|
| 5 | D | 95 | Total 746 | C 468 | N 136 | O 140 | S 2 | 0 | 0 |
| 5 | H | 95 | Total 746 | C 468 | N 136 | O 140 | S 2 | 0 | 0 |

- Molecule 6 is a DNA chain called DNA (147-MER).

| Mol | Chain | Residues | Atoms | | | | | AltConf | Trace |
|---|---|---|---|---|---|---|---|---|---|
| 6 | I | 147 | Total 3034 | C 1435 | N 572 | O 880 | P 147 | 0 | 0 |

- Molecule 7 is a DNA chain called DNA (147-MER).

| Mol | Chain | Residues | Atoms | | | | | AltConf | Trace |
|---|---|---|---|---|---|---|---|---|---|
| 7 | J | 147 | Total 2990 | C 1422 | N 540 | O 882 | P 146 | 0 | 0 |

- Molecule 8 is a protein called Baculoviral IAP repeat-containing protein 5.

| Mol | Chain | Residues | Atoms | | | | | AltConf | Trace |
|---|---|---|---|---|---|---|---|---|---|
| 8 | M | 137 | Total 1118 | C 712 | N 191 | O 207 | S 8 | 0 | 0 |

- Molecule 9 is a protein called Inner centromere protein.

| Mol | Chain | Residues | Atoms | | | | | AltConf | Trace |
|---|---|---|---|---|---|---|---|---|---|
| 9 | N | 42 | Total 346 | C 220 | N 54 | O 67 | S 5 | 0 | 0 |

For Manuscript Review

# 3 Residue-property plots (i)

These plots are drawn for all protein, RNA, DNA and oligosaccharide chains in the entry. The first graphic for a chain summarises the proportions of the various outlier classes displayed in the second graphic. The second graphic shows the sequence view annotated by issues in geometry and atom inclusion in map density. Residues are color-coded according to the number of geometric quality criteria for which they contain at least one outlier: green = 0, yellow = 1, orange = 2 and red = 3 or more. A red diamond above a residue indicates a poor fit to the EM map for this residue (all-atom inclusion < 40%). Stretches of 2 or more consecutive residues without any outlier are shown as a green connector. Residues present in the sample, but not in the model, are shown in grey.

- Molecule 1: Borealin

Chain L:

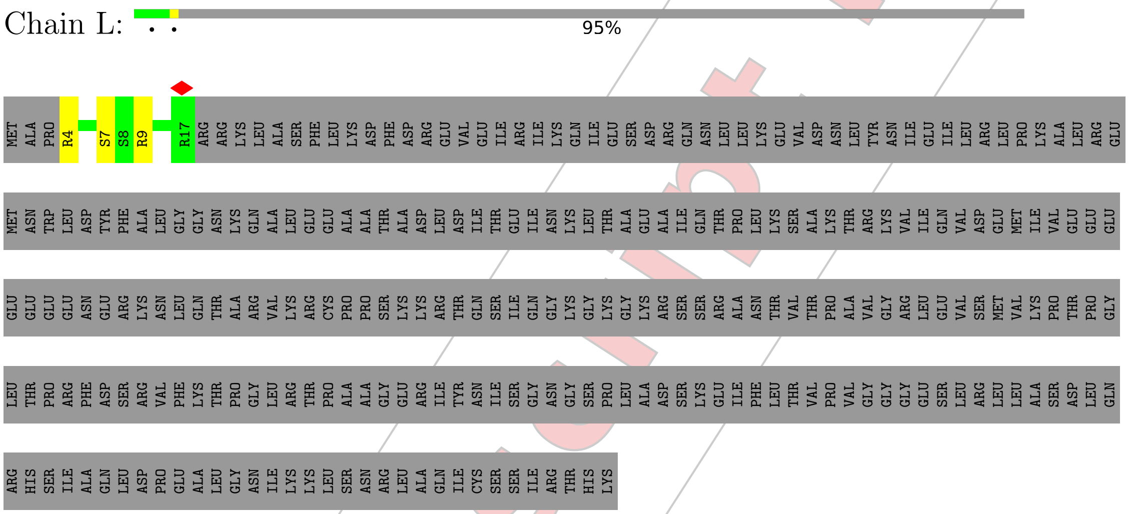


- Molecule 1: Borealin

Chain O:

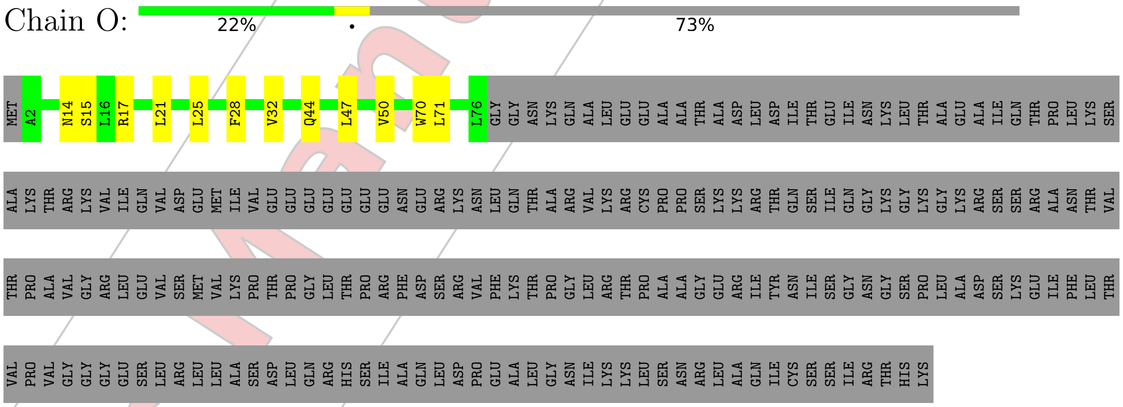


- Molecule 2: Histone H3.2

Chain A:

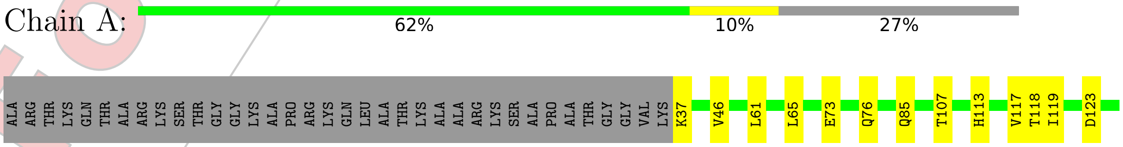


For Manuscript Review

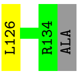


• Molecule 2: Histone H3.2

Chain E:

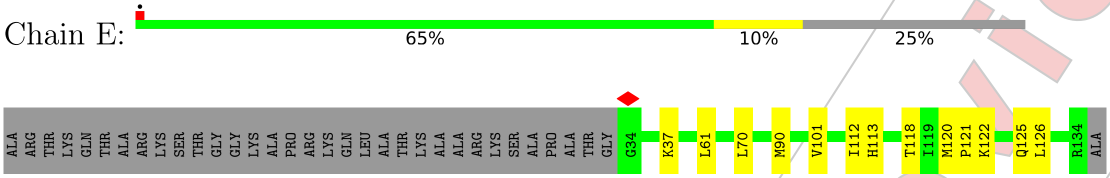


• Molecule 3: Histone H4

Chain B:

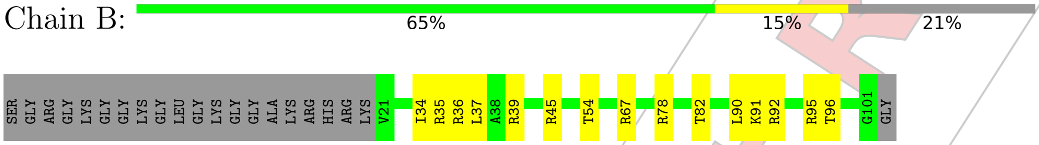


• Molecule 3: Histone H4

Chain F:

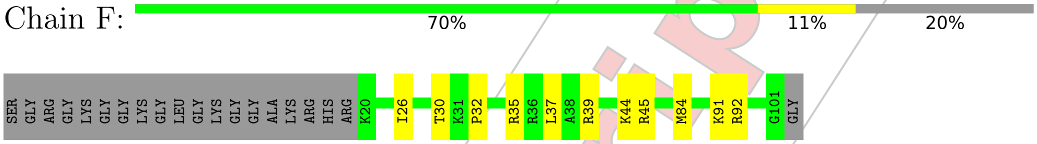


• Molecule 4: Histone H2A type 1

Chain C:

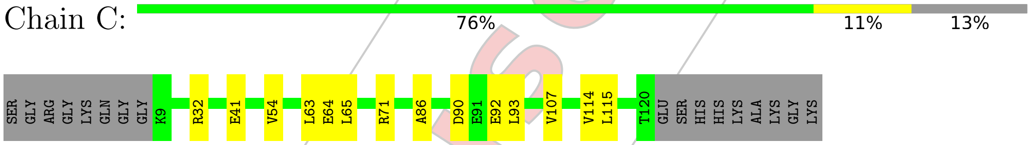


• Molecule 4: Histone H2A type 1

Chain G:

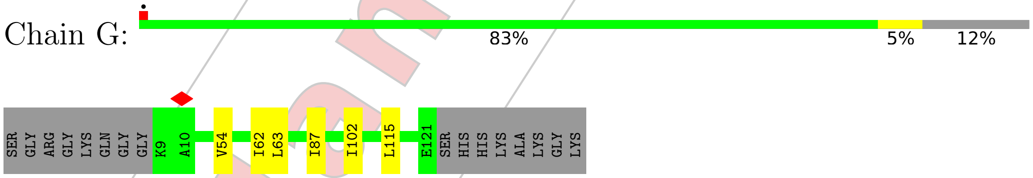


• Molecule 5: Histone H2B type 1-J

Chain D:

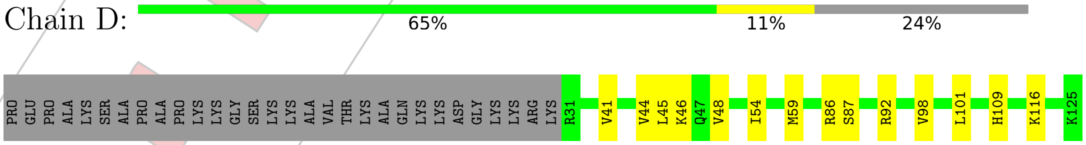


• Molecule 5: Histone H2B type 1-J

Chain H:

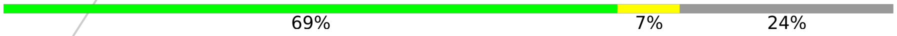


For Manuscript Review

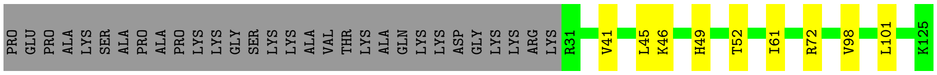


• Molecule 6: DNA (147-MER)

Chain I:

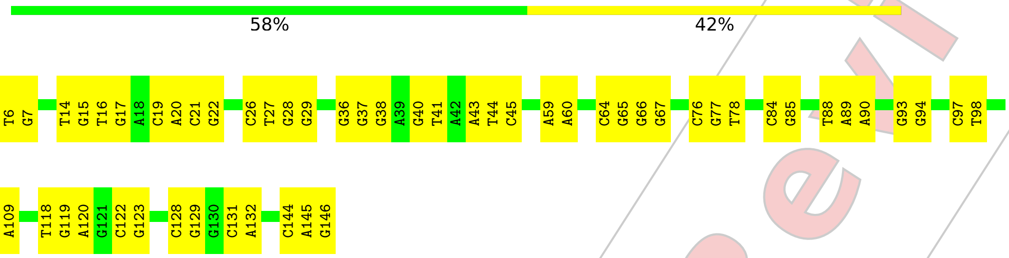


• Molecule 7: DNA (147-MER)

Chain J:

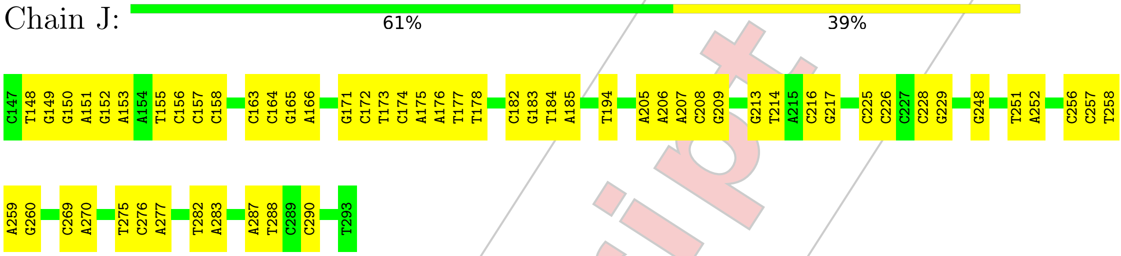


• Molecule 8: Baculoviral IAP repeat-containing protein 5

Chain M:

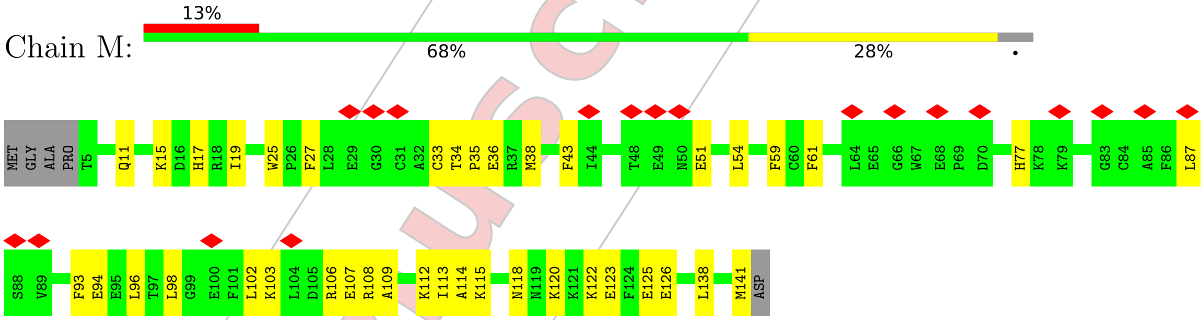


• Molecule 9: Inner centromere protein

Chain N:

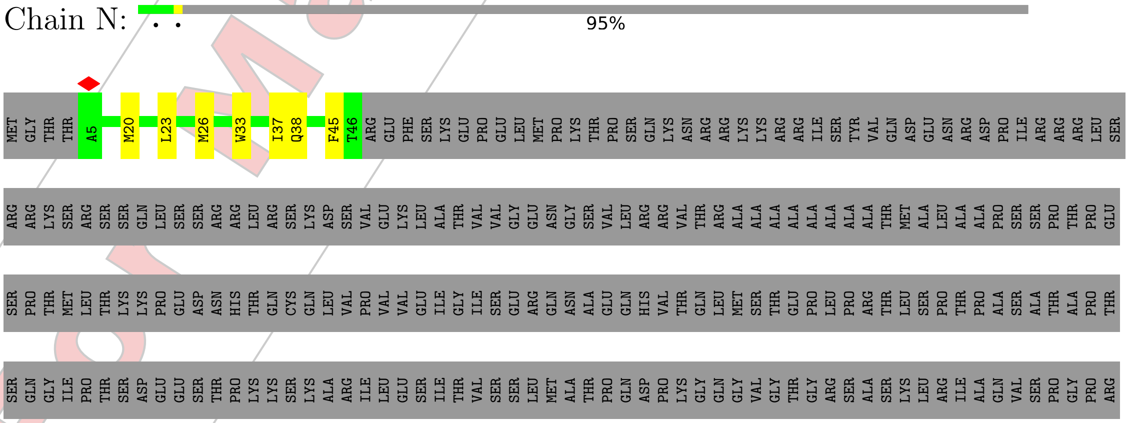


For Manuscript Review

ASP SER PRO ALA PHE PRO ASP SER PRO TRP ARG GLU ARG VAL LEU ALA PRO ILE LEU PRO ASP ASN PHE SER THR PRO THR GLY SER ARG THR ASP SER GLN SER VAL ARG HIS SER PRO ILE ALA PRO SER SER PRO SER PRO GLN VAL LEU ALA GLN LYS TYR SER LEU VAL ALA LYS

GLN GLU SER VAL VAL ARG ARG ALA SER ARG ARG LEU ALA LYS LYS THR ALA GLU GLU PRO ALA ALA SER GLY ARG ILE ILE CYS HIS SER TYR LEU GLU ARG LEU LEU ASN VAL GLU VAL PRO GLN LYS VAL GLY SER GLU GLN LYS GLU PRO PRO GLU GLU ALA GLU PRO VAL ALA ALA

ALA GLU PRO GLU VAL PRO GLU ASN ASN GLY ASN ASN SER TRP PRO HIS ASN ASP THR GLU ILE ALA ASN SER THR PRO ASN PRO LYS PRO ALA ALA SER SER PRO GLU THR PRO SER ALA GLY GLN GLN GLU ALA LYS THR ASP GLN ALA ASP GLY PRO ARG GLU PRO PRO GLN SER ALA

ARG ARG LYS ARG SER TYR LYS GLN ALA VAL SER GLU LEU ASP GLU GLU GLN HIS LEU GLU ASP GLU GLU LEU GLN PRO PRO ARG SER LYS THR PRO SER SER PRO CYS PRO ALA SER LYS VAL VAL ARG PRO LEU ARG THR PHE LEU HIS THR VAL GLN ARG ASN GLN MET LEU MET THR

PRO THR SER ALA PRO ARG SER VAL MET LYS SER PHE ILE LYS ARG ASN THR PRO LEU ARG MET ASP PRO LYS CYS SER PHE VAL GLU LYS GLU ARG GLN ARG LEU GLU ASN LEU ARG ARG LYS GLU GLU ALA GLU GLN LEU ARG ARG GLN LYS VAL GLU GLU ASP LYS ARG ARG ARG LEU

GLU GLU VAL LYS LEU LYS ARG GLU GLU ARG LEU ARG LYS VAL LEU GLN ALA ARG GLU ARG VAL GLU GLN MET LYS GLU GLU LYS LYS LYS GLN ILE GLU GLN LYS PHE ALA GLN ILE ASP GLU LYS THR GLU LYS ALA LYS GLU GLU ARG LEU ALA GLU GLU LYS ALA LYS LYS LYS ALA

ALA ALA LYS LYS MET GLU GLU VAL GLU ALA ARG ARG LYS GLN GLU GLU GLU ALA ARG ARG LEU ARG TRP LEU GLN GLN GLU GLU GLU GLU ARG ARG HIS GLN GLU LEU LEU GLN LYS LYS LYS GLU GLU GLU GLN GLU ARG LEU ARG LYS ALA ALA GLU ALA LYS ARG LEU ALA GLU GLN

ARG GLU GLN GLU ARG ARG GLU GLN GLU ARG ARG GLU GLN GLU ARG ARG GLU GLN GLU ARG ARG GLU GLN GLU ARG ARG GLU GLN GLU ARG GLN LEU ALA GLU GLN GLU ARG ARG ARG GLU GLN GLU ARG LEU GLN ALA GLU ARG GLU LEU GLN GLU ARG GLU LYS ALA LEU ARG LEU GLN

LYS GLU GLN LEU GLN ARG GLU LEU GLU GLU LYS LYS LYS LYS GLU GLU GLN GLN ARG LEU ALA GLU ARG GLN LEU GLN GLU GLU GLN GLU LYS LYS ALA LYS GLU ALA ALA GLY ALA SER LYS ALA LEU ASN VAL THR VAL ASP VAL GLN SER PRO ALA CYS THR SER TYR GLN MET THR

PRO GLN GLY HIS ARG ALA PRO PRO LYS ILE ASN PRO ASP ASN TYR GLY MET ASP LEU ASN SER ASP ASP SER THR ASP ASP GLU ALA HIS PRO ARG LYS PRO ILE PRO THR TRP ALA ARG GLY THR PRO LEU SER GLN ALA ILE ILE HIS GLN TYR TYR HIS PRO PRO ASN LEU LEU GLU

LEU PHE GLY THR ILE LEU PRO LEU ASP LEU GLU ASP ILE PHE LYS LYS SER LYS PRO ARG TYR HIS LYS ARG THR SER SER ALA VAL TRP ASN SER PRO PRO LEU GLN GLY ALA ARG VAL PRO SER SER LEU ALA TYR SER LEU LYS LYS HIS

For Manuscript Review

# 4 Experimental information (i)

| Property | Value | Source |
|---|---|---|
| EM reconstruction method | SINGLE PARTICLE | Depositor |
| Imposed symmetry | POINT, Not provided | |
| Number of particles used | 75653 | Depositor |
| Resolution determination method | FSC 0.143 CUT-OFF | Depositor |
| CTF correction method | PHASE FLIPPING AND AMPLITUDE CORRECTION | Depositor |
| Microscope | TFS KRIOS | Depositor |
| Voltage (kV) | 300 | Depositor |
| Electron dose ($e^-/\text{Å}^2$) | 40 | Depositor |
| Minimum defocus (nm) | 500 | Depositor |
| Maximum defocus (nm) | 2600 | Depositor |
| Magnification | Not provided | |
| Image detector | FEI FALCON IV (4k x 4k) | Depositor |
| Maximum map value | 0.903 | Depositor |
| Minimum map value | -0.325 | Depositor |
| Average map value | -0.000 | Depositor |
| Map value standard deviation | 0.029 | Depositor |
| Recommended contour level | 0.061 | Depositor |
| Map size (Å) | 279.168, 279.168, 279.168 | wwPDB |
| Map dimensions | 384, 384, 384 | wwPDB |
| Map angles (°) | 90.0, 90.0, 90.0 | wwPDB |
| Pixel spacing (Å) | 0.727, 0.727, 0.727 | Depositor |

For Manuscript Review

# 5 Model quality (i)

## 5.1 Standard geometry (i)

The Z score for a bond length (or angle) is the number of standard deviations the observed value is removed from the expected value. A bond length (or angle) with $|Z| > 5$ is considered an outlier worth inspection. RMSZ is the root-mean-square of all Z scores of the bond lengths (or angles).

| Mol | Chain | Bond lengths | | Bond angles | |
|---|---|---|---|---|---|
| | | RMSZ | $\#|Z| > 5$ | RMSZ | $\#|Z| > 5$ |
| 1 | L | 0.07 | 0/107 | 0.31 | 0/139 |
| 1 | O | 0.27 | 0/641 | 0.52 | 0/857 |
| 2 | A | 0.14 | 0/823 | 0.23 | 0/1104 |
| 2 | E | 0.15 | 0/843 | 0.26 | 0/1130 |
| 3 | B | 0.15 | 0/655 | 0.25 | 0/878 |
| 3 | F | 0.15 | 0/664 | 0.27 | 0/889 |
| 4 | C | 0.13 | 0/872 | 0.25 | 0/1174 |
| 4 | G | 0.13 | 0/881 | 0.22 | 0/1186 |
| 5 | D | 0.13 | 0/757 | 0.23 | 0/1015 |
| 5 | H | 0.13 | 0/757 | 0.22 | 0/1015 |
| 6 | I | 0.20 | 0/3408 | 0.41 | 0/5263 |
| 7 | J | 0.21 | 0/3349 | 0.37 | 0/5162 |
| 8 | M | 0.15 | 0/1147 | 0.46 | 0/1542 |
| 9 | N | 0.13 | 0/352 | 0.42 | 0/474 |
| All | All | 0.18 | 0/15256 | 0.35 | 0/21828 |

There are no bond length outliers.

There are no bond angle outliers.

There are no chirality outliers.

There are no planarity outliers.

## 5.2 Too-close contacts (i)

In the following table, the Non-H and H(model) columns list the number of non-hydrogen atoms and hydrogen atoms in the chain respectively. The H(added) column lists the number of hydrogen atoms added and optimized by MolProbity. The Clashes column lists the number of clashes within the asymmetric unit, whereas Symm-Clashes lists symmetry-related clashes.

| Mol | Chain | Non-H | H(model) | H(added) | Clashes | Symm-Clashes |
|---|---|---|---|---|---|---|
| 1 | L | 108 | 0 | 120 | 4 | 0 |
| 1 | O | 633 | 0 | 663 | 12 | 0 |

*Continued on next page...*

For Manuscript Review

For Manuscript Review

*Continued from previous page...*

| Mol | Chain | Non-H | H(model) | H(added) | Clashes | Symm-Clashes |
|---|---|---|---|---|---|---|
| 2 | A | 811 | 0 | 853 | 18 | 0 |
| 2 | E | 831 | 0 | 878 | 15 | 0 |
| 3 | B | 648 | 0 | 693 | 17 | 0 |
| 3 | F | 657 | 0 | 706 | 18 | 0 |
| 4 | C | 862 | 0 | 935 | 14 | 0 |
| 4 | G | 871 | 0 | 941 | 7 | 0 |
| 5 | D | 746 | 0 | 771 | 12 | 0 |
| 5 | H | 746 | 0 | 771 | 14 | 0 |
| 6 | I | 3034 | 0 | 1649 | 46 | 0 |
| 7 | J | 2990 | 0 | 1652 | 42 | 0 |
| 8 | M | 1118 | 0 | 1091 | 31 | 0 |
| 9 | N | 346 | 0 | 334 | 8 | 0 |
| All | All | 14401 | 0 | 12057 | 206 | 0 |

The all-atom clashscore is defined as the number of clashes found per 1000 atoms (including hydrogen atoms). The all-atom clashscore for this structure is 8.

All (206) close contacts within the same asymmetric unit are listed below, sorted by their clash magnitude.

| Atom-1 | Atom-2 | Interatomic distance (Å) | Clash overlap (Å) |
|---|---|---|---|
| 2:E:118:THR:HG22 | 3:F:45:ARG:HB3 | 1.56 | 0.85 |
| 2:A:118:THR:OG1 | 3:B:45:ARG:HD3 | 1.81 | 0.80 |
| 3:F:92:ARG:HH21 | 5:H:101:LEU:CD2 | 1.96 | 0.77 |
| 1:O:28:PHE:O | 1:O:32:VAL:HG23 | 1.83 | 0.77 |
| 3:F:92:ARG:HH21 | 5:H:101:LEU:HD23 | 1.49 | 0.75 |
| 2:E:118:THR:CG2 | 3:F:45:ARG:HE | 2.02 | 0.72 |
| 4:C:115:LEU:HD13 | 3:F:44:LYS:HB2 | 1.76 | 0.68 |
| 2:E:118:THR:HG22 | 3:F:45:ARG:HE | 1.59 | 0.67 |
| 1:L:9:ARG:NH1 | 4:C:92:GLU:OE1 | 2.22 | 0.66 |
| 3:B:78:ARG:HD2 | 7:J:248:DG:H5’ | 1.77 | 0.65 |
| 6:I:144:DC:H2” | 6:I:145:DA:C8 | 2.32 | 0.64 |
| 1:L:9:ARG:HD2 | 4:C:92:GLU:OE1 | 1.98 | 0.64 |
| 6:I:118:DT:H2” | 6:I:119:DG:C8 | 2.33 | 0.64 |
| 2:E:70:LEU:HD11 | 3:F:26:ILE:HD12 | 1.81 | 0.63 |
| 5:H:49:HIS:HB2 | 5:H:52:THR:OG1 | 1.99 | 0.62 |
| 8:M:15:LYS:HE2 | 8:M:19:ILE:HD11 | 1.80 | 0.62 |
| 7:J:287:DA:H2’ | 7:J:288:DT:H71 | 1.81 | 0.62 |
| 4:C:65:LEU:HB3 | 4:C:86:ALA:HB1 | 1.82 | 0.62 |
| 7:J:175:DA:H2” | 7:J:176:DA:C8 | 2.35 | 0.61 |
| 6:I:128:DC:H2” | 6:I:129:DG:N7 | 2.16 | 0.60 |
| 2:E:118:THR:HG22 | 3:F:45:ARG:CB | 2.31 | 0.60 |

*Continued on next page...*

Continued from previous page...

| Atom-1 | Atom-2 | Interatomic distance (Å) | Clash overlap (Å) |
|---|---|---|---|
| 3:B:90:LEU:HB3 | 3:B:95:ARG:HB2 | 1.83 | 0.60 |
| 8:M:122:LYS:HA | 8:M:125:GLU:HG3 | 1.85 | 0.59 |
| 6:I:77:DG:H2” | 6:I:78:DT:H72 | 1.85 | 0.58 |
| 2:A:61:LEU:HD22 | 3:B:36:ARG:HD2 | 1.84 | 0.58 |
| 6:I:19:DC:H2” | 6:I:20:DA:C8 | 2.39 | 0.58 |
| 7:J:155:DT:H2’ | 7:J:156:DC:C6 | 2.38 | 0.57 |
| 8:M:61:PHE:HE2 | 8:M:108:ARG:HH12 | 1.52 | 0.57 |
| 2:E:90:MET:HE2 | 2:E:90:MET:HA | 1.85 | 0.57 |
| 6:I:37:DG:H2” | 6:I:38:DG:N7 | 2.20 | 0.57 |
| 5:D:54:ILE:HG21 | 5:D:59:MET:HE2 | 1.85 | 0.57 |
| 2:E:61:LEU:HD12 | 3:F:37:LEU:HD23 | 1.85 | 0.57 |
| 6:I:26:DC:H2” | 6:I:27:DT:C5 | 2.40 | 0.56 |
| 3:F:92:ARG:NH2 | 5:H:101:LEU:CD2 | 2.67 | 0.56 |
| 7:J:156:DC:H2” | 7:J:157:DC:H5” | 1.88 | 0.56 |
| 7:J:205:DA:H2” | 7:J:206:DA:H5” | 1.86 | 0.56 |
| 3:B:91:LYS:HG3 | 3:B:96:THR:HG22 | 1.86 | 0.56 |
| 6:I:88:DT:H2” | 6:I:89:DA:C8 | 2.41 | 0.56 |
| 2:E:120:MET:HE3 | 2:E:122:LYS:HE2 | 1.87 | 0.56 |
| 6:I:76:DC:H2” | 6:I:77:DG:C8 | 2.42 | 0.55 |
| 6:I:43:DA:H2” | 6:I:44:DT:H5” | 1.88 | 0.55 |
| 2:A:118:THR:OG1 | 3:B:45:ARG:CD | 2.51 | 0.55 |
| 4:C:41:GLU:HG2 | 5:D:87:SER:HB2 | 1.88 | 0.55 |
| 7:J:171:DG:H5’ | 7:J:171:DG:C8 | 2.42 | 0.54 |
| 2:A:61:LEU:HD13 | 3:B:36:ARG:C | 2.32 | 0.54 |
| 4:C:63:LEU:HD13 | 5:D:45:LEU:HB2 | 1.91 | 0.53 |
| 6:I:4:DG:H2” | 6:I:5:DA:H5’ | 1.90 | 0.53 |
| 1:O:21:LEU:O | 1:O:25:LEU:HG | 2.09 | 0.53 |
| 8:M:108:ARG:C | 8:M:112:LYS:HZ2 | 2.17 | 0.53 |
| 7:J:165:DG:H2” | 7:J:166:DA:C8 | 2.44 | 0.52 |
| 6:I:108:DT:H2” | 6:I:109:DA:N7 | 2.24 | 0.52 |
| 7:J:150:DG:H2” | 7:J:151:DA:C8 | 2.45 | 0.52 |
| 7:J:166:DA:C8 | 7:J:166:DA:H5’ | 2.45 | 0.52 |
| 3:B:92:ARG:HH22 | 5:D:101:LEU:HD23 | 1.75 | 0.52 |
| 7:J:228:DC:H2” | 7:J:229:DG:C8 | 2.43 | 0.52 |
| 1:O:14:ASN:O | 1:O:15:SER:C | 2.53 | 0.52 |
| 6:I:1:DC:H1’ | 6:I:2:DA:C5 | 2.44 | 0.52 |
| 5:D:116:LYS:NZ | 5:D:116:LYS:HB2 | 2.24 | 0.52 |
| 1:O:50:VAL:HG21 | 8:M:113:ILE:HD13 | 1.92 | 0.52 |
| 3:F:84:MET:HE3 | 3:F:84:MET:HA | 1.92 | 0.52 |
| 6:I:40:DG:H2’ | 6:I:41:DT:H71 | 1.91 | 0.51 |
| 6:I:118:DT:H2” | 6:I:119:DG:N7 | 2.25 | 0.51 |

Continued on next page...

For Manuscript Review

*Continued from previous page...*

| Atom-1 | Atom-2 | Interatomic distance (Å) | Clash overlap (Å) |
|---|---|---|---|
| 5:H:49:HIS:CB | 5:H:52:THR:OG1 | 2.58 | 0.51 |
| 6:I:28:DG:H1' | 6:I:29:DG:C8 | 2.46 | 0.51 |
| 7:J:206:DA:H2" | 7:J:207:DA:C8 | 2.46 | 0.51 |
| 3:B:67:ARG:HH11 | 3:B:67:ARG:HG2 | 1.76 | 0.51 |
| 4:G:63:LEU:HD13 | 5:H:45:LEU:HB2 | 1.92 | 0.51 |
| 9:N:37:ILE:HD12 | 9:N:38:GLN:N | 2.26 | 0.51 |
| 2:A:73:GLU:O | 2:A:76:GLN:HG2 | 2.10 | 0.50 |
| 7:J:157:DC:H2" | 7:J:158:DC:H5' | 1.93 | 0.50 |
| 6:I:108:DT:H2" | 6:I:109:DA:C8 | 2.46 | 0.50 |
| 9:N:33:TRP:CH2 | 9:N:37:ILE:HG12 | 2.46 | 0.50 |
| 6:I:104:DT:H2" | 6:I:105:DG:H5" | 1.93 | 0.50 |
| 1:O:70:TRP:HZ2 | 8:M:96:LEU:HB2 | 1.77 | 0.50 |
| 6:I:122:DC:H2" | 6:I:123:DG:C8 | 2.47 | 0.50 |
| 6:I:16:DT:H2" | 6:I:17:DG:C8 | 2.47 | 0.49 |
| 1:O:70:TRP:HD1 | 8:M:98:LEU:HA | 1.77 | 0.49 |
| 3:F:92:ARG:HH21 | 5:H:101:LEU:HD22 | 1.74 | 0.49 |
| 7:J:258:DT:H2" | 7:J:259:DA:C8 | 2.47 | 0.49 |
| 7:J:152:DG:H2" | 7:J:153:DA:C8 | 2.48 | 0.49 |
| 2:A:118:THR:OG1 | 3:B:45:ARG:HB3 | 2.13 | 0.49 |
| 2:A:61:LEU:HD13 | 3:B:36:ARG:HB3 | 1.95 | 0.48 |
| 2:A:85:GLN:HG3 | 3:B:82:THR:HA | 1.94 | 0.48 |
| 6:I:145:DA:H2" | 6:I:146:DG:N7 | 2.28 | 0.48 |
| 6:I:14:DT:H2" | 6:I:15:DG:C8 | 2.48 | 0.48 |
| 7:J:228:DC:H2" | 7:J:229:DG:H8 | 1.79 | 0.48 |
| 4:C:107:VAL:HG11 | 2:E:101:VAL:HG11 | 1.96 | 0.48 |
| 2:A:118:THR:HG1 | 3:B:45:ARG:HD3 | 1.77 | 0.48 |
| 8:M:38:MET:HB3 | 8:M:43:PHE:HB2 | 1.94 | 0.48 |
| 7:J:182:DC:H2" | 7:J:183:DG:C8 | 2.48 | 0.48 |
| 8:M:61:PHE:CE2 | 8:M:112:LYS:HB3 | 2.48 | 0.48 |
| 8:M:77:HIS:CD2 | 8:M:87:LEU:HD21 | 2.49 | 0.48 |
| 2:A:46:VAL:HG21 | 7:J:229:DG:H3' | 1.96 | 0.48 |
| 3:F:92:ARG:NH2 | 5:H:101:LEU:HD22 | 2.29 | 0.48 |
| 4:C:114:VAL:HG11 | 2:E:112:ILE:HG12 | 1.96 | 0.47 |
| 7:J:216:DC:H2" | 7:J:217:DG:C8 | 2.49 | 0.47 |
| 4:G:54:VAL:HG21 | 5:H:98:VAL:HG21 | 1.96 | 0.47 |
| 7:J:177:DT:C2' | 7:J:178:DT:H71 | 2.44 | 0.47 |
| 6:I:119:DG:H1' | 6:I:120:DA:N7 | 2.29 | 0.47 |
| 7:J:275:DT:H2" | 7:J:276:DC:C5 | 2.48 | 0.47 |
| 8:M:120:LYS:HG3 | 9:N:26:MET:HE1 | 1.95 | 0.47 |
| 3:F:30:THR:HB | 3:F:32:PRO:HD2 | 1.96 | 0.47 |
| 8:M:115:LYS:HE2 | 8:M:115:LYS:HA | 1.96 | 0.47 |

*Continued on next page...*

For Manuscript Review

*Continued from previous page...*

| Atom-1 | Atom-2 | Interatomic distance (Å) | Clash overlap (Å) |
|---|---|---|---|
| 6:I:6:DT:H2” | 6:I:7:DG:H8 | 1.80 | 0.47 |
| 6:I:0:DA:H1’ | 6:I:1:DC:C4 | 2.50 | 0.46 |
| 6:I:93:DG:H2” | 6:I:94:DG:H5” | 1.98 | 0.46 |
| 7:J:269:DC:H2” | 7:J:270:DA:C8 | 2.50 | 0.46 |
| 7:J:184:DT:H2” | 7:J:185:DA:N7 | 2.30 | 0.46 |
| 4:G:102:ILE:HG23 | 5:H:61:ILE:HD13 | 1.98 | 0.46 |
| 8:M:138:LEU:HA | 8:M:141:MET:HE2 | 1.97 | 0.46 |
| 4:C:32:ARG:HD3 | 6:I:29:DG:OP2 | 2.16 | 0.46 |
| 7:J:194:DT:H6 | 7:J:194:DT:H5” | 1.81 | 0.46 |
| 8:M:51:GLU:HB3 | 8:M:54:LEU:HB2 | 1.98 | 0.46 |
| 6:I:66:DG:H2” | 6:I:67:DG:C8 | 2.50 | 0.45 |
| 6:I:0:DA:H1’ | 6:I:1:DC:C5 | 2.51 | 0.45 |
| 6:I:36:DG:H2” | 6:I:37:DG:N7 | 2.32 | 0.45 |
| 7:J:172:DC:H2” | 7:J:173:DT:C5 | 2.51 | 0.45 |
| 4:C:71:ARG:HG2 | 4:C:71:ARG:HH11 | 1.82 | 0.45 |
| 1:L:7:SER:HB2 | 5:D:109:HIS:CD2 | 2.51 | 0.45 |
| 1:O:70:TRP:CZ2 | 8:M:96:LEU:HB2 | 2.51 | 0.45 |
| 7:J:173:DT:H2” | 7:J:174:DC:C6 | 2.51 | 0.45 |
| 8:M:108:ARG:HB3 | 8:M:108:ARG:CZ | 2.47 | 0.45 |
| 6:I:36:DG:H2” | 6:I:37:DG:C8 | 2.52 | 0.44 |
| 7:J:175:DA:H2” | 7:J:176:DA:H8 | 1.81 | 0.44 |
| 1:O:21:LEU:HD12 | 1:O:25:LEU:HD21 | 1.99 | 0.44 |
| 4:G:62:ILE:HD13 | 4:G:87:ILE:HD11 | 1.99 | 0.44 |
| 8:M:103:LYS:O | 8:M:107:GLU:HG2 | 2.17 | 0.44 |
| 3:B:92:ARG:CZ | 3:B:92:ARG:HB2 | 2.48 | 0.44 |
| 5:D:86:ARG:HH21 | 5:D:92:ARG:HH22 | 1.65 | 0.44 |
| 8:M:123:GLU:O | 8:M:126:GLU:HG2 | 2.17 | 0.44 |
| 7:J:259:DA:H2” | 7:J:260:DG:H8 | 1.82 | 0.44 |
| 7:J:251:DT:H1’ | 7:J:252:DA:H5’ | 1.99 | 0.44 |
| 5:D:46:LYS:HD3 | 5:D:46:LYS:HA | 1.73 | 0.44 |
| 6:I:84:DC:H2” | 6:I:85:DG:C8 | 2.52 | 0.44 |
| 8:M:93:PHE:CD1 | 8:M:94:GLU:N | 2.86 | 0.44 |
| 7:J:163:DC:H2” | 7:J:164:DC:C5 | 2.53 | 0.43 |
| 9:N:33:TRP:O | 9:N:37:ILE:HG13 | 2.18 | 0.43 |
| 1:L:4:ARG:HH21 | 5:D:44:VAL:HG13 | 1.83 | 0.43 |
| 8:M:103:LYS:HG2 | 8:M:106:ARG:HH12 | 1.82 | 0.43 |
| 8:M:109:ALA:HA | 8:M:112:LYS:HD2 | 1.99 | 0.43 |
| 6:I:144:DC:H2” | 6:I:145:DA:N7 | 2.33 | 0.43 |
| 6:I:101:DA:H5” | 6:I:101:DA:H8 | 1.83 | 0.43 |
| 7:J:176:DA:H2’ | 7:J:177:DT:H71 | 2.00 | 0.43 |
| 6:I:89:DA:H1’ | 6:I:90:DA:C8 | 2.54 | 0.43 |

*Continued on next page...*

For Manuscript Review

*Continued from previous page...*

| Atom-1 | Atom-2 | Interatomic distance (Å) | Clash overlap (Å) |
|---|---|---|---|
| 7:J:290:DC:H6 | 7:J:290:DC:H2' | 1.70 | 0.43 |
| 2:A:118:THR:HG22 | 2:A:119:ILE:N | 2.33 | 0.43 |
| 6:I:59:DA:H2" | 6:I:60:DA:C8 | 2.54 | 0.43 |
| 6:I:16:DT:H2" | 6:I:17:DG:N7 | 2.34 | 0.43 |
| 5:H:72:ARG:HB3 | 5:H:101:LEU:HD13 | 2.01 | 0.43 |
| 7:J:225:DC:H2" | 7:J:226:DC:C5 | 2.54 | 0.43 |
| 9:N:26:MET:HE2 | 9:N:26:MET:HB3 | 1.89 | 0.43 |
| 8:M:59:PHE:HA | 8:M:108:ARG:HD2 | 1.99 | 0.42 |
| 1:O:17:ARG:NH2 | 9:N:45:PHE:HB3 | 2.35 | 0.42 |
| 9:N:20:MET:HA | 9:N:23:LEU:HD23 | 2.00 | 0.42 |
| 2:A:113:HIS:CG | 2:E:126:LEU:HD22 | 2.54 | 0.42 |
| 2:A:126:LEU:HD22 | 2:E:113:HIS:CG | 2.55 | 0.42 |
| 6:I:4:DG:H2" | 6:I:5:DA:C8 | 2.53 | 0.42 |
| 2:A:61:LEU:HD12 | 3:B:37:LEU:HD23 | 2.02 | 0.42 |
| 6:I:26:DC:H2" | 6:I:27:DT:C7 | 2.49 | 0.42 |
| 8:M:33:CYS:HB3 | 8:M:38:MET:HE1 | 2.00 | 0.42 |
| 6:I:5:DA:H1' | 6:I:6:DT:H5" | 2.02 | 0.42 |
| 6:I:28:DG:H1' | 6:I:29:DG:N7 | 2.34 | 0.42 |
| 6:I:44:DT:H2" | 6:I:45:DC:H5' | 2.02 | 0.42 |
| 3:F:91:LYS:HE3 | 3:F:91:LYS:HB3 | 1.89 | 0.42 |
| 7:J:208:DC:H2" | 7:J:209:DG:C8 | 2.55 | 0.42 |
| 7:J:176:DA:C2' | 7:J:177:DT:H71 | 2.50 | 0.42 |
| 2:E:37:LYS:HE2 | 2:E:37:LYS:HB2 | 1.82 | 0.42 |
| 2:A:117:VAL:HG13 | 4:G:115:LEU:HD22 | 2.01 | 0.41 |
| 3:B:35:ARG:O | 3:B:39:ARG:HG2 | 2.20 | 0.41 |
| 4:C:63:LEU:HD11 | 5:D:41:VAL:HG13 | 2.01 | 0.41 |
| 5:H:46:LYS:HA | 5:H:46:LYS:HD3 | 1.79 | 0.41 |
| 1:O:70:TRP:CZ3 | 1:O:71:LEU:HG | 2.55 | 0.41 |
| 4:C:54:VAL:HG21 | 5:D:98:VAL:HG21 | 2.02 | 0.41 |
| 4:C:90:ASP:OD1 | 4:C:93:LEU:HB2 | 2.20 | 0.41 |
| 2:A:107:THR:HG23 | 2:A:123:ASP:HB2 | 2.02 | 0.41 |
| 4:C:64:GLU:HB2 | 5:D:48:VAL:HG11 | 2.02 | 0.41 |
| 4:G:87:ILE:HD13 | 4:G:87:ILE:HA | 1.87 | 0.41 |
| 6:I:21:DC:H2" | 6:I:22:DG:H8 | 1.85 | 0.41 |
| 1:O:17:ARG:HH22 | 9:N:45:PHE:HB3 | 1.85 | 0.41 |
| 8:M:11:GLN:HB3 | 8:M:17:HIS:CE1 | 2.55 | 0.41 |
| 2:A:37:LYS:HB3 | 2:A:37:LYS:HE2 | 1.83 | 0.41 |
| 7:J:213:DG:H2" | 7:J:214:DT:C5 | 2.55 | 0.41 |
| 7:J:256:DC:H2" | 7:J:257:DC:C6 | 2.56 | 0.41 |
| 7:J:277:DA:H8 | 7:J:277:DA:H5" | 1.85 | 0.41 |
| 4:G:63:LEU:HD11 | 5:H:41:VAL:HG13 | 2.01 | 0.41 |

*Continued on next page...*

For Manuscript Review

*Continued from previous page...*

| Atom-1 | Atom-2 | Interatomic distance (Å) | Clash overlap (Å) |
|---|---|---|---|
| 6:I:97:DC:H2" | 6:I:98:DT:C6 | 2.55 | 0.41 |
| 8:M:34:THR:C | 8:M:38:MET:HE2 | 2.45 | 0.41 |
| 7:J:256:DC:H2" | 7:J:257:DC:C5 | 2.56 | 0.41 |
| 6:I:64:DC:H2" | 6:I:65:DG:C8 | 2.56 | 0.41 |
| 7:J:183:DG:H2" | 7:J:184:DT:C6 | 2.56 | 0.41 |
| 7:J:282:DT:H2" | 7:J:283:DA:C8 | 2.56 | 0.41 |
| 8:M:114:ALA:O | 8:M:118:ASN:OD1 | 2.38 | 0.41 |
| 3:B:34:ILE:HD13 | 3:B:54:THR:HB | 2.02 | 0.41 |
| 1:O:44:GLN:O | 1:O:47:LEU:HD12 | 2.21 | 0.41 |
| 8:M:25:TRP:CZ2 | 8:M:27:PHE:HB2 | 2.56 | 0.41 |
| 6:I:131:DC:H2" | 6:I:132:DA:H8 | 1.86 | 0.40 |
| 3:F:35:ARG:O | 3:F:39:ARG:HG2 | 2.21 | 0.40 |
| 3:F:92:ARG:NH2 | 5:H:101:LEU:HD23 | 2.26 | 0.40 |
| 8:M:25:TRP:CD1 | 8:M:35:PRO:HD3 | 2.56 | 0.40 |
| 8:M:36:GLU:H | 8:M:36:GLU:CD | 2.28 | 0.40 |
| 8:M:102:LEU:HD23 | 8:M:102:LEU:HA | 1.93 | 0.40 |
| 8:M:109:ALA:HA | 8:M:112:LYS:CD | 2.52 | 0.40 |
| 2:E:118:THR:CG2 | 3:F:45:ARG:NE | 2.78 | 0.40 |
| 2:A:65:LEU:HD23 | 2:A:65:LEU:HA | 1.90 | 0.40 |
| 2:E:121:PRO:O | 2:E:125:GLN:HG2 | 2.22 | 0.40 |
| 7:J:148:DT:H2" | 7:J:149:DG:N7 | 2.36 | 0.40 |

There are no symmetry-related clashes.

## 5.3 Torsion angles (i)

### 5.3.1 Protein backbone (i)

In the following table, the Percentiles column shows the percent Ramachandran outliers of the chain as a percentile score with respect to all PDB entries followed by that with respect to all EM entries.

The Analysed column shows the number of residues for which the backbone conformation was analysed, and the total number of residues.

| Mol | Chain | Analysed | Favoured | Allowed | Outliers | Percentiles | |
|---|---|---|---|---|---|---|---|
| 1 | L | 12/280 (4%) | 10 (83%) | 2 (17%) | 0 | 100 | 100 |
| 1 | O | 73/280 (26%) | 70 (96%) | 3 (4%) | 0 | 100 | 100 |
| 2 | A | 96/135 (71%) | 94 (98%) | 2 (2%) | 0 | 100 | 100 |
| 2 | E | 99/135 (73%) | 96 (97%) | 3 (3%) | 0 | 100 | 100 |

*Continued on next page...*

For Manuscript Review

*Continued from previous page...*

| Mol | Chain | Analysed | Favoured | Allowed | Outliers | Percentiles | |
|---|---|---|---|---|---|---|---|
| 3 | B | 79/102 (78%) | 75 (95%) | 4 (5%) | 0 | 100 | 100 |
| 3 | F | 80/102 (78%) | 79 (99%) | 1 (1%) | 0 | 100 | 100 |
| 4 | C | 110/129 (85%) | 107 (97%) | 3 (3%) | 0 | 100 | 100 |
| 4 | G | 111/129 (86%) | 108 (97%) | 3 (3%) | 0 | 100 | 100 |
| 5 | D | 93/125 (74%) | 91 (98%) | 2 (2%) | 0 | 100 | 100 |
| 5 | H | 93/125 (74%) | 91 (98%) | 2 (2%) | 0 | 100 | 100 |
| 8 | M | 135/142 (95%) | 131 (97%) | 4 (3%) | 0 | 100 | 100 |
| 9 | N | 40/918 (4%) | 39 (98%) | 1 (2%) | 0 | 100 | 100 |
| All | All | 1021/2602 (39%) | 991 (97%) | 30 (3%) | 0 | 100 | 100 |

There are no Ramachandran outliers to report.

### 5.3.2 Protein sidechains (i)

In the following table, the Percentiles column shows the percent sidechain outliers of the chain as a percentile score with respect to all PDB entries followed by that with respect to all EM entries.

The Analysed column shows the number of residues for which the sidechain conformation was analysed, and the total number of residues.

| Mol | Chain | Analysed | Rotameric | Outliers | Percentiles | |
|---|---|---|---|---|---|---|
| 1 | L | 12/242 (5%) | 12 (100%) | 0 | 100 | 100 |
| 1 | O | 69/242 (28%) | 69 (100%) | 0 | 100 | 100 |
| 2 | A | 86/110 (78%) | 86 (100%) | 0 | 100 | 100 |
| 2 | E | 88/110 (80%) | 88 (100%) | 0 | 100 | 100 |
| 3 | B | 67/78 (86%) | 67 (100%) | 0 | 100 | 100 |
| 3 | F | 68/78 (87%) | 68 (100%) | 0 | 100 | 100 |
| 4 | C | 87/98 (89%) | 87 (100%) | 0 | 100 | 100 |
| 4 | G | 88/98 (90%) | 88 (100%) | 0 | 100 | 100 |
| 5 | D | 81/104 (78%) | 81 (100%) | 0 | 100 | 100 |
| 5 | H | 81/104 (78%) | 81 (100%) | 0 | 100 | 100 |
| 8 | M | 120/123 (98%) | 120 (100%) | 0 | 100 | 100 |
| 9 | N | 39/817 (5%) | 39 (100%) | 0 | 100 | 100 |
| All | All | 886/2204 (40%) | 886 (100%) | 0 | 100 | 100 |

For Manuscript Review

There are no protein residues with a non-rotameric sidechain to report.

Sometimes sidechains can be flipped to improve hydrogen bonding and reduce clashes. All (7) such sidechains are listed below:

| Mol | Chain | Res | Type |
|---|---|---|---|
| 2 | A | 113 | HIS |
| 3 | B | 25 | ASN |
| 4 | C | 24 | GLN |
| 5 | D | 47 | GLN |
| 3 | F | 75 | HIS |
| 4 | G | 104 | GLN |
| 1 | O | 44 | GLN |

#### 5.3.3 RNA (i)

There are no RNA molecules in this entry.

## 5.4 Non-standard residues in protein, DNA, RNA chains (i)

There are no non-standard protein/DNA/RNA residues in this entry.

## 5.5 Carbohydrates (i)

There are no oligosaccharides in this entry.

## 5.6 Ligand geometry (i)

There are no ligands in this entry.

## 5.7 Other polymers (i)

There are no such residues in this entry.

## 5.8 Polymer linkage issues (i)

There are no chain breaks in this entry.

For Manuscript Review

# 6 Map visualisation (i)

This section contains visualisations of the EMDB entry EMD-54924. These allow visual inspection of the internal detail of the map and identification of artifacts.

Images derived from a raw map, generated by summing the deposited half-maps, are presented below the corresponding image components of the primary map to allow further visual inspection and comparison with those of the primary map.

## 6.1 Orthogonal projections (i)

### 6.1.1 Primary map

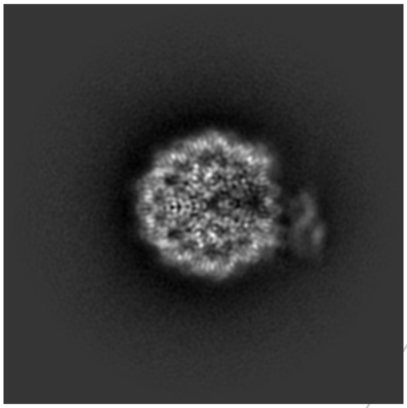

X

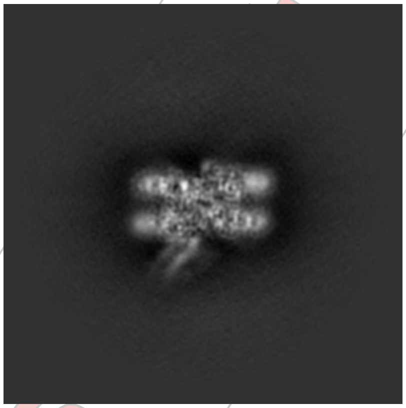

Y

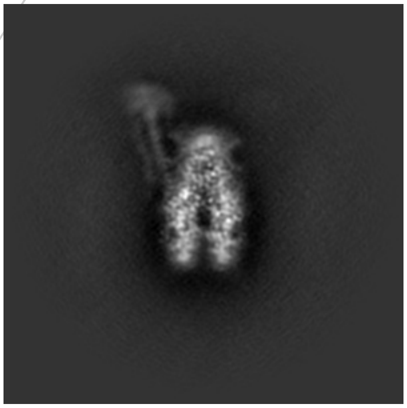

Z

### 6.1.2 Raw map

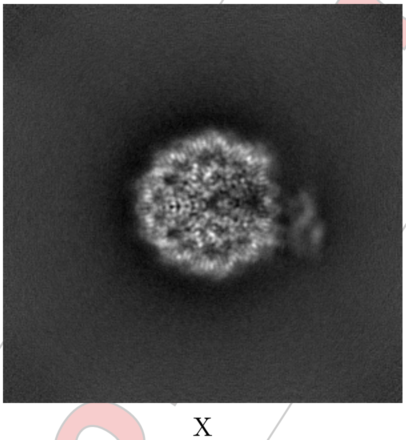

X

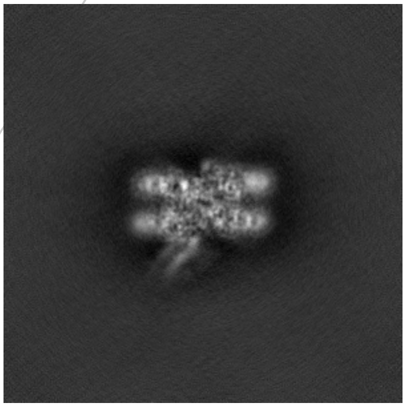

Y

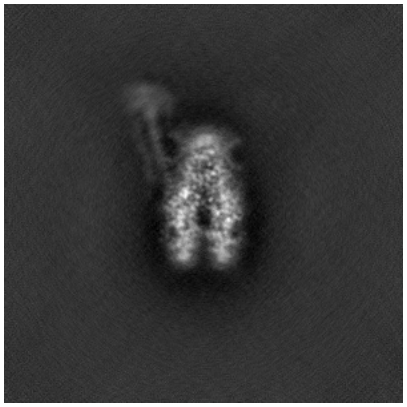

Z

The images above show the map projected in three orthogonal directions.

For Manuscript Review

## 6.2 Central slices (i)

### 6.2.1 Primary map

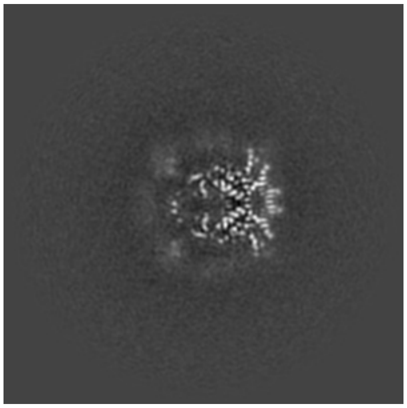
X Index: 192

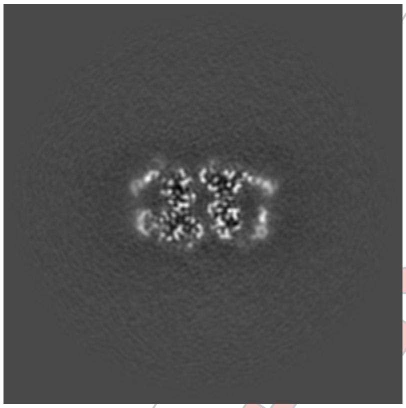
Y Index: 192

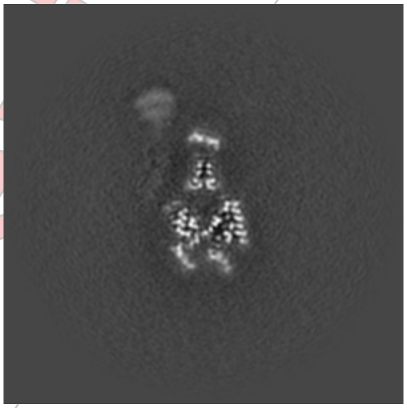
Z Index: 192

### 6.2.2 Raw map

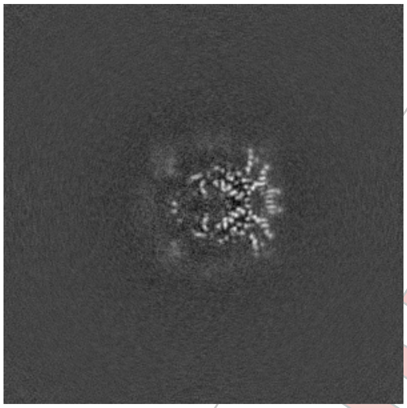
X Index: 192

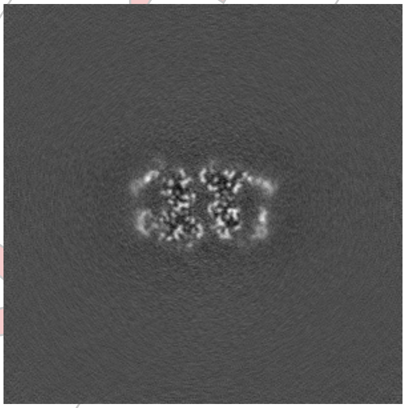
Y Index: 192

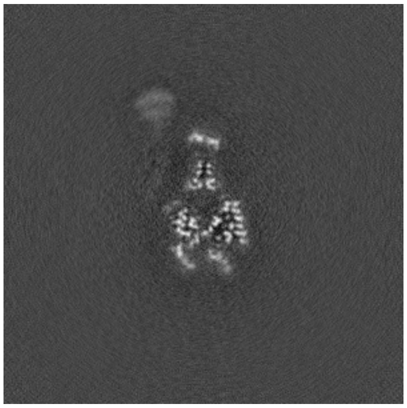
Z Index: 192

The images above show central slices of the map in three orthogonal directions.

For Manuscript Review

## 6.3 Largest variance slices (i)

### 6.3.1 Primary map

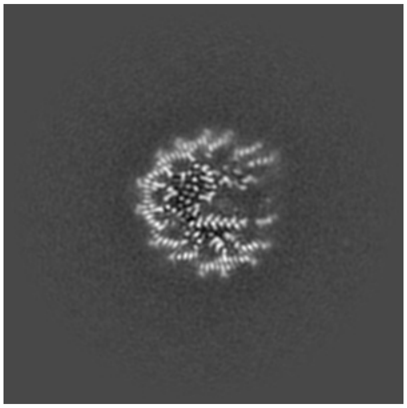

X Index: 209

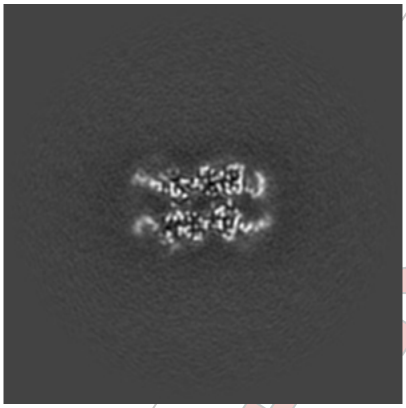

Y Index: 184

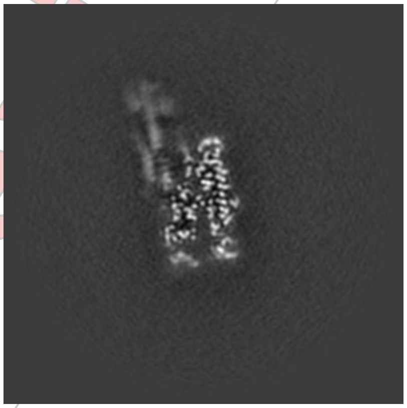

Z Index: 172

### 6.3.2 Raw map

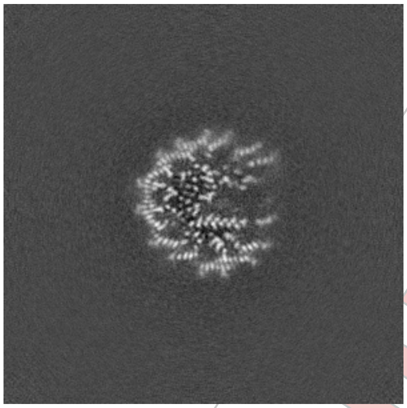

X Index: 209

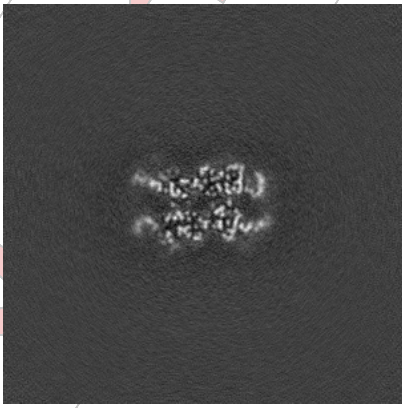

Y Index: 184

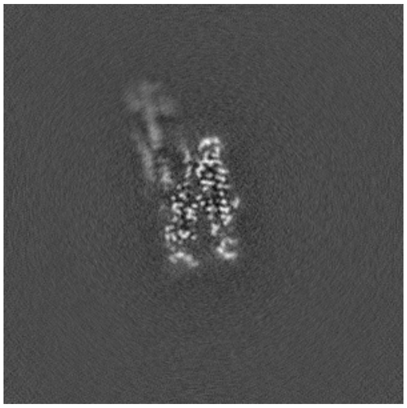

Z Index: 171

The images above show the largest variance slices of the map in three orthogonal directions.

For Manuscript Review

## 6.4 Orthogonal standard-deviation projections (False-color) (i)

### 6.4.1 Primary map

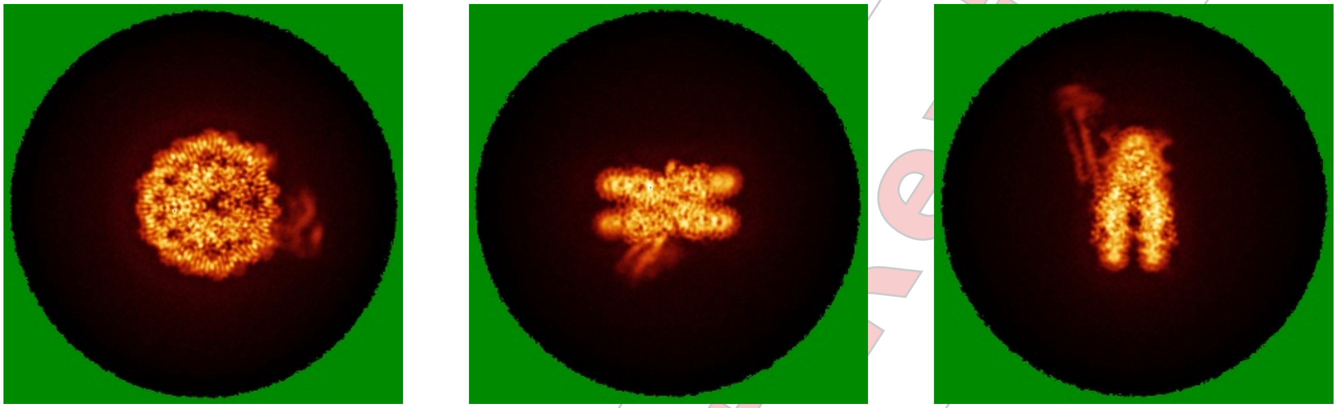

X Y Z

### 6.4.2 Raw map

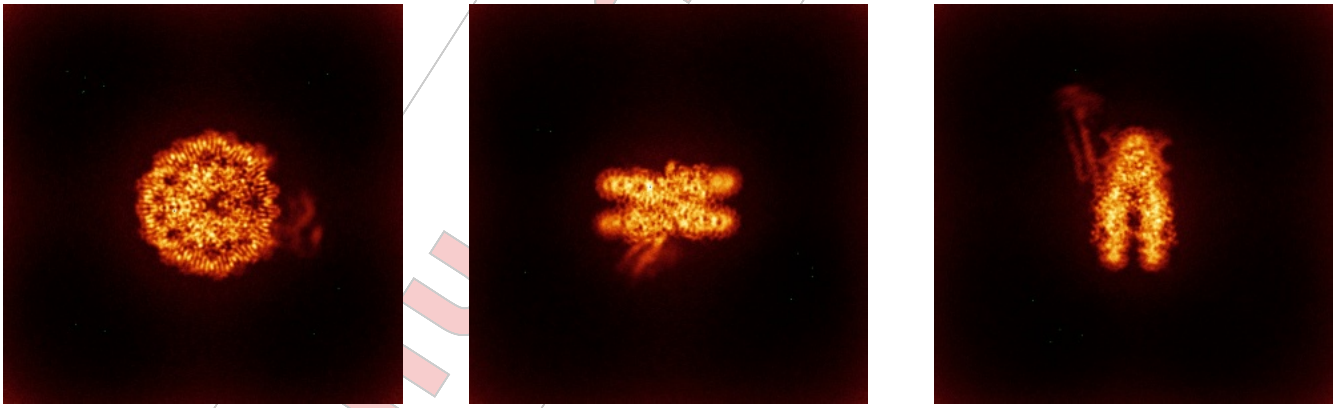

X Y Z

The images above show the map standard deviation projections with false color in three orthogonal directions. Minimum values are shown in green, max in blue, and dark to light orange shades represent small to large values respectively.

For Manuscript Review

## 6.5 Orthogonal surface views ⓘ

### 6.5.1 Primary map

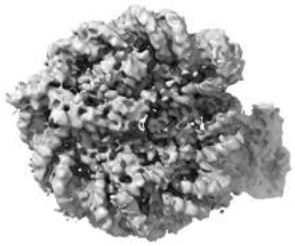

X

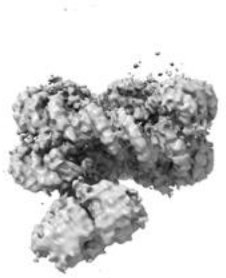

Y

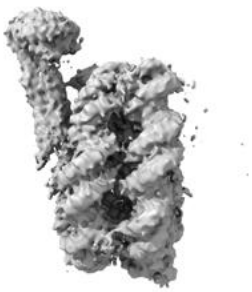

Z

The images above show the 3D surface view of the map at the recommended contour level 0.061. These images, in conjunction with the slice images, may facilitate assessment of whether an appropriate contour level has been provided.

### 6.5.2 Raw map

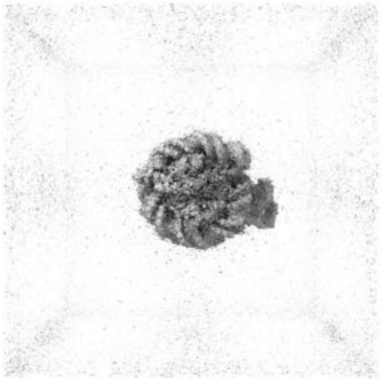

X

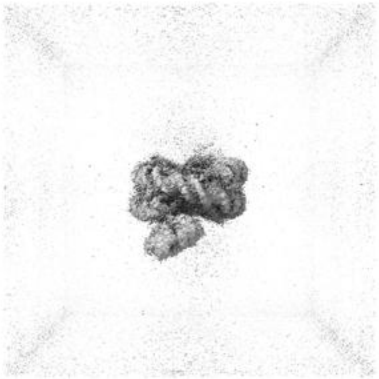

Y

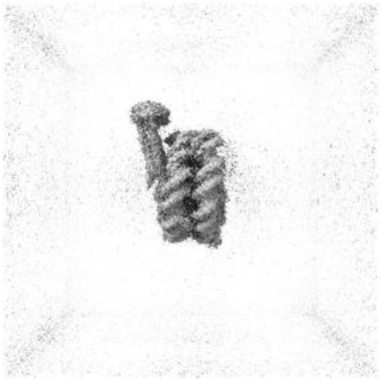

Z

These images show the 3D surface of the raw map. The raw map's contour level was selected so that its surface encloses the same volume as the primary map does at its recommended contour level.

## 6.6 Mask visualisation ⓘ

This section was not generated. No masks/segmentation were deposited.

For Manuscript Review

# 7 Map analysis (i)

This section contains the results of statistical analysis of the map.

## 7.1 Map-value distribution (i)

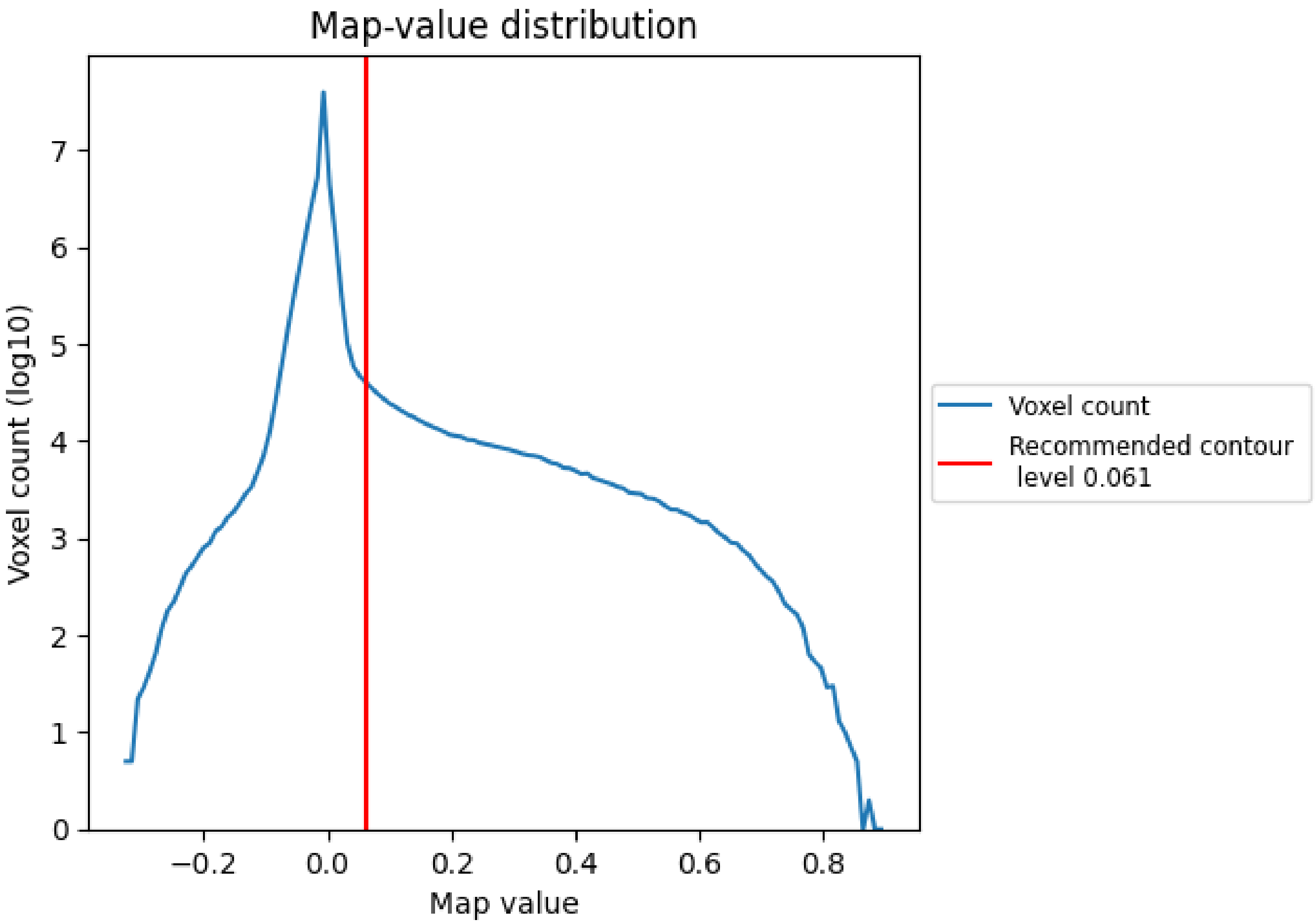


The map-value distribution is plotted in 128 intervals along the x-axis. The y-axis is logarithmic. A spike in this graph at zero usually indicates that the volume has been masked.

For Manuscript Review

## 7.2 Volume estimate (i)

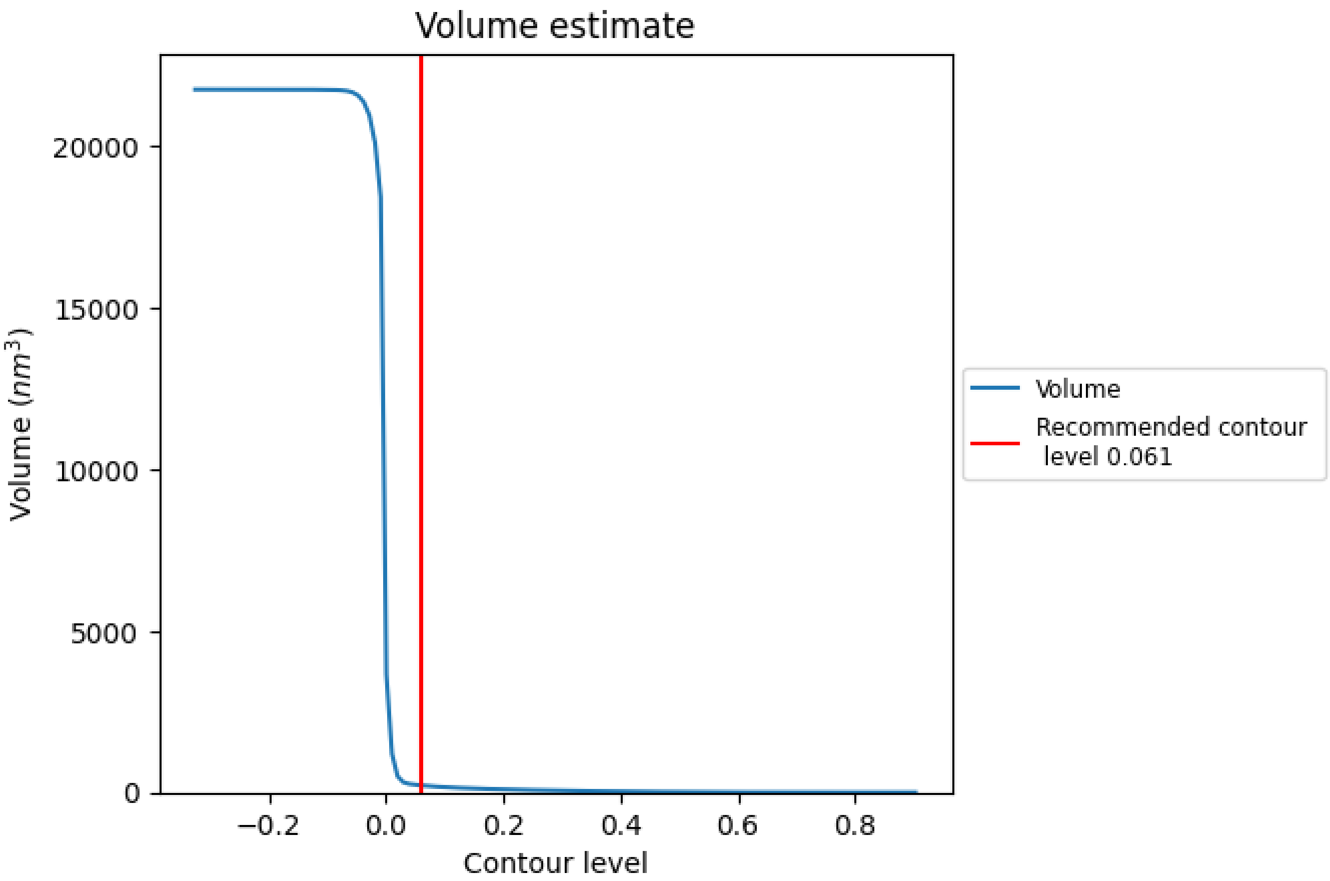


The volume at the recommended contour level is 216 $nm^3$; this corresponds to an approximate mass of 195 kDa.

The volume estimate graph shows how the enclosed volume varies with the contour level. The recommended contour level is shown as a vertical line and the intersection between the line and the curve gives the volume of the enclosed surface at the given level.

For Manuscript Review

## 7.3 Rotationally averaged power spectrum (i)

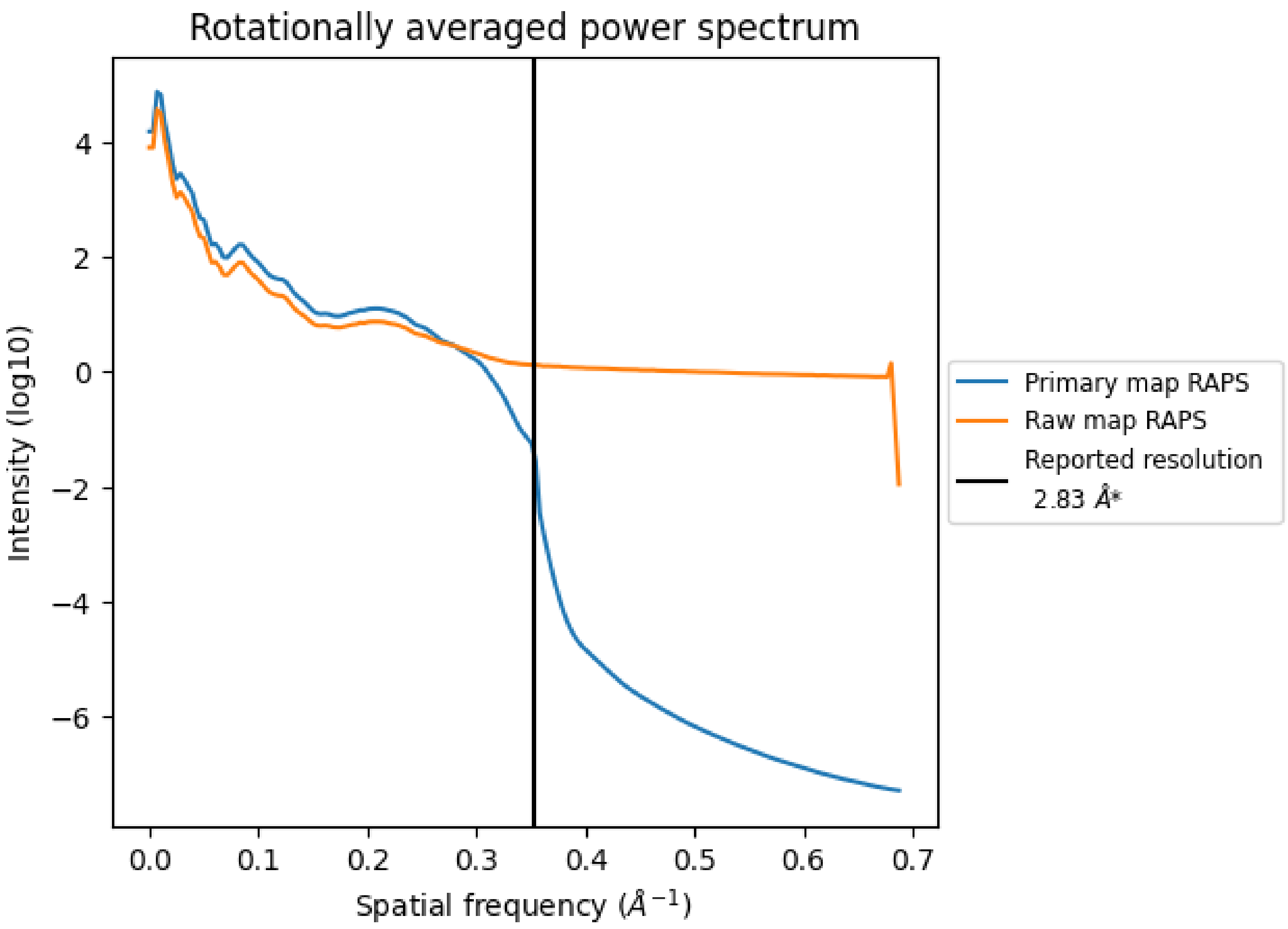


*Reported resolution corresponds to spatial frequency of 0.353 Å$^{-1}$

For Manuscript Review

# 8 Fourier-Shell correlation (i)

Fourier-Shell Correlation (FSC) is the most commonly used method to estimate the resolution of single-particle and subtomogram-averaged maps. The shape of the curve depends on the imposed symmetry, mask and whether or not the two 3D reconstructions used were processed from a common reference. The reported resolution is shown as a black line. A curve is displayed for the half-bit criterion in addition to lines showing the 0.143 gold standard cut-off and 0.5 cut-off.

## 8.1 FSC (i)

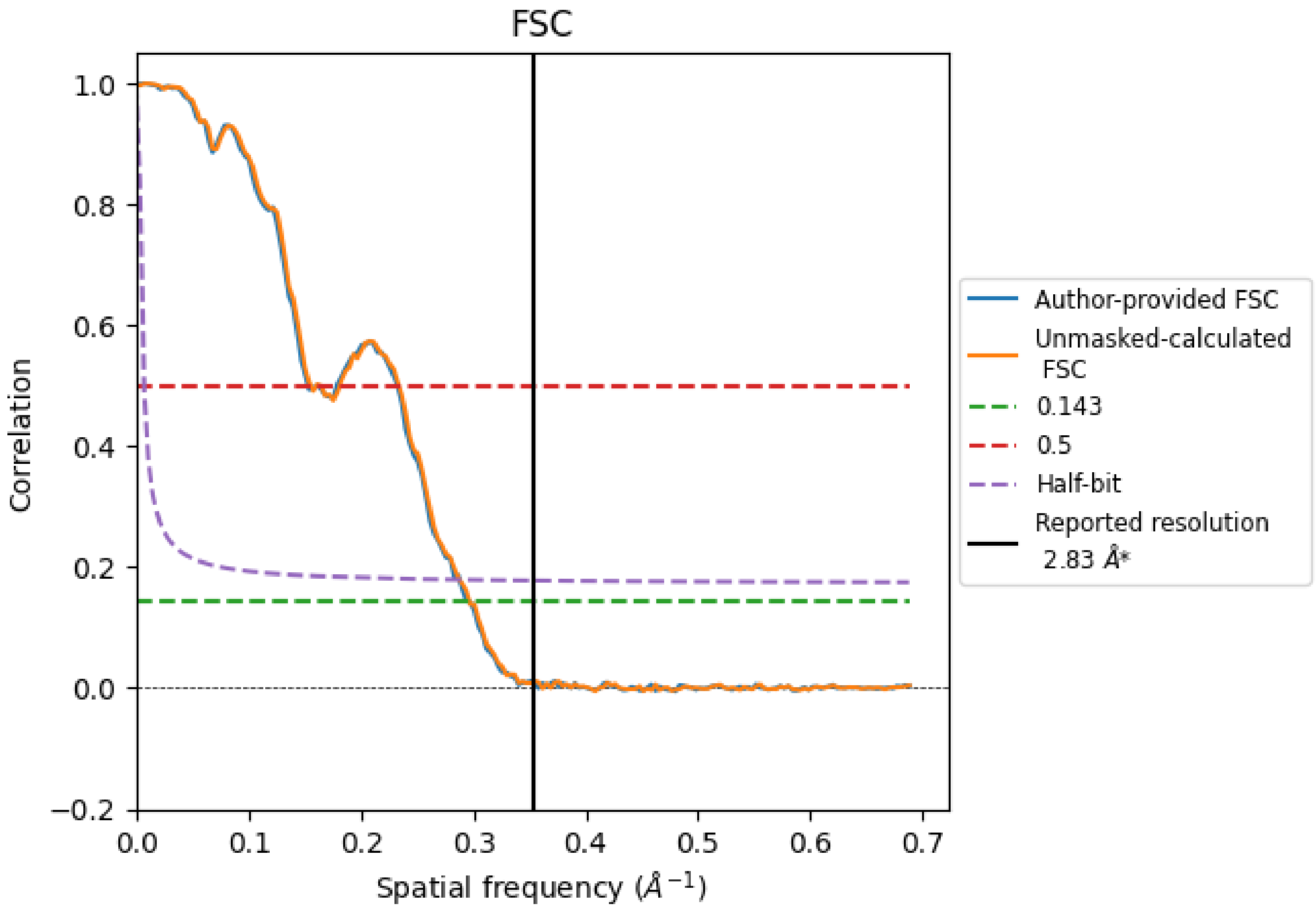


*Reported resolution corresponds to spatial frequency of 0.353 Å$^{-1}$

For Manuscript Review

## 8.2 Resolution estimates (i)

| Resolution estimate (Å) | Estimation criterion (FSC cut-off) | | |
|---|---|---|---|
| | 0.143 | 0.5 | Half-bit |
| Reported by author | 2.83 | - | - |
| Author-provided FSC curve | 3.39 | 6.53 | 3.48 |
| Unmasked-calculated* | 3.37 | 6.44 | 3.48 |

*Resolution estimate based on FSC curve calculated by comparison of deposited half-maps. The value from author-provided FSC intersecting FSC 0.143 CUT-OFF 3.39 differs from the reported value 2.83 by more than 10 %

The value from deposited half-maps intersecting FSC 0.143 CUT-OFF 3.37 differs from the reported value 2.83 by more than 10 %

For Manuscript Review

# 9 Map-model fit (i)

This section contains information regarding the fit between EMDB map EMD-54924 and PDB model 9SI3. Per-residue inclusion information can be found in section 3 on page 6.

## 9.1 Map-model overlay (i)

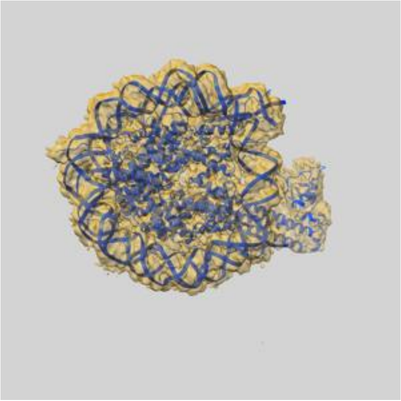

X

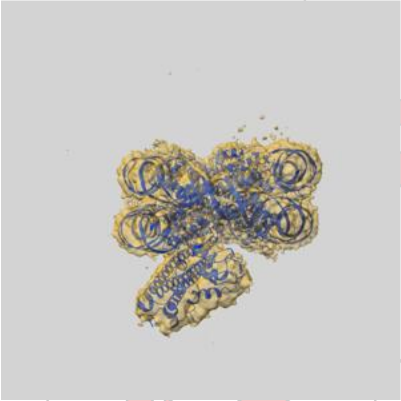

Y

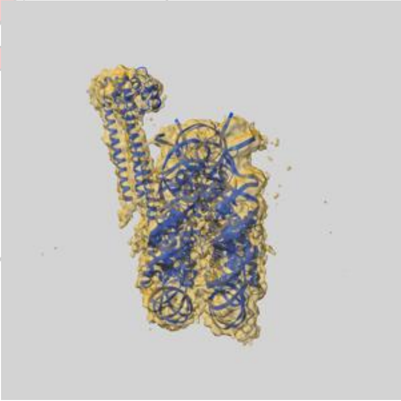

Z

The images above show the 3D surface view of the map at the recommended contour level 0.061 at 50% transparency in yellow overlaid with a ribbon representation of the model coloured in blue. These images allow for the visual assessment of the quality of fit between the atomic model and the map.

For Manuscript Review

## 9.2 Q-score mapped to coordinate model (i)

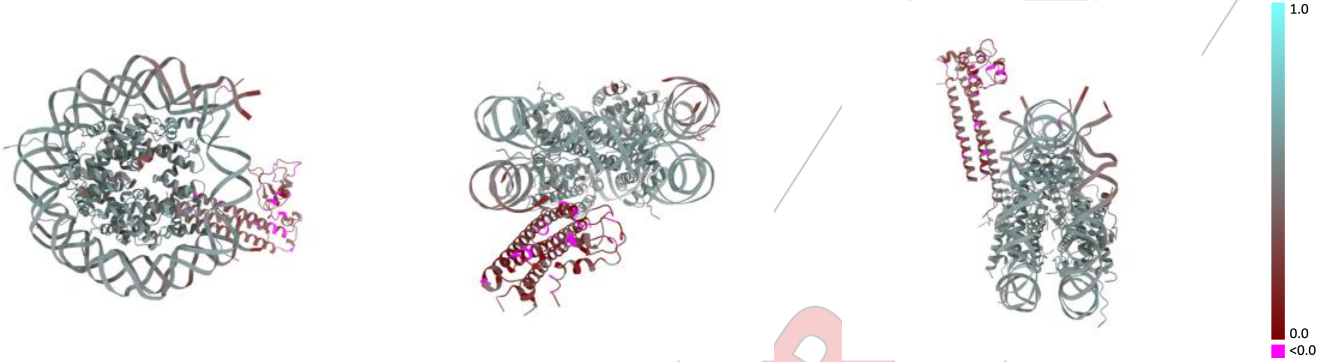


X Y Z

The images above show the model with each residue coloured according its Q-score. This shows their resolvability in the map with higher Q-score values reflecting better resolvability. Please note: Q-score is calculating the resolvability of atoms, and thus high values are only expected at resolutions at which atoms can be resolved. Low Q-score values may therefore be expected for many entries.

## 9.3 Atom inclusion mapped to coordinate model (i)

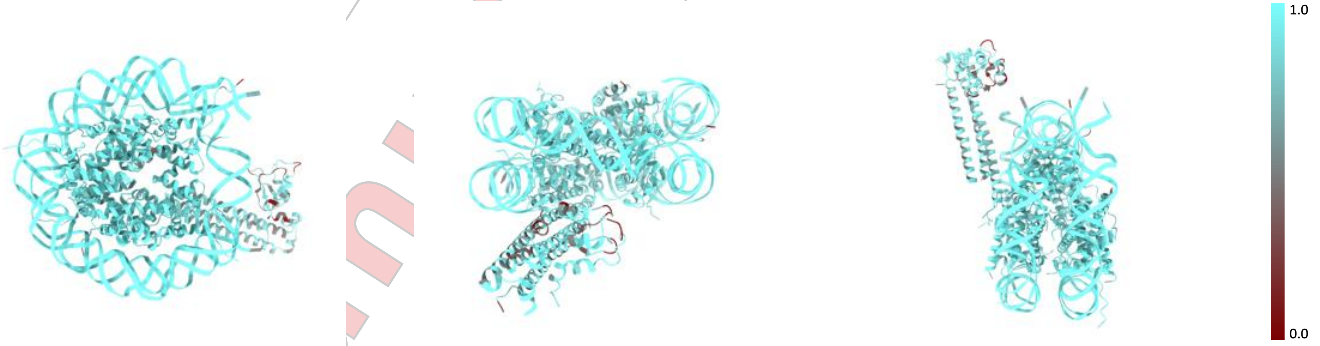


X Y Z

The images above show the model with each residue coloured according to its atom inclusion. This shows to what extent they are inside the map at the recommended contour level (0.061).

For Manuscript Review

## 9.4 Atom inclusion (i)

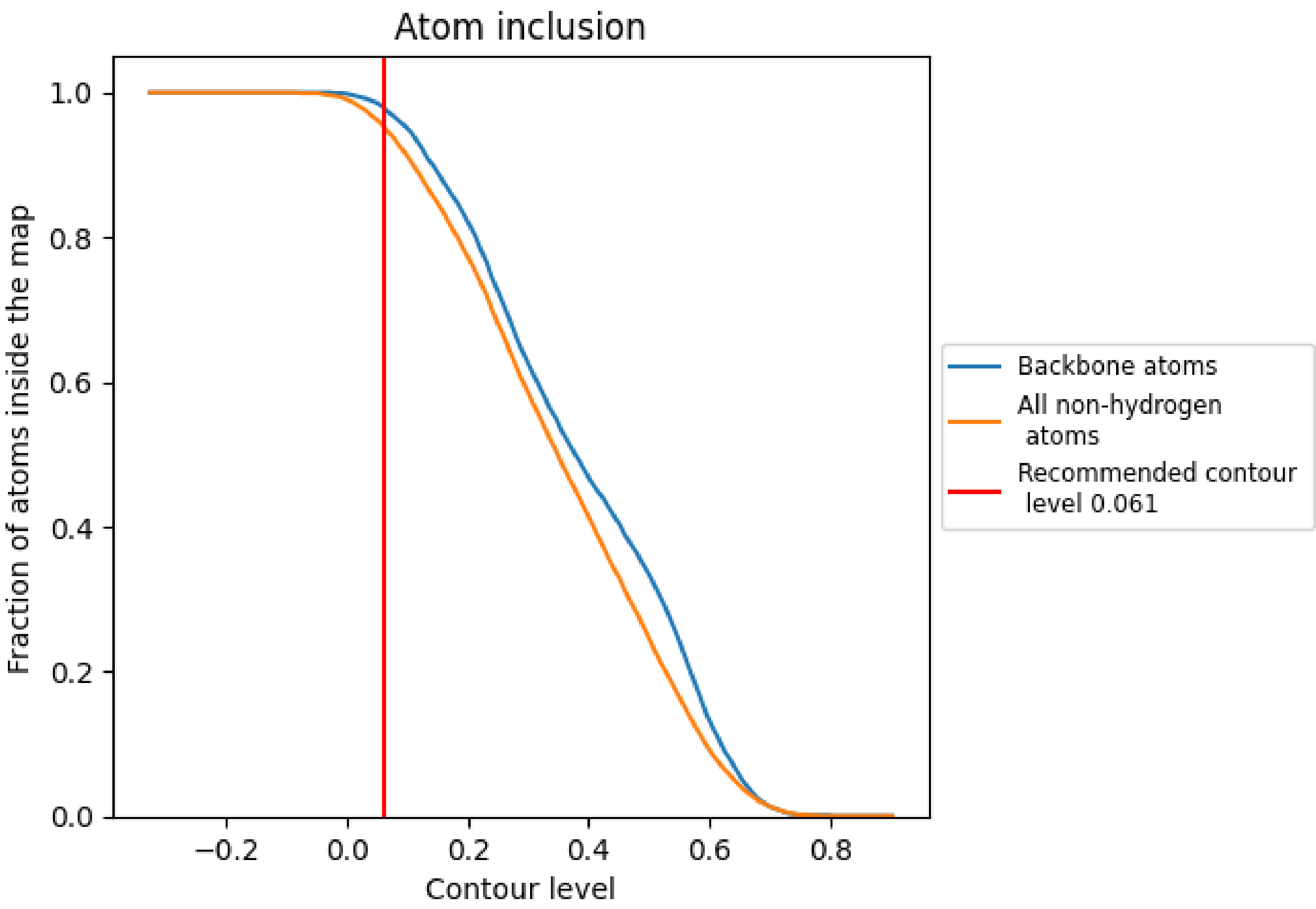


At the recommended contour level, 98% of all backbone atoms, 95% of all non-hydrogen atoms, are inside the map.

For Manuscript Review

## 9.5 Map-model fit summary (i)

The table lists the average atom inclusion at the recommended contour level (0.061) and Q-score for the entire model and for each chain.

| Chain | Atom inclusion | Q-score |
|---|---|---|
| All | 0.9530 | 0.4760 |
| A | 0.9720 | 0.5480 |
| B | 0.9810 | 0.5540 |
| C | 0.9690 | 0.5400 |
| D | 0.9740 | 0.5260 |
| E | 0.9650 | 0.5380 |
| F | 0.9730 | 0.5470 |
| G | 0.9570 | 0.5370 |
| H | 0.9710 | 0.5320 |
| I | 0.9950 | 0.5120 |
| J | 0.9960 | 0.5100 |
| L | 0.7450 | 0.3490 |
| M | 0.7510 | 0.1470 |
| N | 0.7930 | 0.2130 |
| O | 0.8490 | 0.2740 |

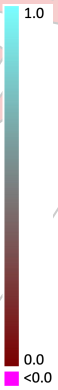


For Manuscript Review
