# Supplementary figures and images for: Nucleosome interaction of the CPC secures centromeric chromatin integrity and chromosome segregation fidelity
Source: EMBO J. 2025 Oct 27;44(22):6556–97. doi: 10.1038/s44318-025-00594-y (PMC12624148; doi:10.1038/s44318-025-00594-y)

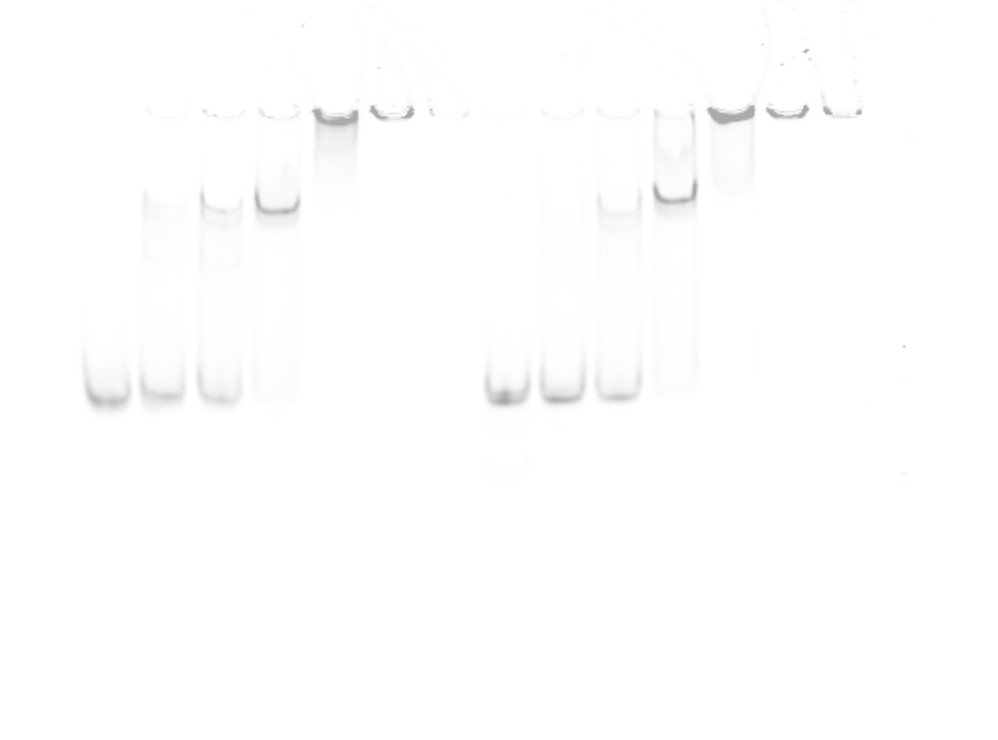

Supplement: Supplementary file 5 — Source data Fig. 3 [file 44318_2025_594_MOESM5_ESM.zip › Figure 3/Fig3A/Fig3A.EMSA_AcidicPatch_220425_Image_000336_220425_EMSA2.tif]

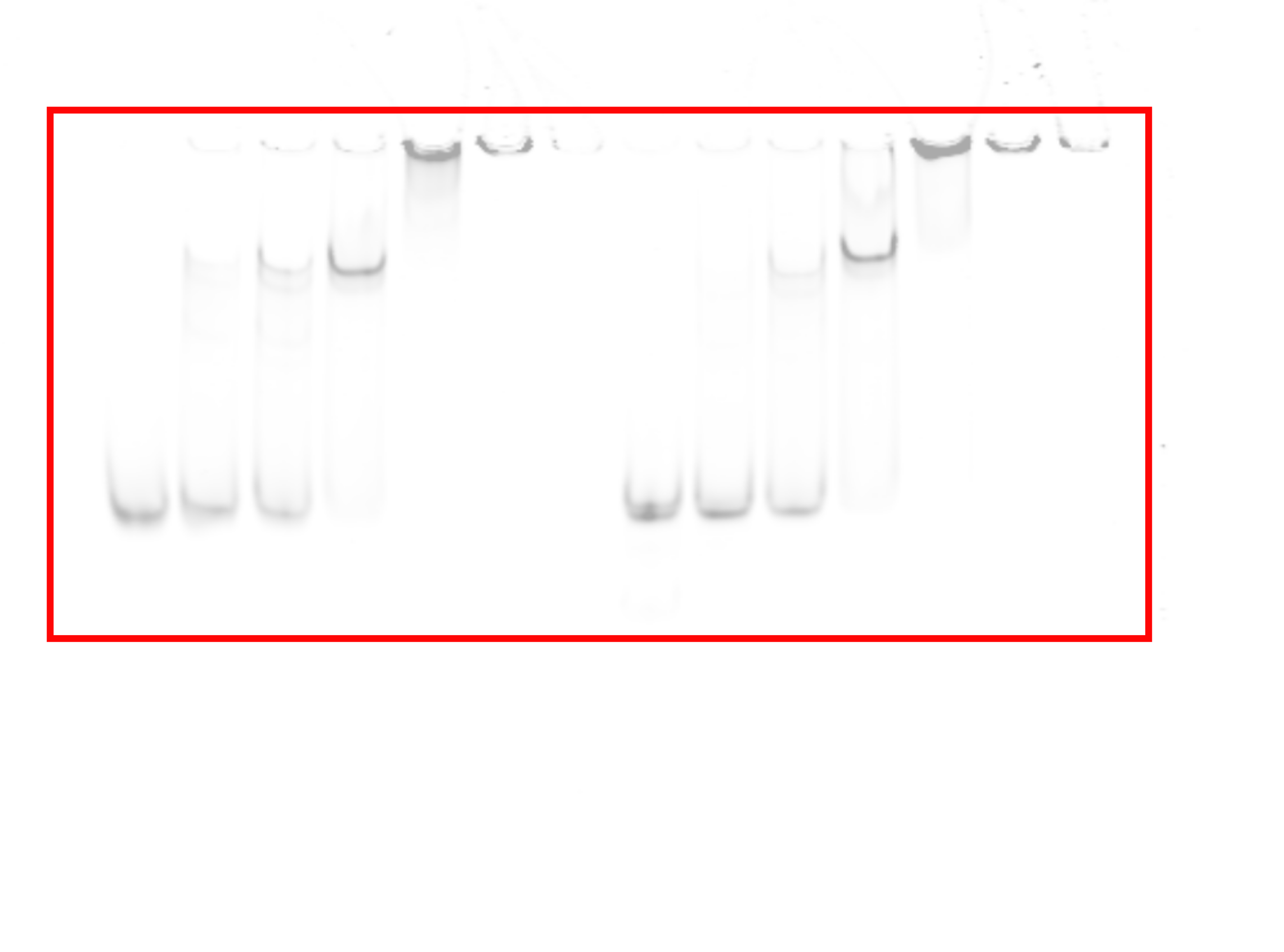

Supplement: Supplementary file 5 — Source data Fig. 3 [file 44318_2025_594_MOESM5_ESM.zip › Figure 3/Fig3A/Fig3A.EMSA_AcidicPatch_220425_Image_000336_220425_EMSA2_CROPPED.tif]

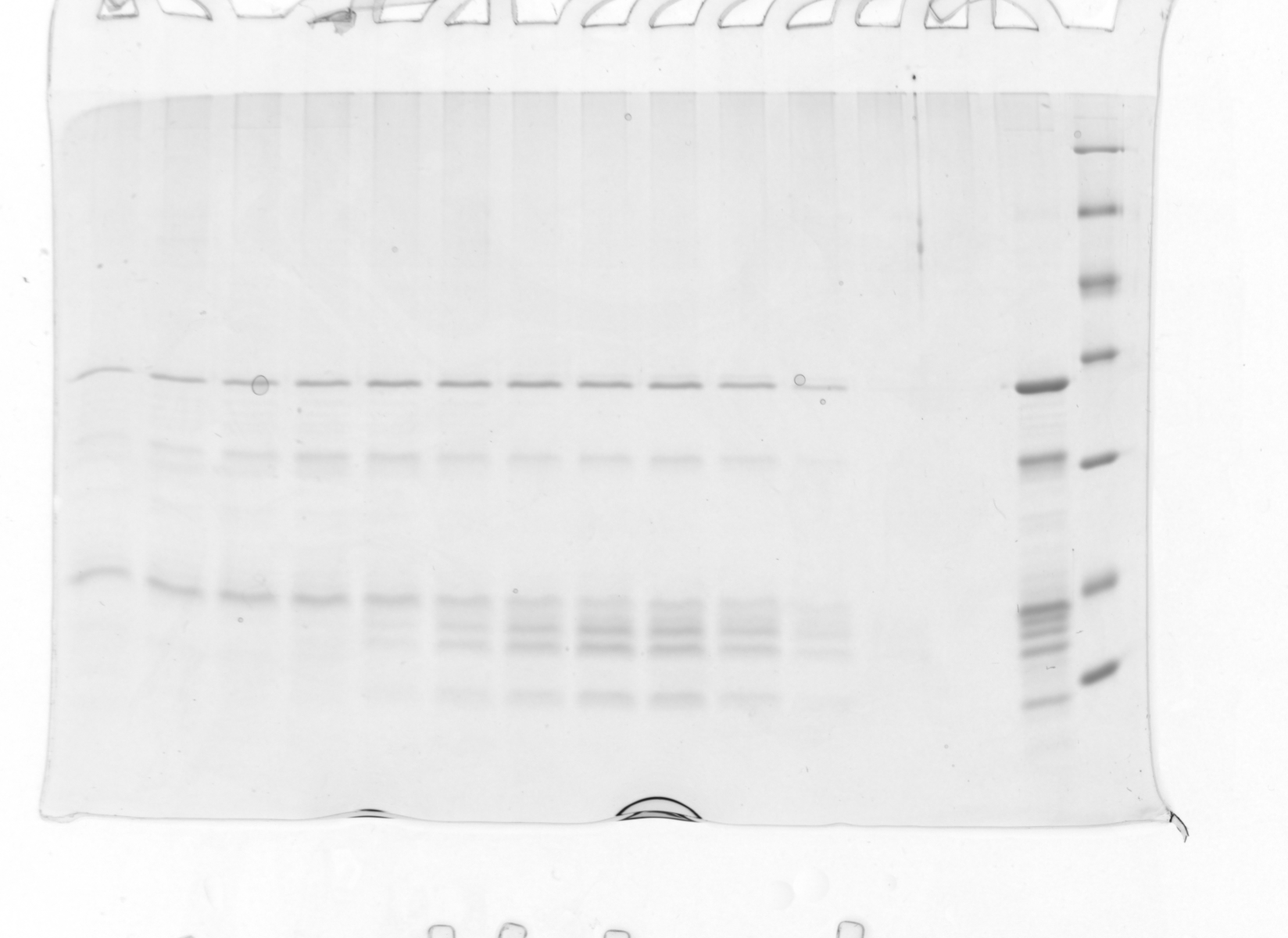

Supplement: Supplementary file 5 — Source data Fig. 3 [file 44318_2025_594_MOESM5_ESM.zip › Figure 3/Fig3B/Fig3B_CPC1_190_DN_NCP.tif]

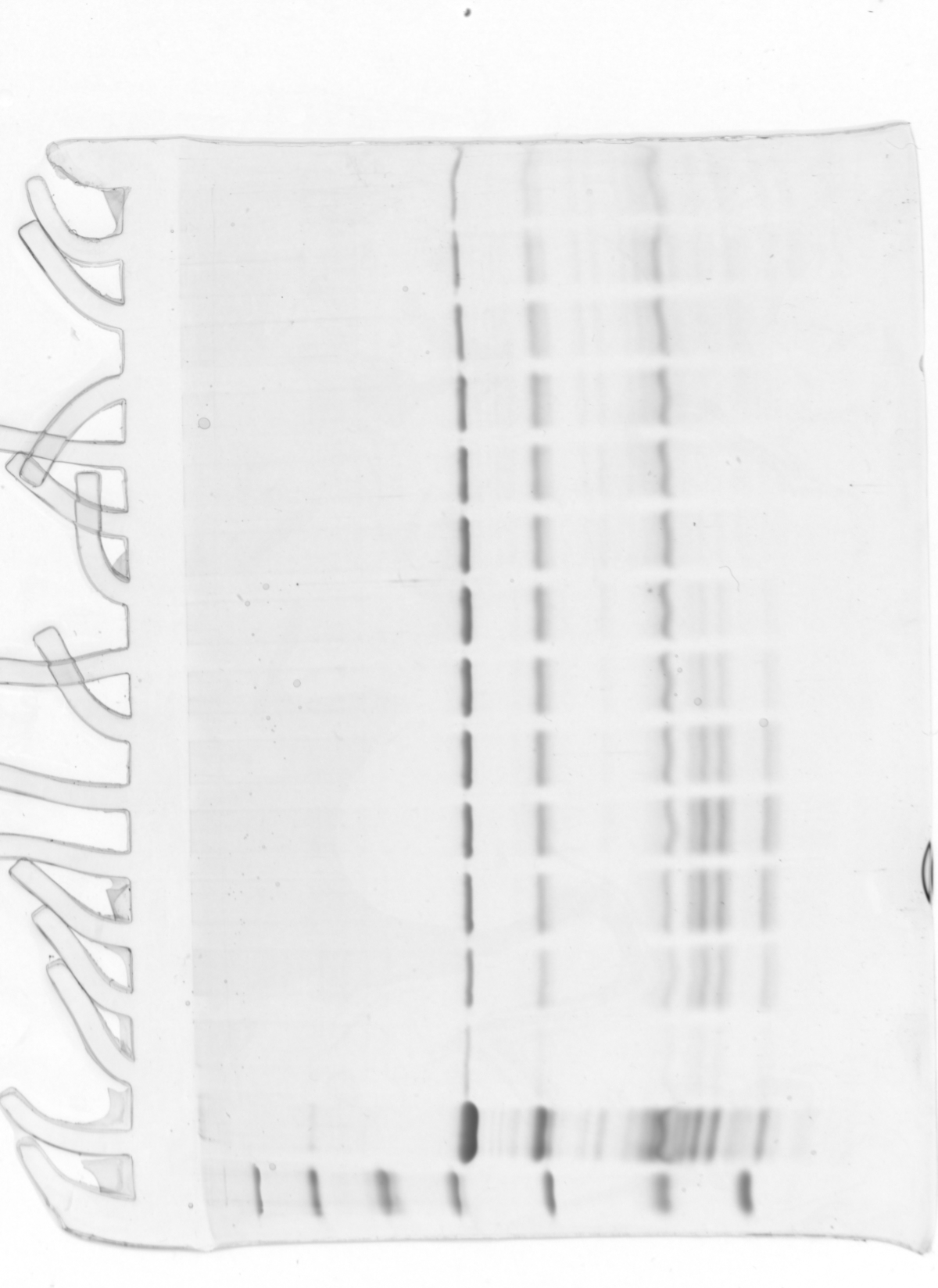

Supplement: Supplementary file 5 — Source data Fig. 3 [file 44318_2025_594_MOESM5_ESM.zip › Figure 3/Fig3B/Fig3B_CPC1_190_NCP.tif]

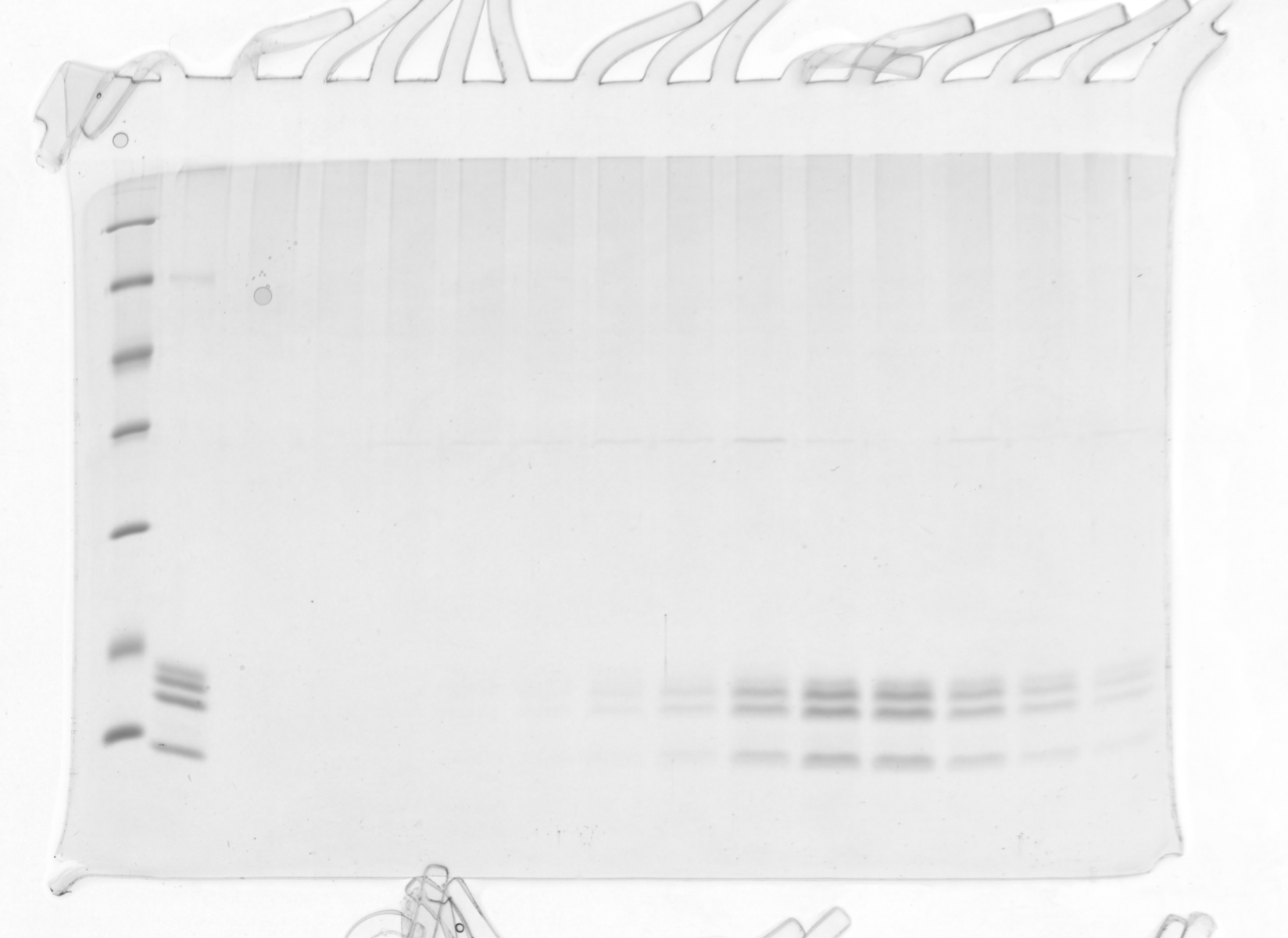

Supplement: Supplementary file 5 — Source data Fig. 3 [file 44318_2025_594_MOESM5_ESM.zip › Figure 3/Fig3B/Fig3B_NCP.tif]

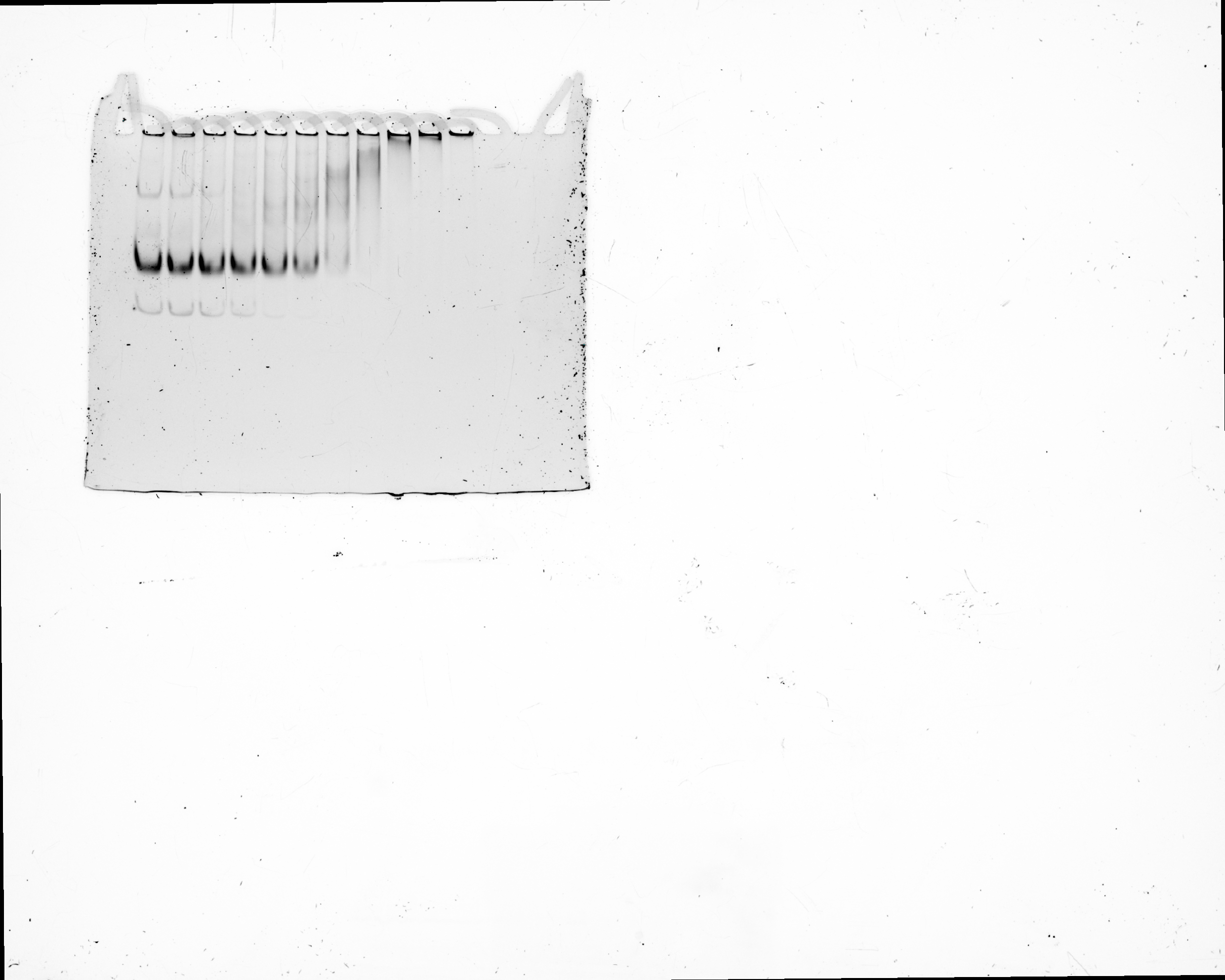

Supplement: Supplementary file 5 — Source data Fig. 3 [file 44318_2025_594_MOESM5_ESM.zip › Figure 3/Fig3C/CPC_1-1903A_w_180825_EMSA4_JP lab 2025-08-18 12h37m40s(SYBR® Safe)_adj.tif]

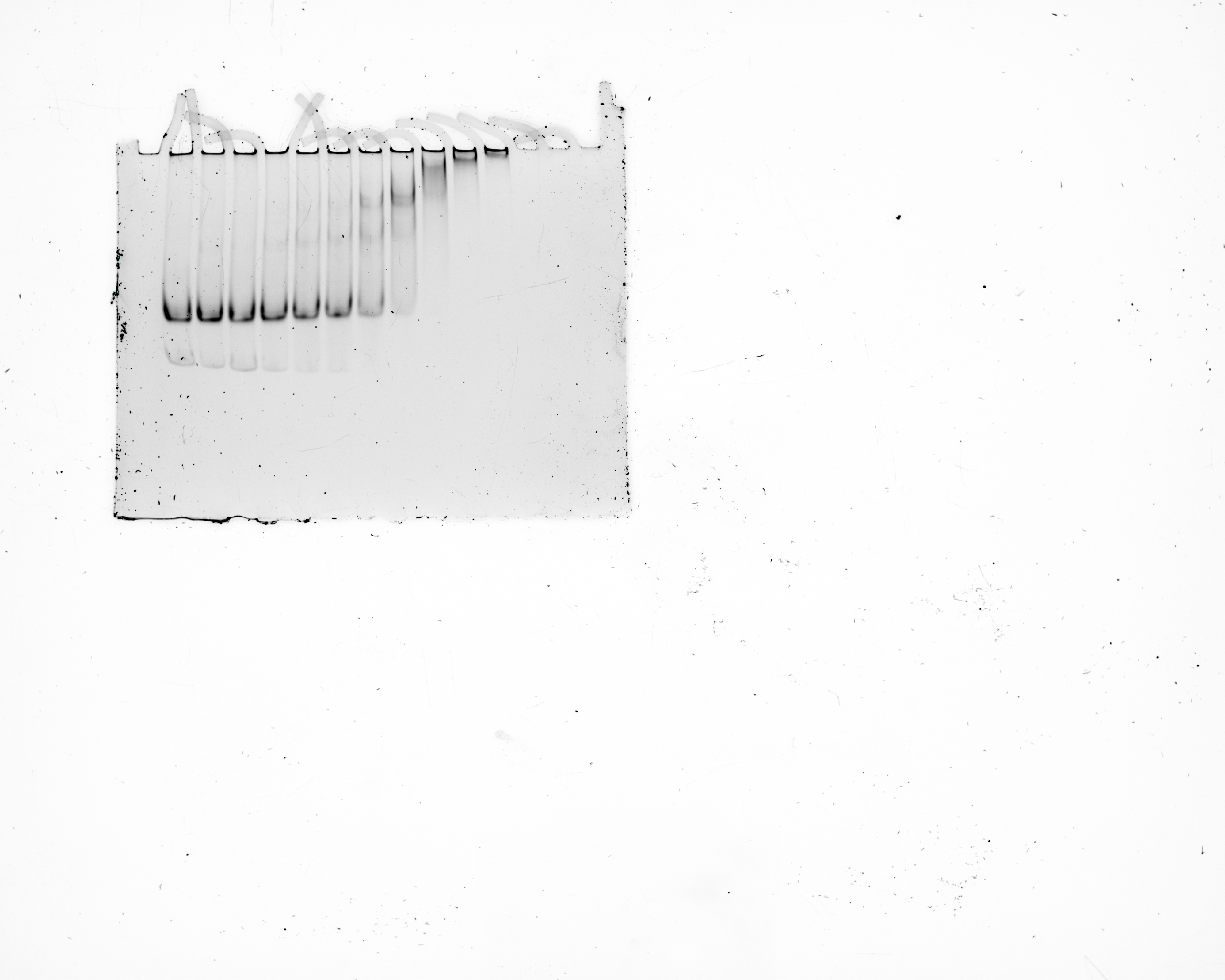

Supplement: Supplementary file 5 — Source data Fig. 3 [file 44318_2025_594_MOESM5_ESM.zip › Figure 3/Fig3C/CPC_1-190_11K12A3A_a-sat250825_EMSA4_JP lab 2025-08-25 11h51m59s(SYBR® Safe).tif]

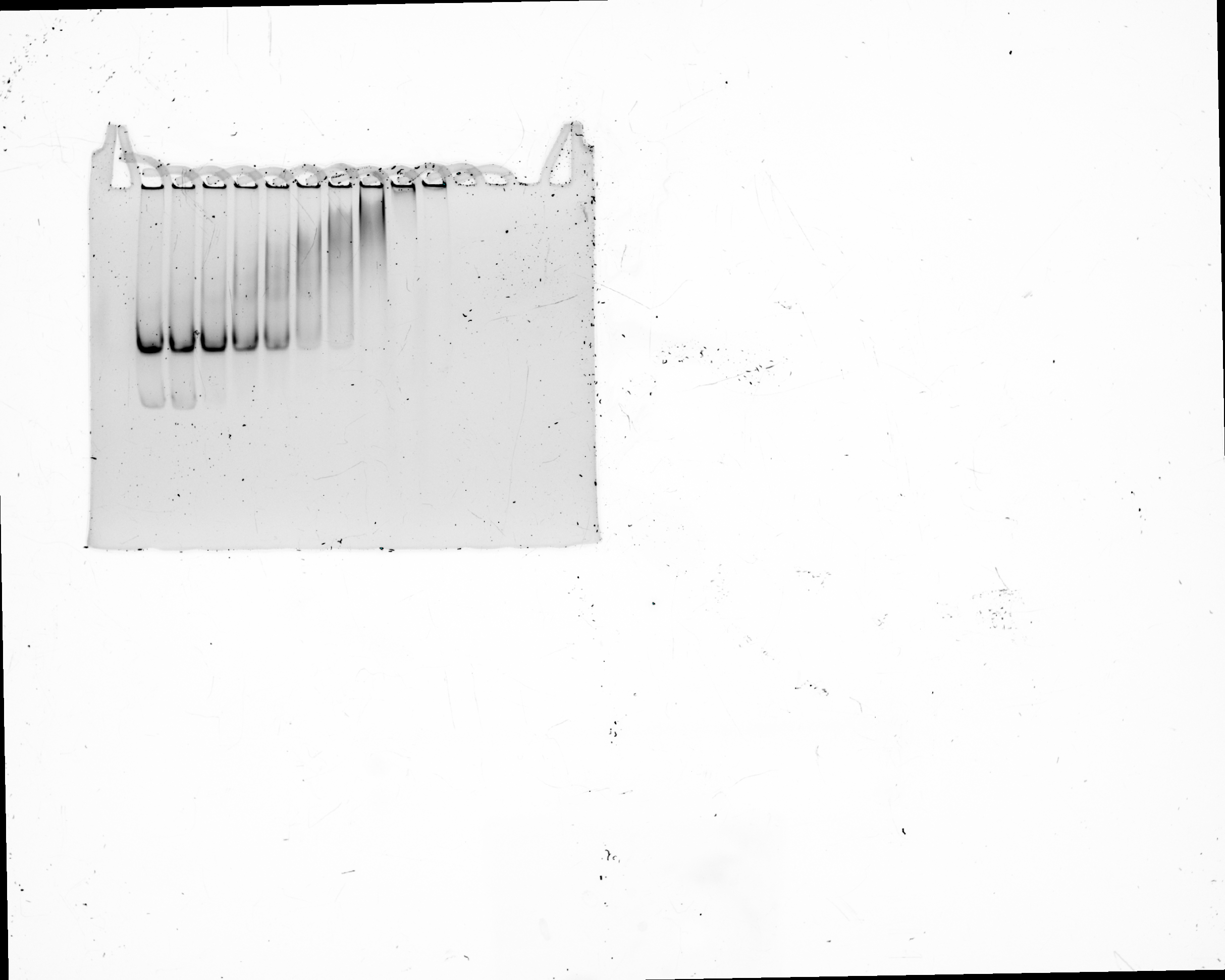

Supplement: Supplementary file 5 — Source data Fig. 3 [file 44318_2025_594_MOESM5_ESM.zip › Figure 3/Fig3C/CPC_1-190_11_end_a-sat_190825_EMSA2_JP lab 2025-08-19 12h05m27s(SYBR® Safe)_adj.tif]

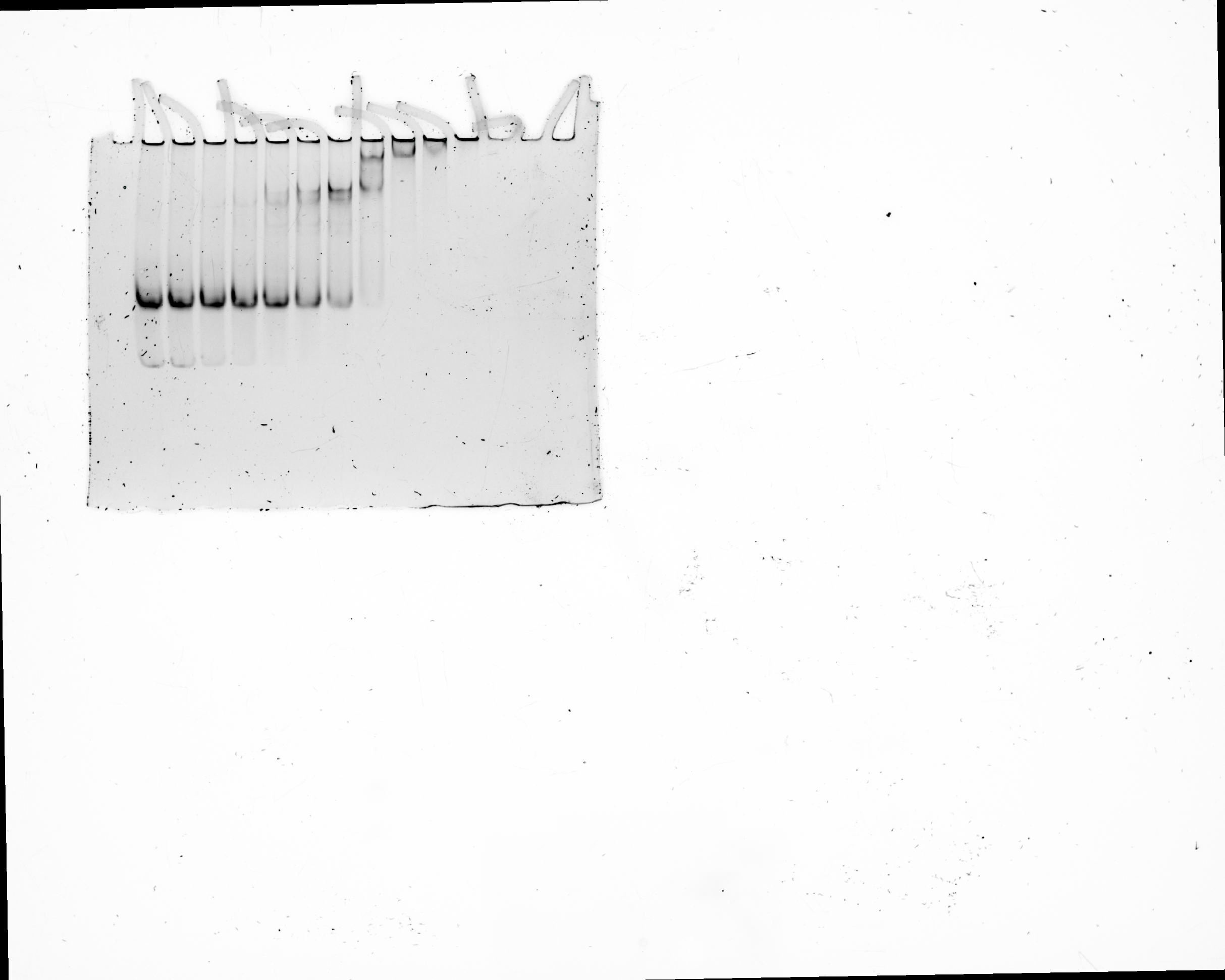

Supplement: Supplementary file 5 — Source data Fig. 3 [file 44318_2025_594_MOESM5_ESM.zip › Figure 3/Fig3C/CPC_1-190_11_K12A_a-sat250825_EMSA2_JP lab 2025-08-25 11h49m05s(SYBR® Safe)_adj.tif]

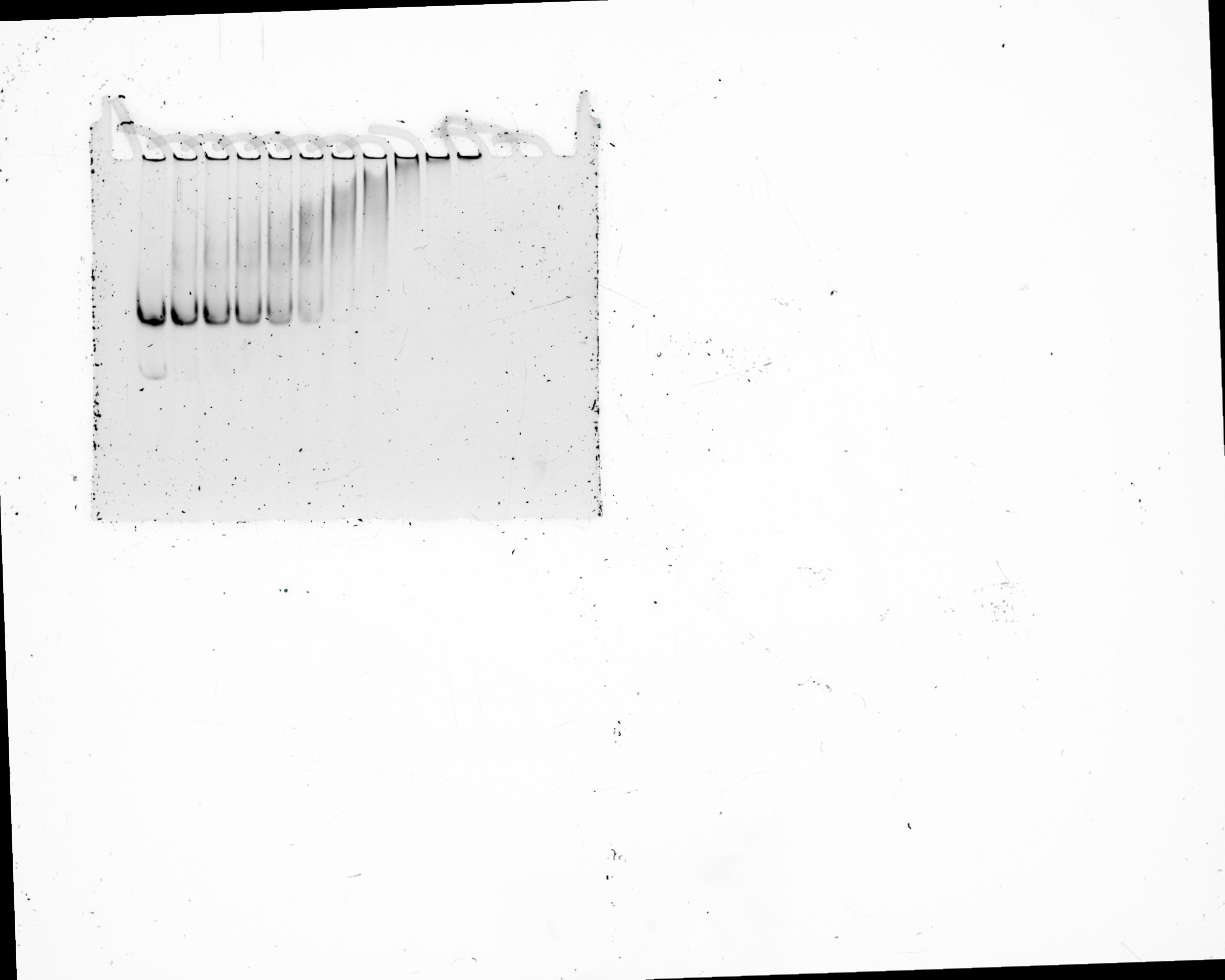

Supplement: Supplementary file 5 — Source data Fig. 3 [file 44318_2025_594_MOESM5_ESM.zip › Figure 3/Fig3C/CPC_1-190_3A_a_sat_190825_EMSA4_JP lab 2025-08-19 12h08m06s(SYBR® Safe)_adj.tif]

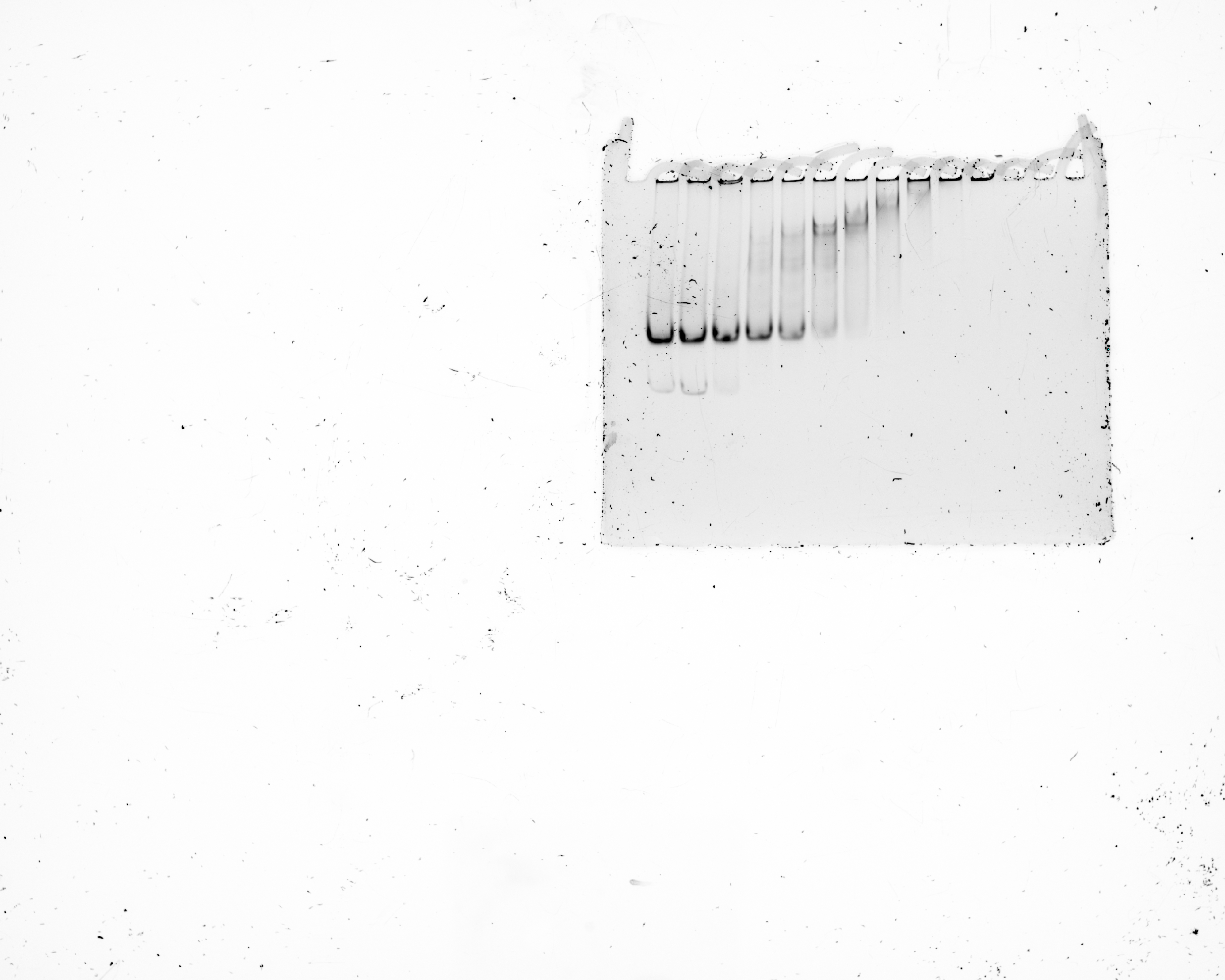

Supplement: Supplementary file 5 — Source data Fig. 3 [file 44318_2025_594_MOESM5_ESM.zip › Figure 3/Fig3C/CPC_1-190_a-sat_200825_EMSA3_JP lab 2025-08-20 11h17m12s(SYBR® Safe)_adj.tif]

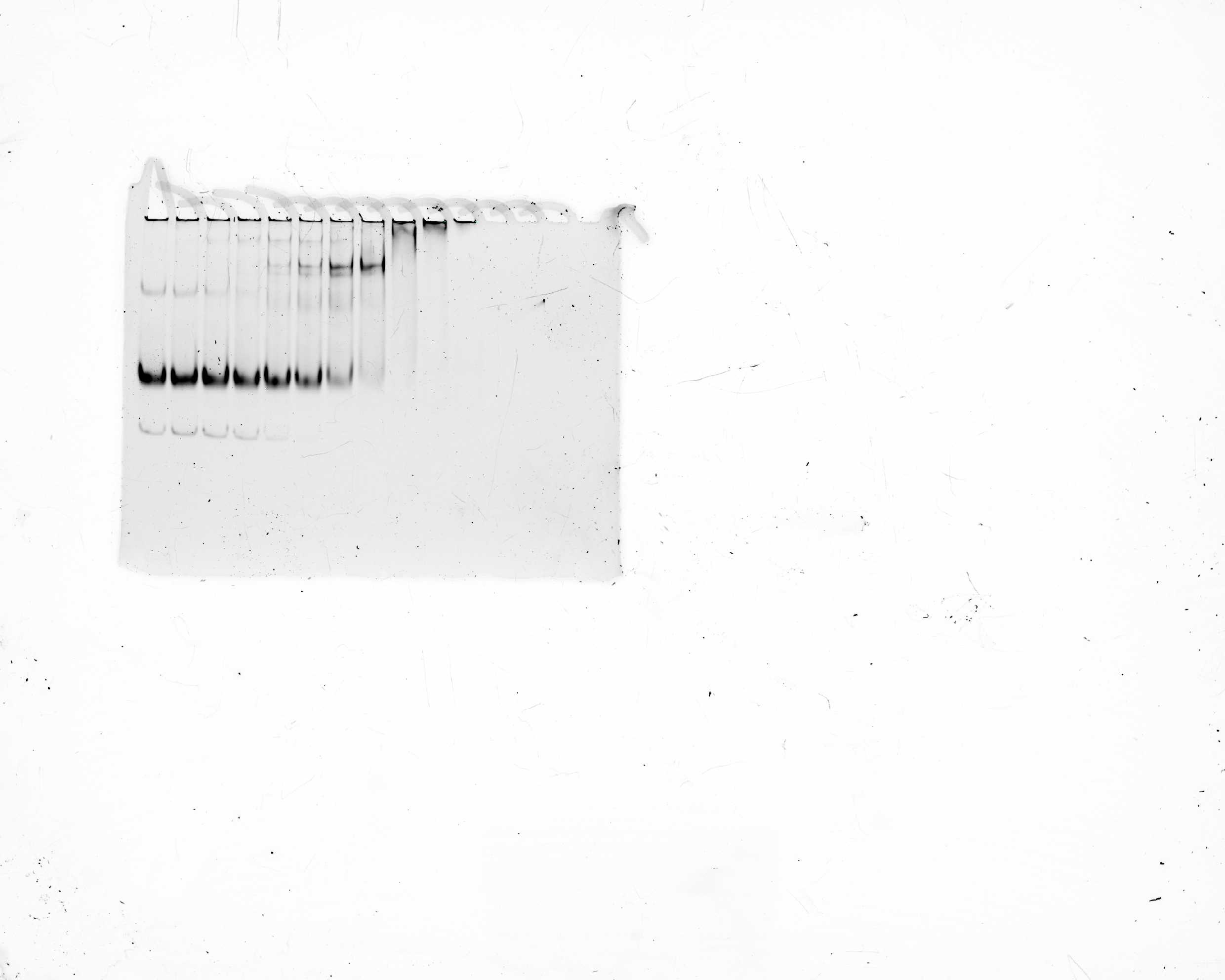

Supplement: Supplementary file 5 — Source data Fig. 3 [file 44318_2025_594_MOESM5_ESM.zip › Figure 3/Fig3C/CPC_1-190_DNK12A_3A_w_140825_EMSA4_JP lab 2025-08-14 12h28m08s(SYBR® Safe)_adj.tif]

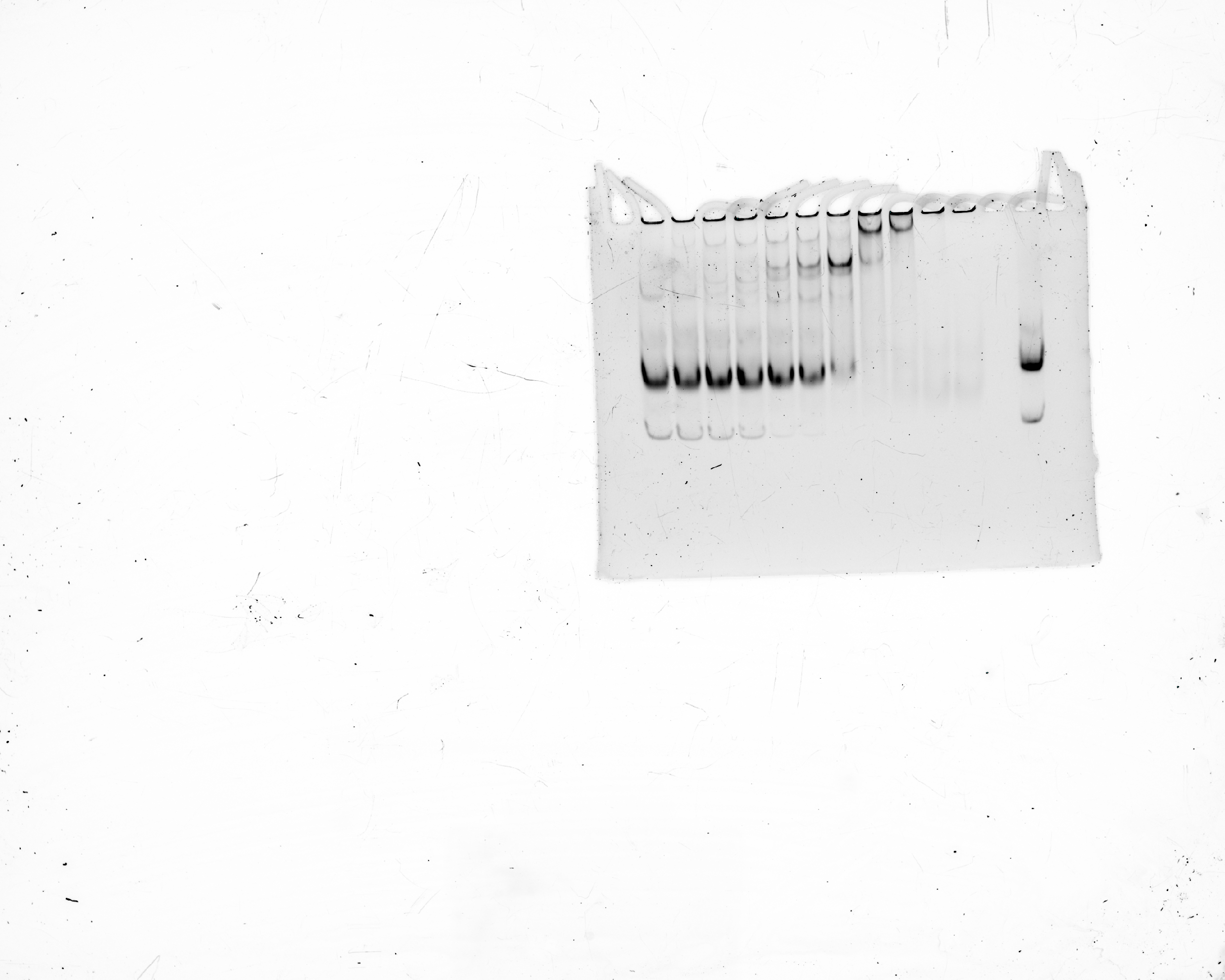

Supplement: Supplementary file 5 — Source data Fig. 3 [file 44318_2025_594_MOESM5_ESM.zip › Figure 3/Fig3C/CPC_1-190_DNK12A_w_140825_EMSA1_JP lab 2025-08-14 12h24m20s(SYBR® Safe)_adj.tif]

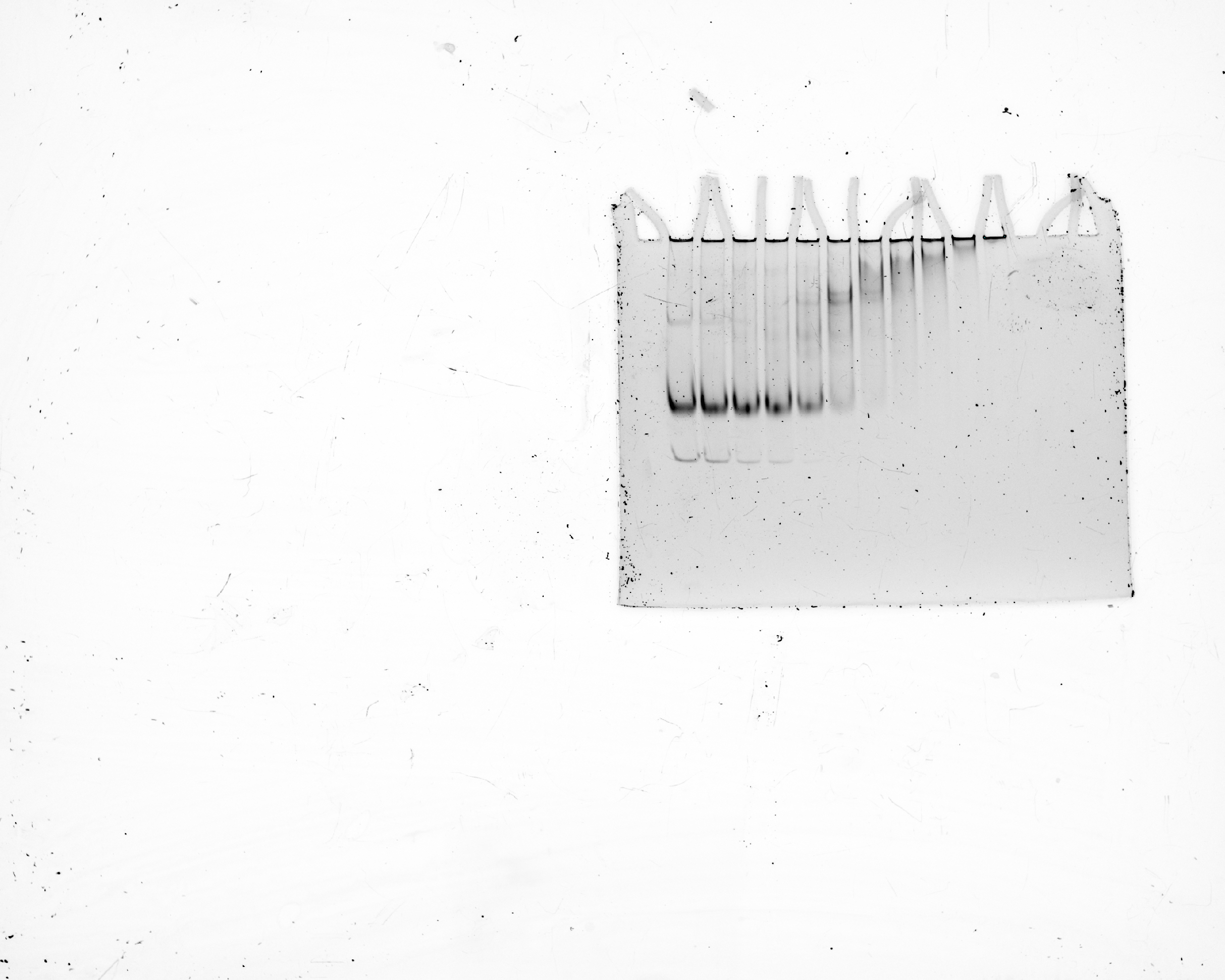

Supplement: Supplementary file 5 — Source data Fig. 3 [file 44318_2025_594_MOESM5_ESM.zip › Figure 3/Fig3C/CPC_1-190_DN_w_180825_EMSA7_JP lab 2025-08-18 16h07m51s(SYBR® Safe)_adj.tif]

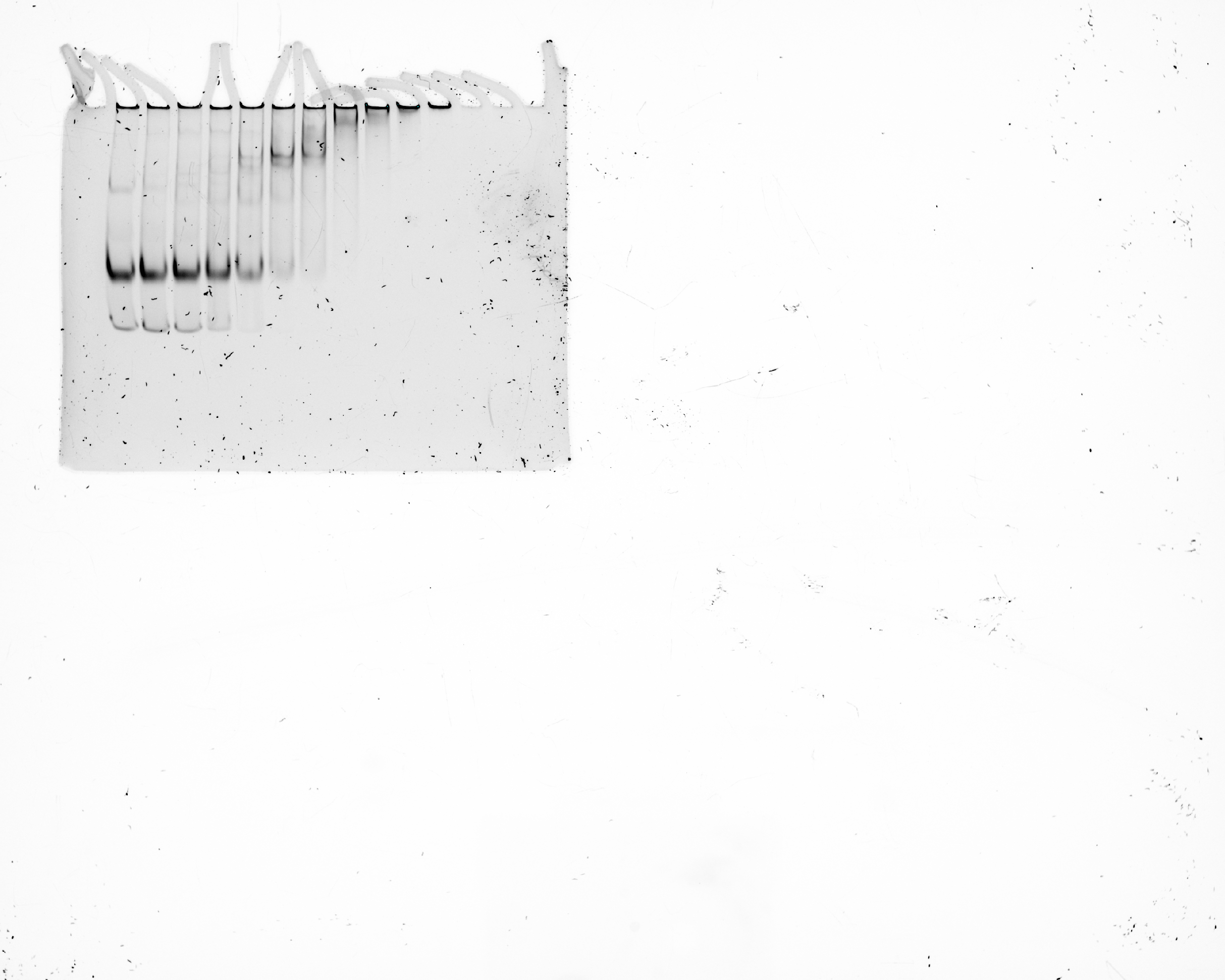

Supplement: Supplementary file 5 — Source data Fig. 3 [file 44318_2025_594_MOESM5_ESM.zip › Figure 3/Fig3C/CPC_1-190_w_260825_EMSA1_JP lab 2025-08-26 12h48m11s(SYBR® Safe)_adj.tif]

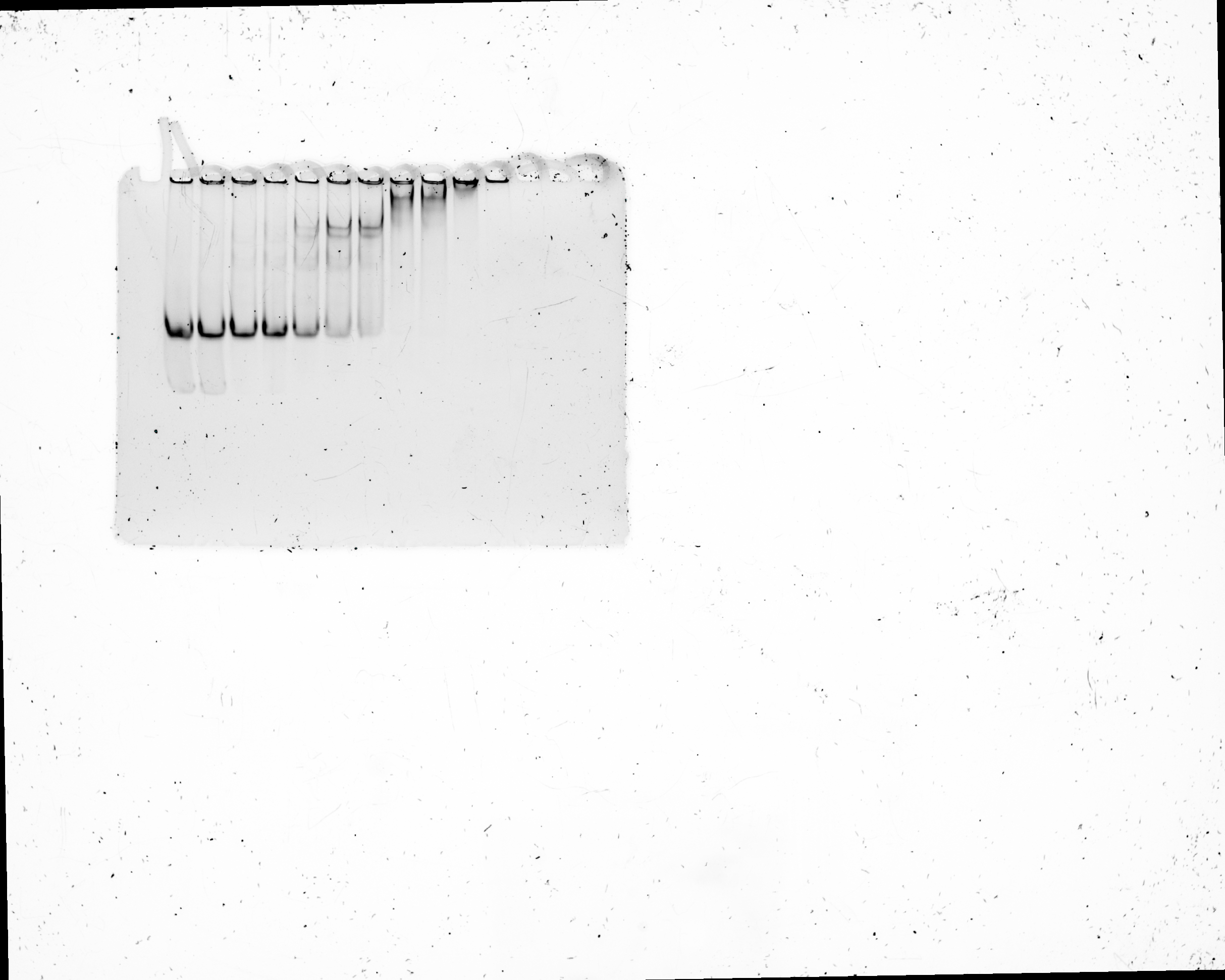

Supplement: Supplementary file 6 — Source data Fig. 4 [file 44318_2025_594_MOESM6_ESM.zip › Figure 4/Fig4C/CPC_1-190_6A_a_sat_200825_EMSA8_JP lab 2025-08-20 14h53m30s(SYBR® Safe)_adj.tif]

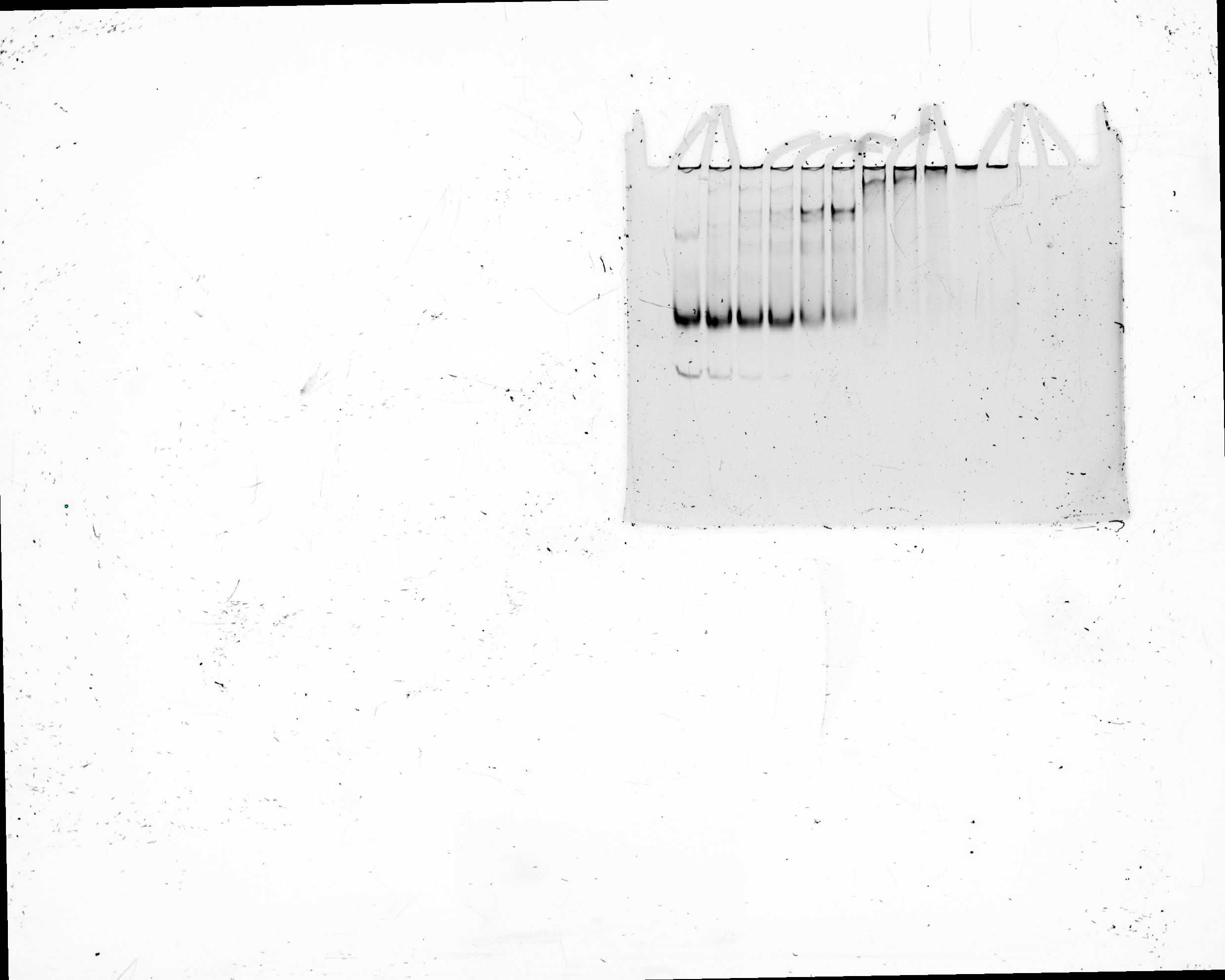

Supplement: Supplementary file 6 — Source data Fig. 4 [file 44318_2025_594_MOESM6_ESM.zip › Figure 4/Fig4C/CPC_1-190_6A_w_150825_EMSA8_JP lab 2025-08-15 15h09m13s(SYBR® Safe)_adj.tif]

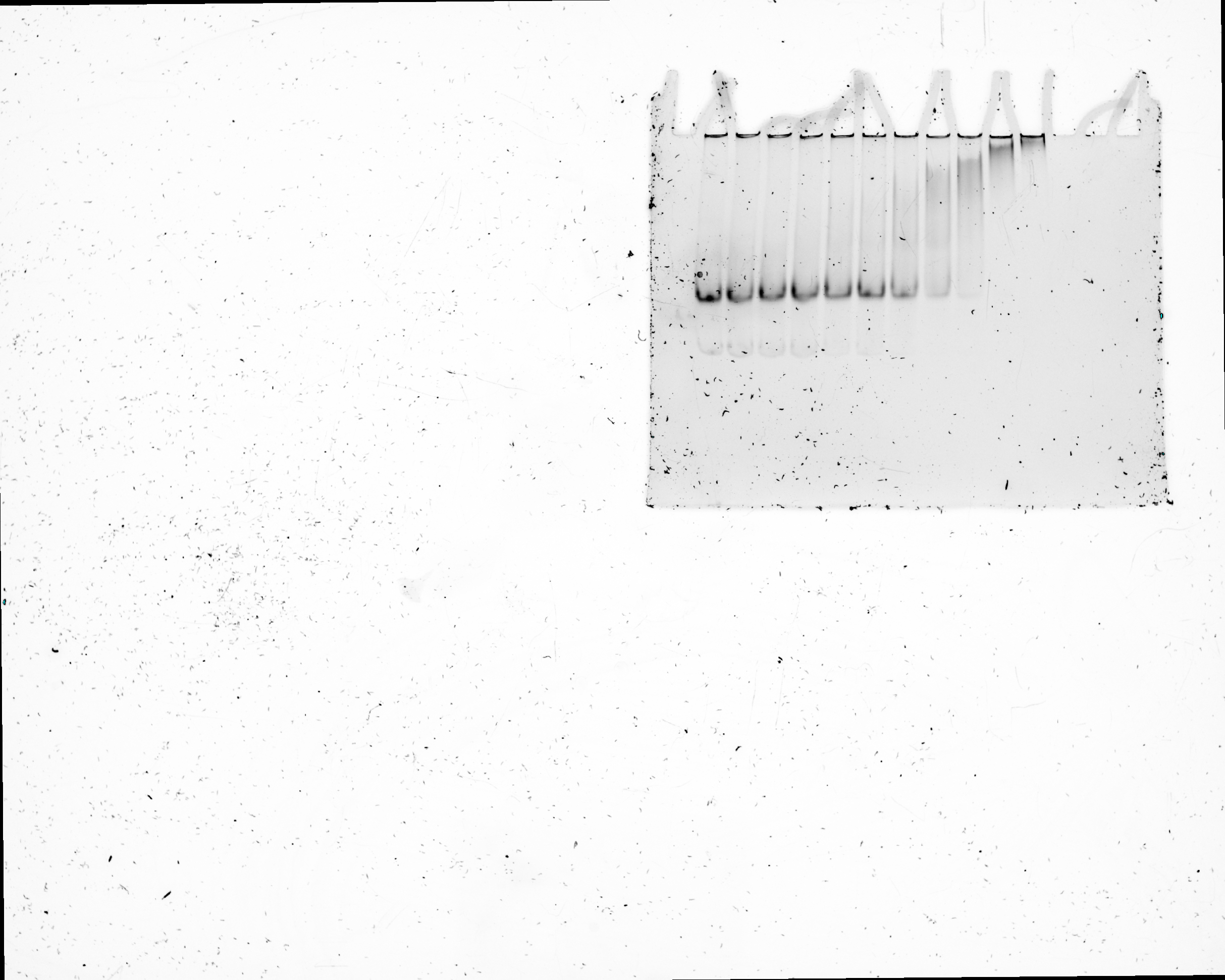

Supplement: Supplementary file 6 — Source data Fig. 4 [file 44318_2025_594_MOESM6_ESM.zip › Figure 4/Fig4C/CPC_1-190_Dloop6A_a-sat_220825_EMSA3_JP lab 2025-08-22 11h55m01s(SYBR® Safe)_adj.tif]

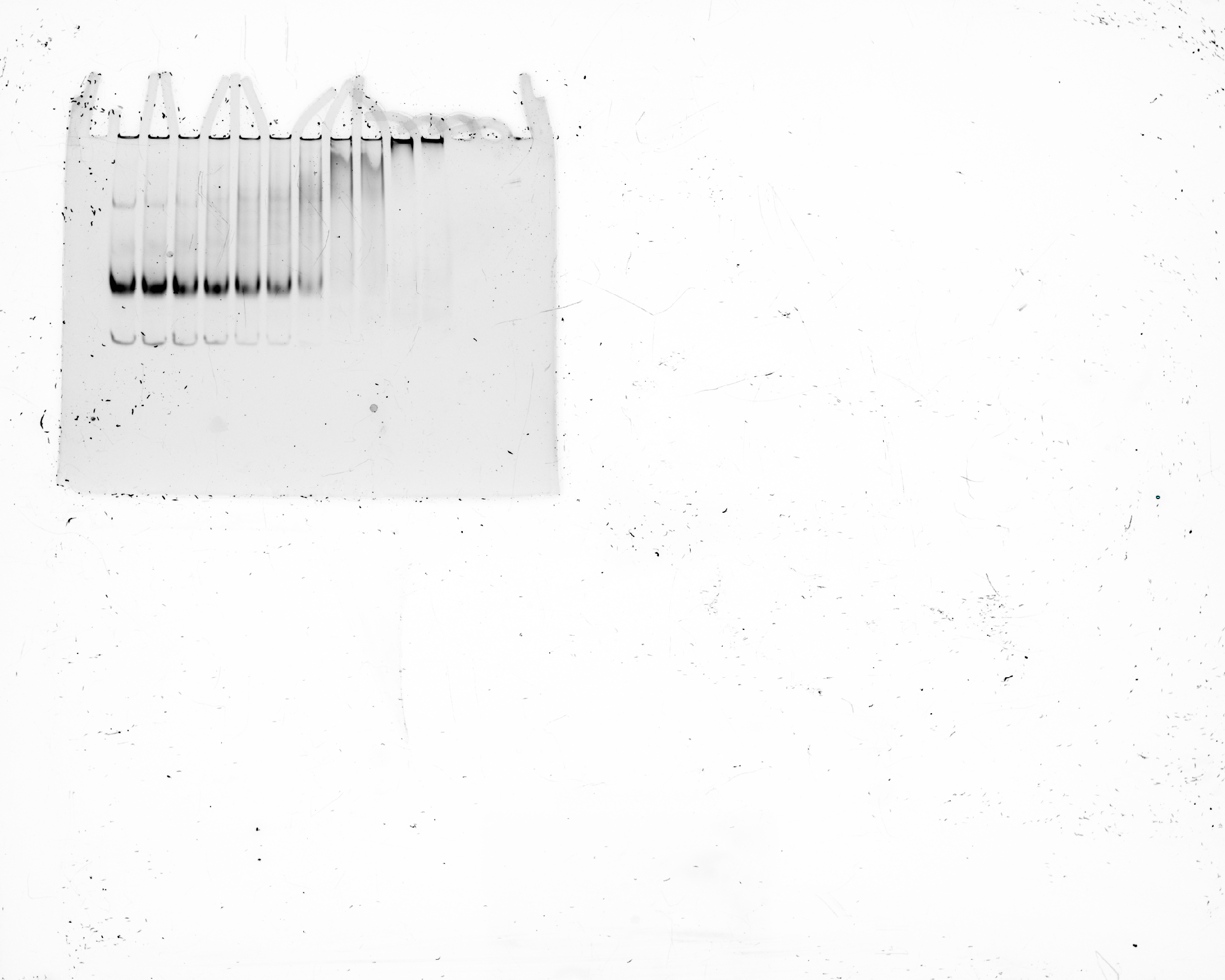

Supplement: Supplementary file 6 — Source data Fig. 4 [file 44318_2025_594_MOESM6_ESM.zip › Figure 4/Fig4C/CPC_1-190_Dloop6A_w_150825_EMSA6_JP lab 2025-08-15 15h06m34s(SYBR® Safe)_adj.tif]

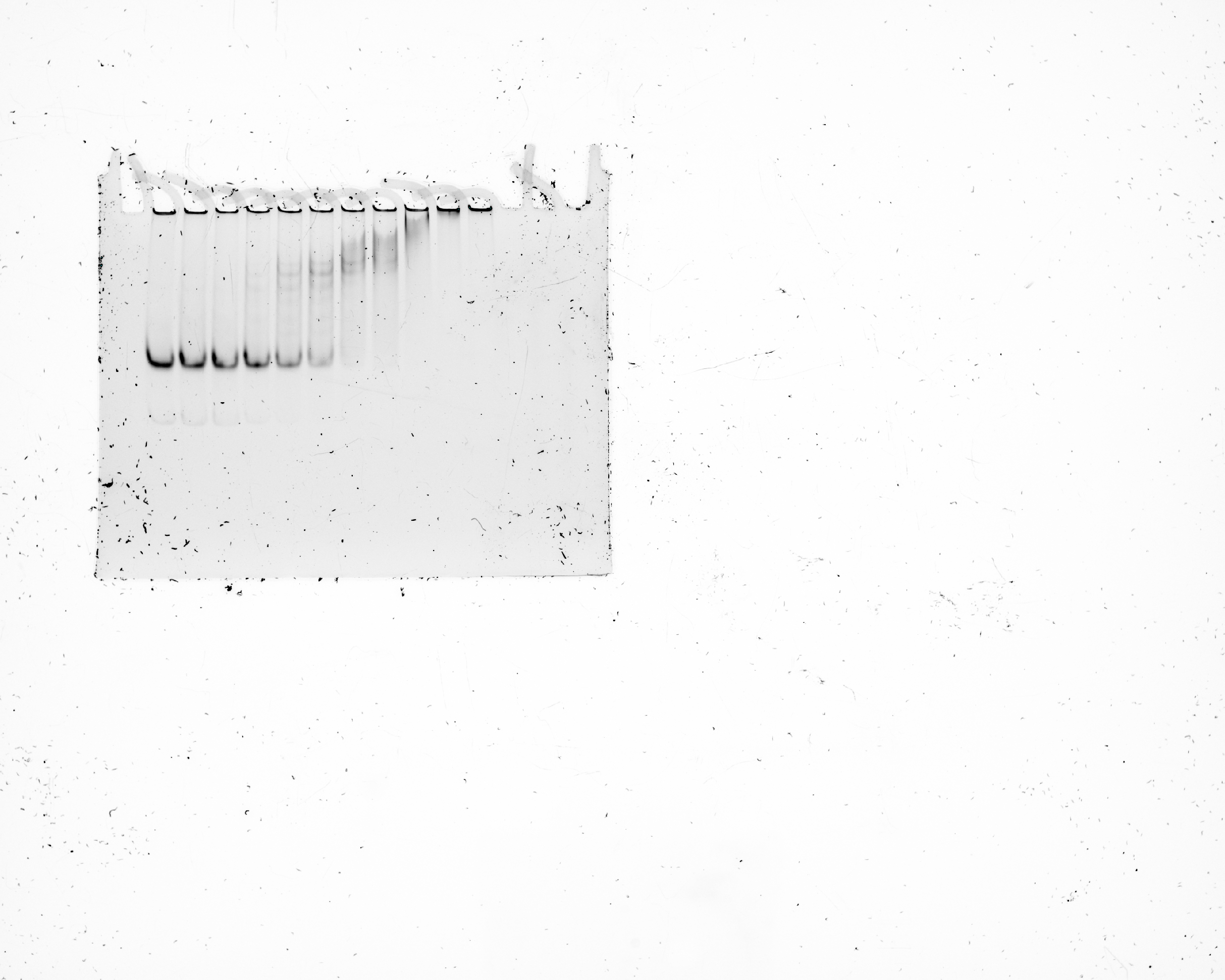

Supplement: Supplementary file 6 — Source data Fig. 4 [file 44318_2025_594_MOESM6_ESM.zip › Figure 4/Fig4C/CPC_1-190_Dloop_a-sat_210825_EMSA3_JP lab 2025-08-21 11h54m47s(SYBR® Safe)_adj.tif]

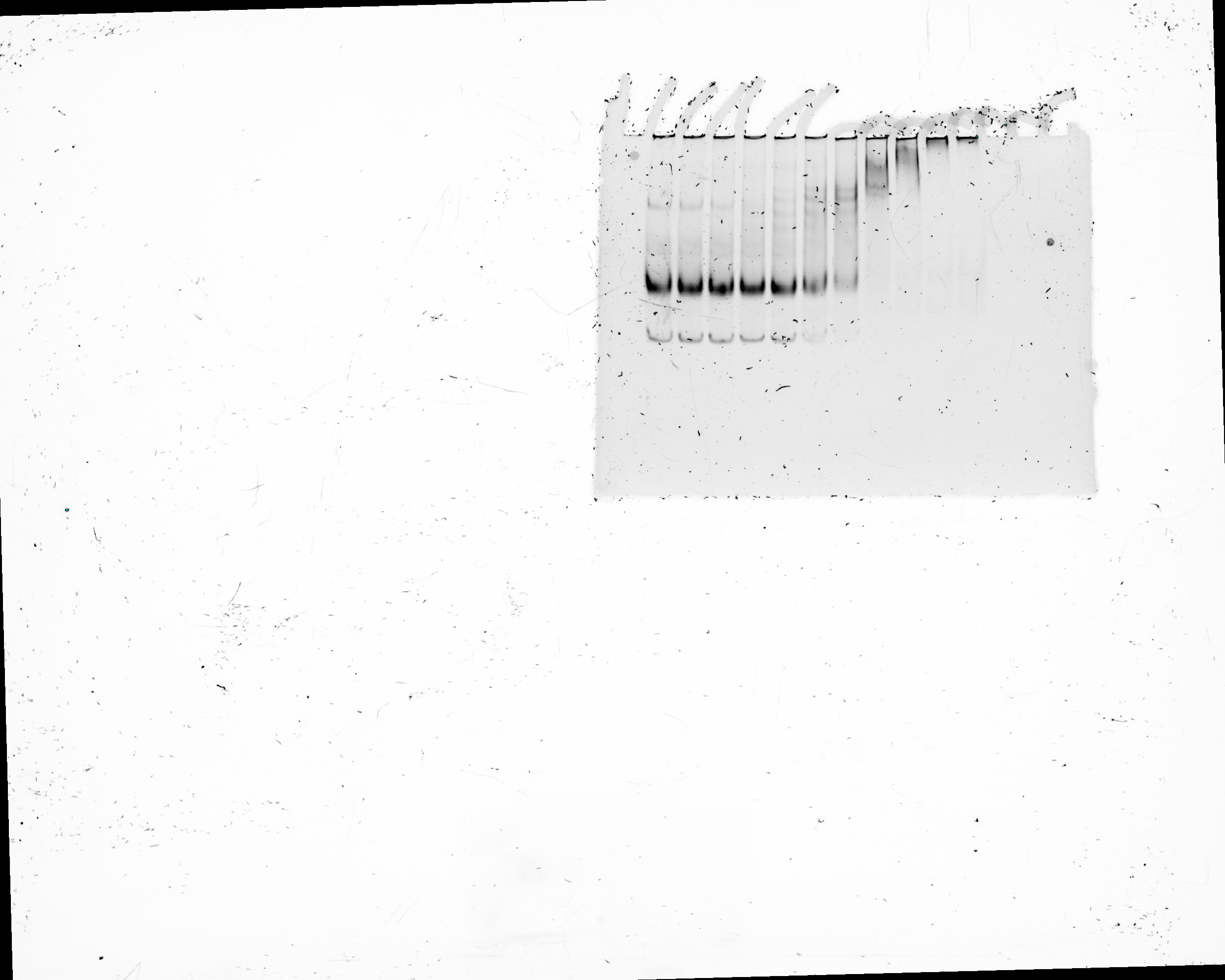

Supplement: Supplementary file 6 — Source data Fig. 4 [file 44318_2025_594_MOESM6_ESM.zip › Figure 4/Fig4C/CPC_1-190_Dloop_w_150825_EMSA5_JP lab 2025-08-15 15h03m27s(SYBR® Safe)_adj.tif]

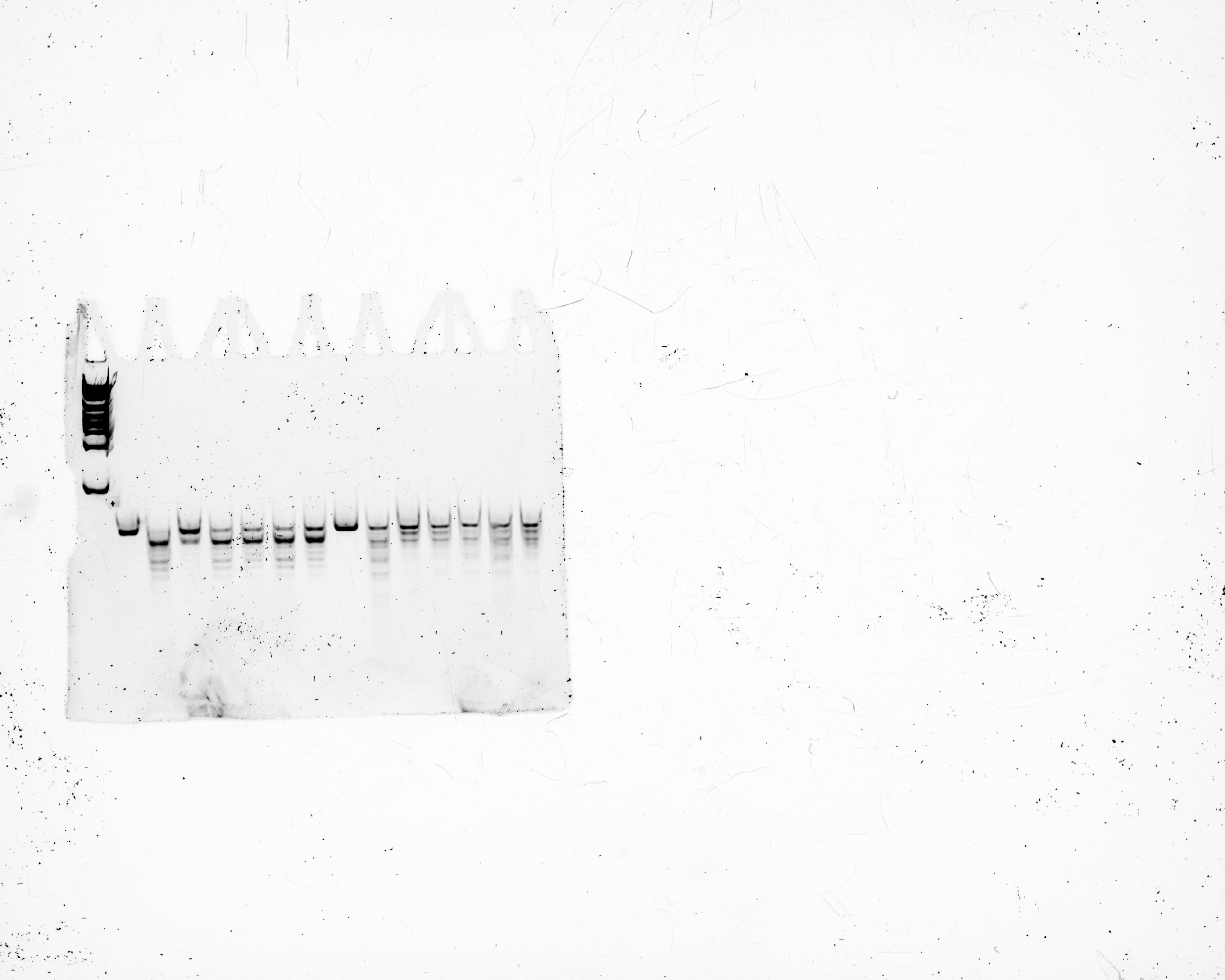

Supplement: Supplementary file 7 — Source data Fig. 5 [file 44318_2025_594_MOESM7_ESM.zip › Figure 5/Figure_5E/Fig5E_JP lab 2025-05-13 21h32m46s(SYBR┬« Safe).tif]

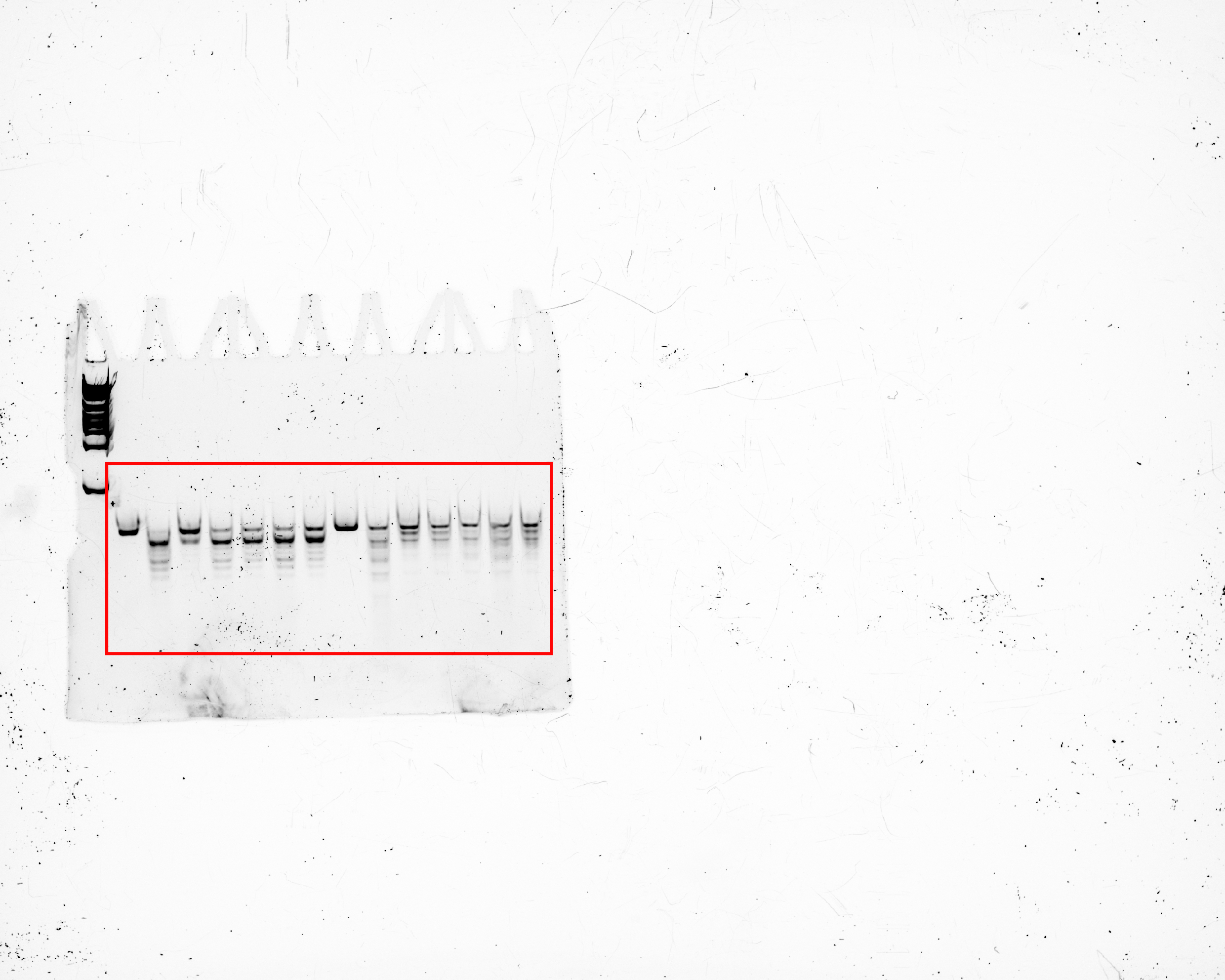

Supplement: Supplementary file 7 — Source data Fig. 5 [file 44318_2025_594_MOESM7_ESM.zip › Figure 5/Figure_5E/Fig5E_MonoMNase_0073CC1B-EC45-4242-B8CB-909A2C24F1D6_cropped_.tiff]

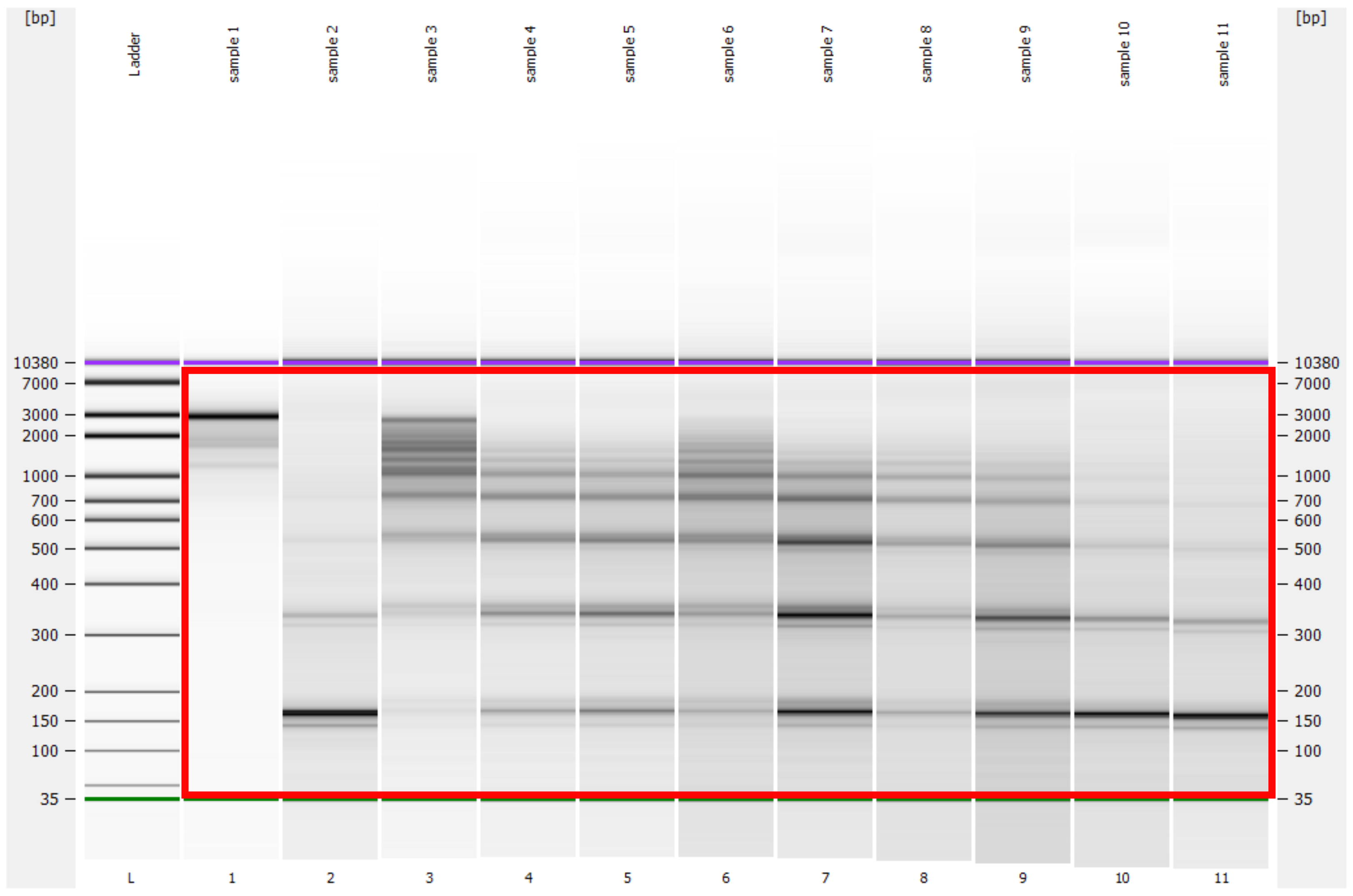

Supplement: Supplementary file 7 — Source data Fig. 5 [file 44318_2025_594_MOESM7_ESM.zip › Figure 5/Figure_5F/Fig.5F_Gel_cropped.tif]

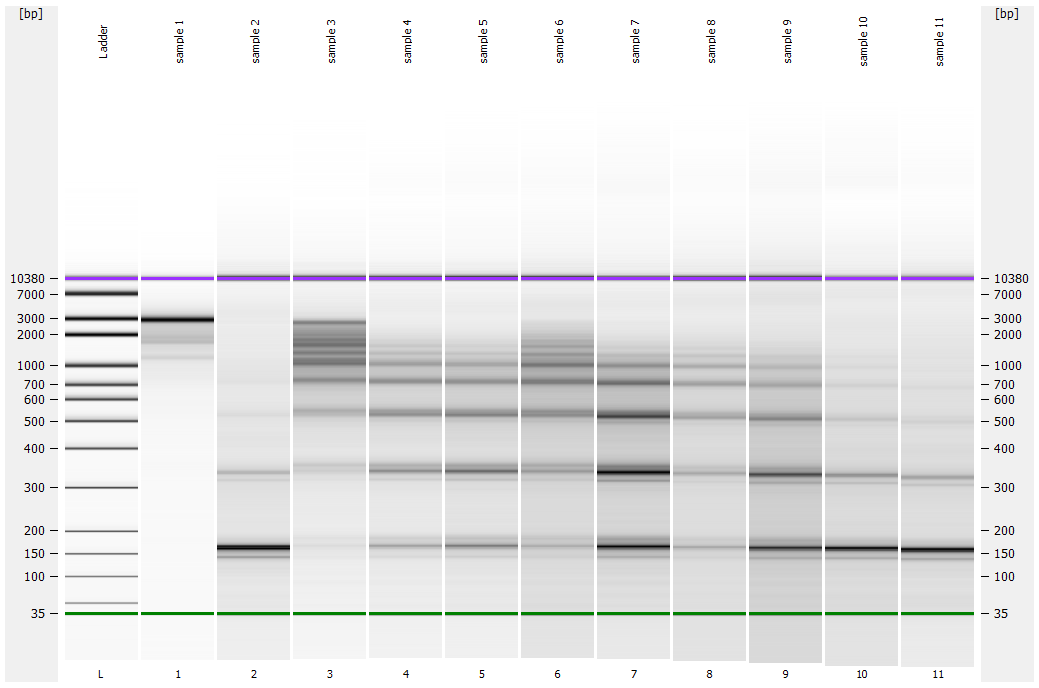

Supplement: Supplementary file 7 — Source data Fig. 5 [file 44318_2025_594_MOESM7_ESM.zip › Figure 5/Figure_5F/Fig.5F_Gel.tif]

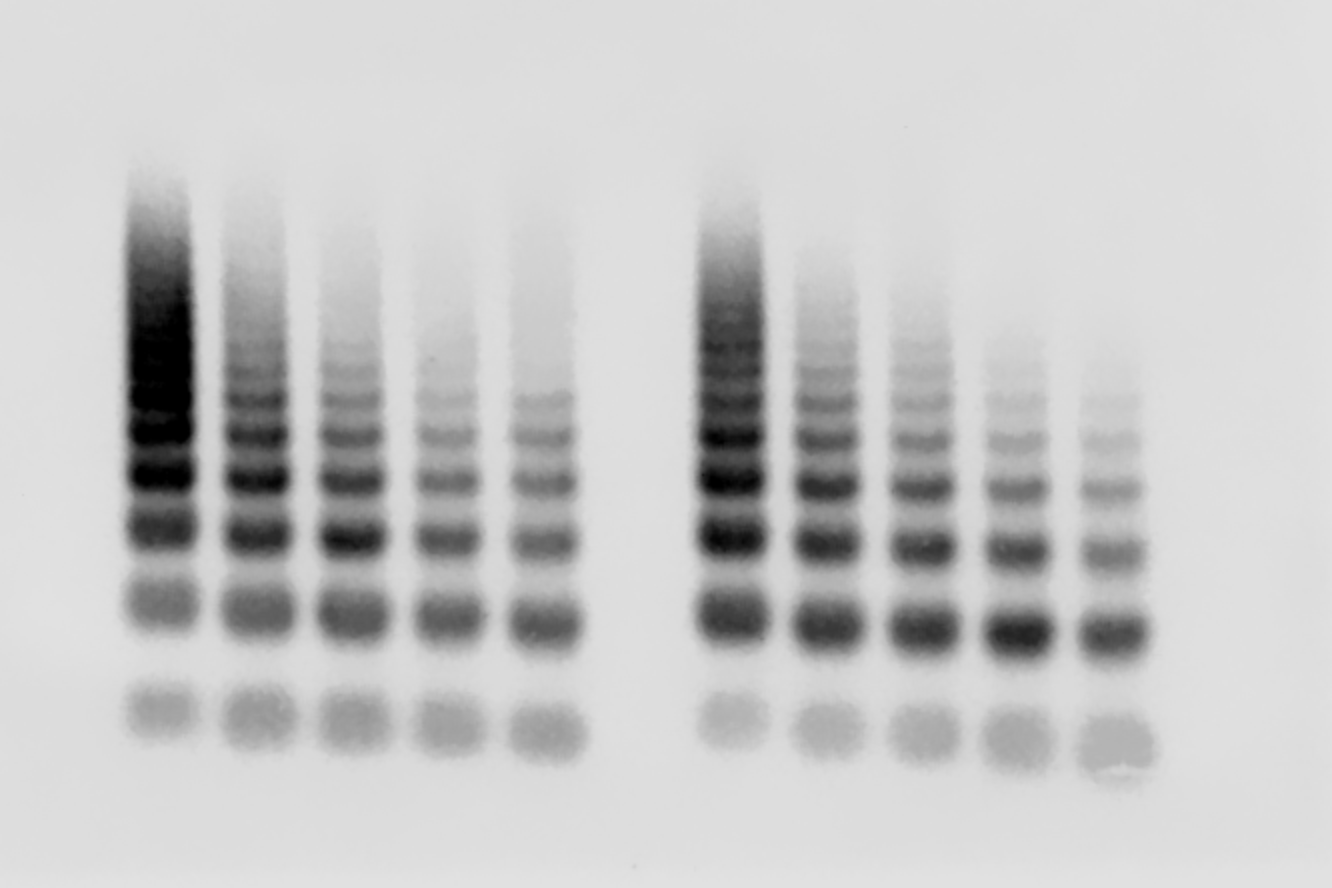

Supplement: Supplementary file 8 — Source data Fig. 6 [file 44318_2025_594_MOESM8_ESM.zip › Figure 6/Fig6A/Fig6A_aSat_INCENP_MNase_3rd-trial_HighExpose.tif]

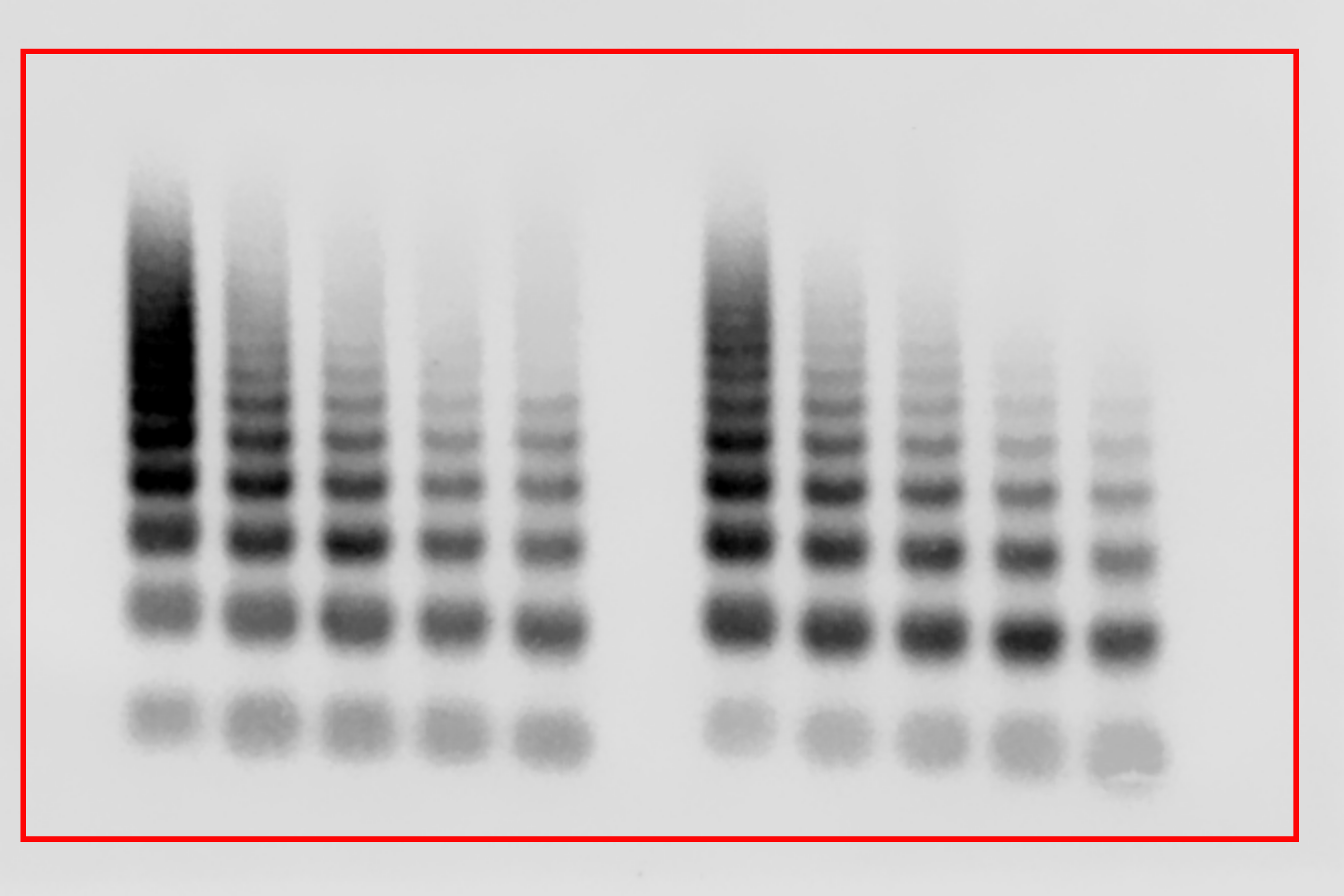

Supplement: Supplementary file 8 — Source data Fig. 6 [file 44318_2025_594_MOESM8_ESM.zip › Figure 6/Fig6A/Fig6A_cropped_area.tiff]

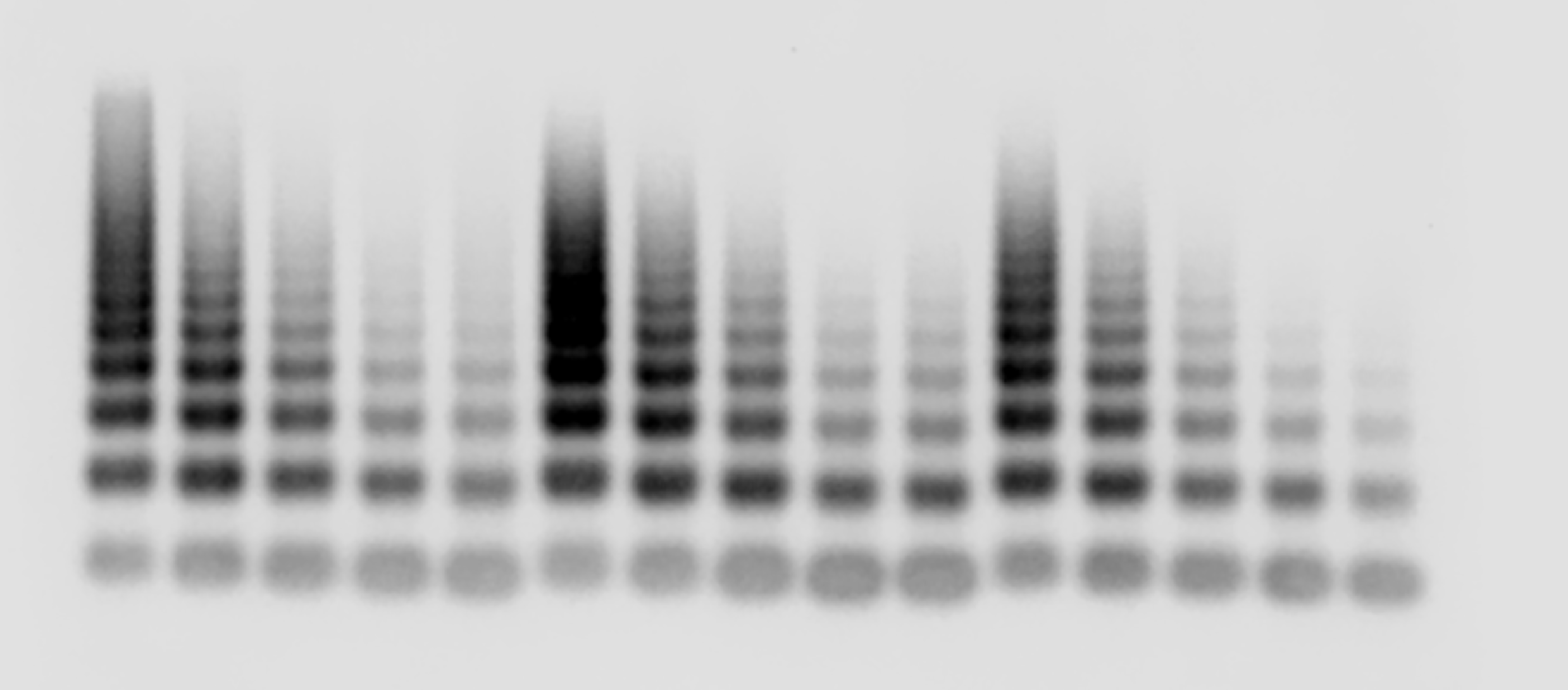

Supplement: Supplementary file 8 — Source data Fig. 6 [file 44318_2025_594_MOESM8_ESM.zip › Figure 6/Fig6B/Fig6B_aSat_Boreali_rep1.tif]

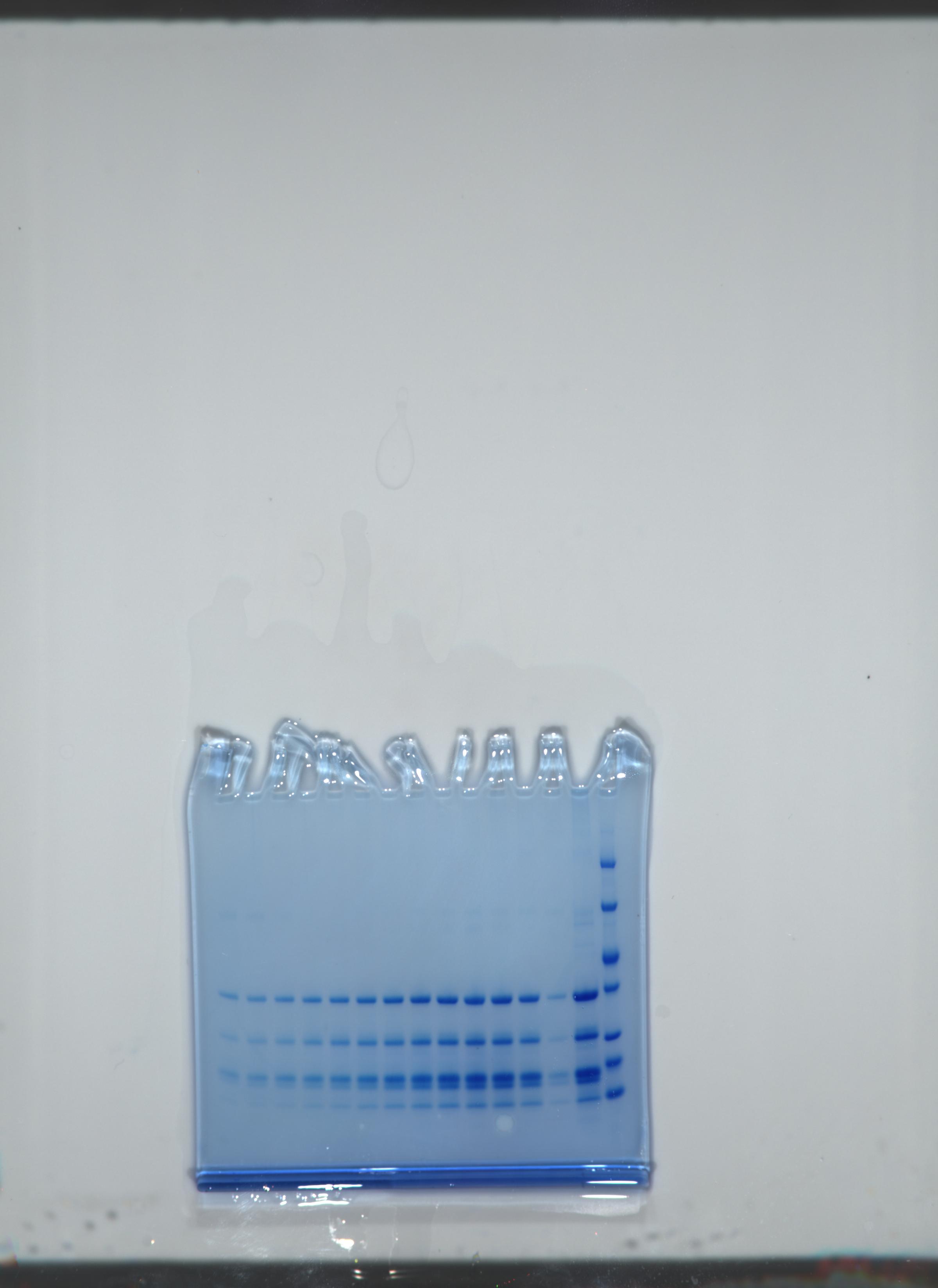

Supplement: Supplementary file 9 — EV Figure Source Data [file 44318_2025_594_MOESM9_ESM.zip › Source_Data_EV_Figures/Fig_EV1/Fig_EV1C/Fig_EV1C_210325_CPC_1_190_NCP_SEC 20250324_173828_Co_Gel.jpg]

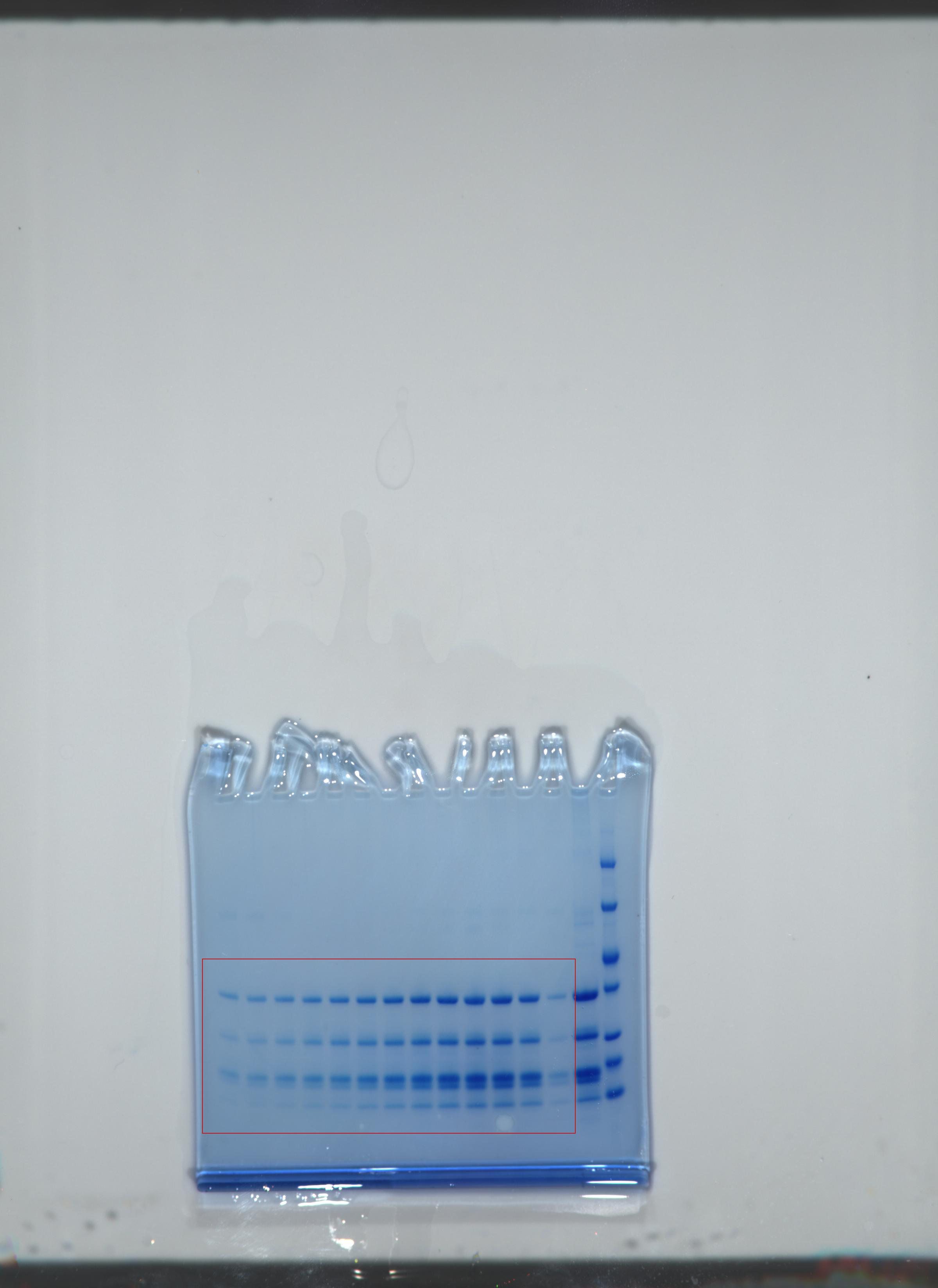

Supplement: Supplementary file 9 — EV Figure Source Data [file 44318_2025_594_MOESM9_ESM.zip › Source_Data_EV_Figures/Fig_EV1/Fig_EV1C/Fig_EV1C_210325_CPC_1_190_NCP_SEC 20250324_173828_Co_Gel_crop.tif]

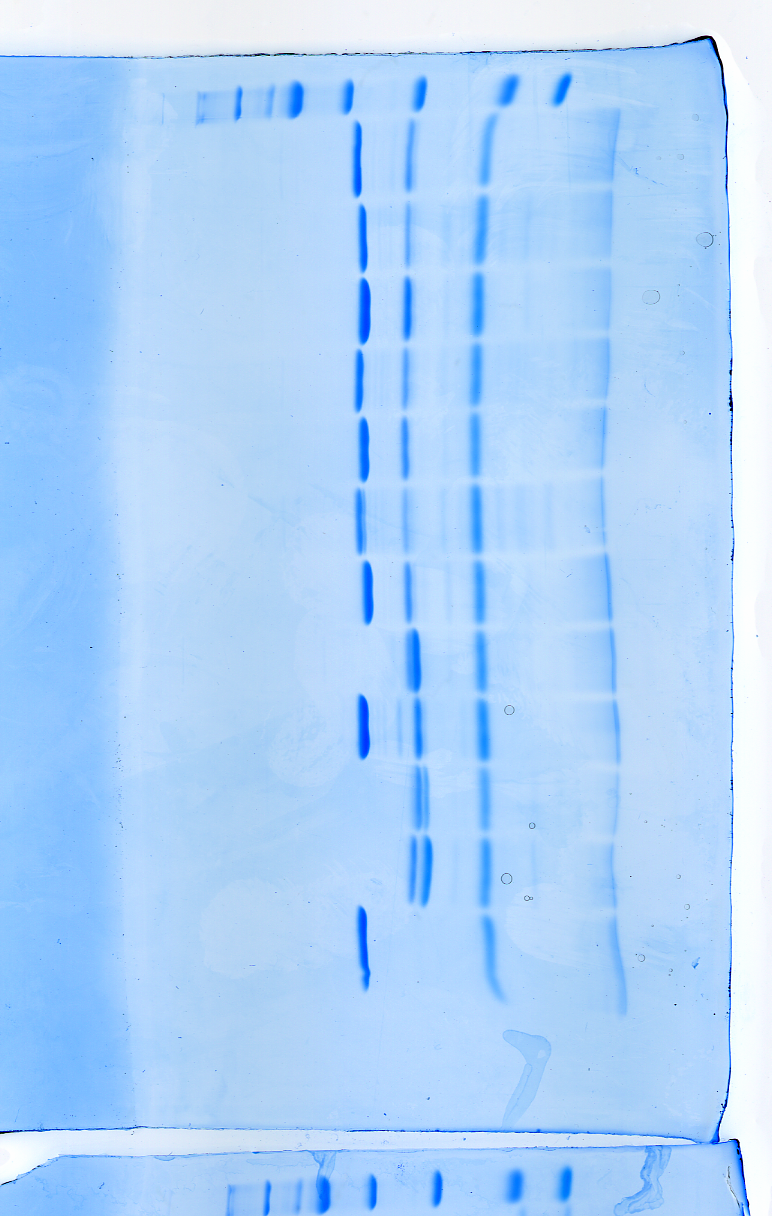

Supplement: Supplementary file 9 — EV Figure Source Data [file 44318_2025_594_MOESM9_ESM.zip › Source_Data_EV_Figures/Fig_EV5/Fig_EV5C_140825_CPC_constrcts_sample_15percentgel099.tif]

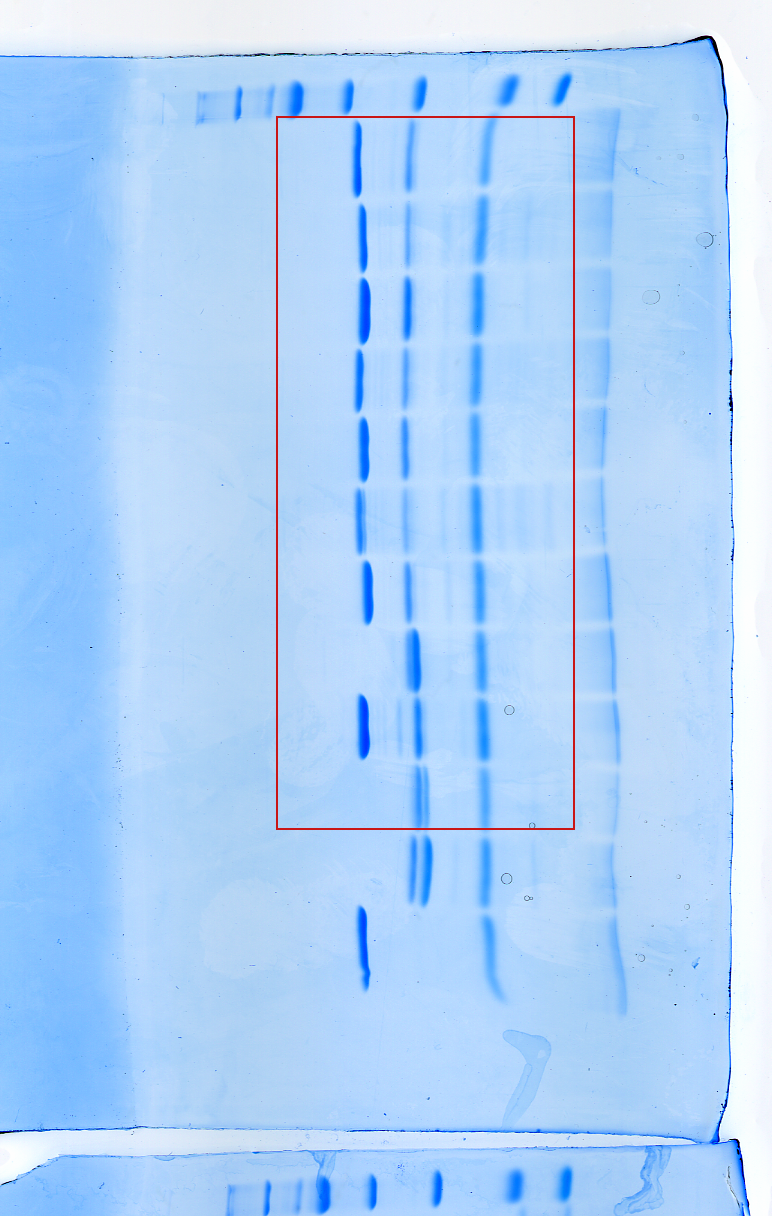

Supplement: Supplementary file 9 — EV Figure Source Data [file 44318_2025_594_MOESM9_ESM.zip › Source_Data_EV_Figures/Fig_EV5/Fig_EV5C_140825_CPC_constrcts_sample_15percentgel099_crop.tif]

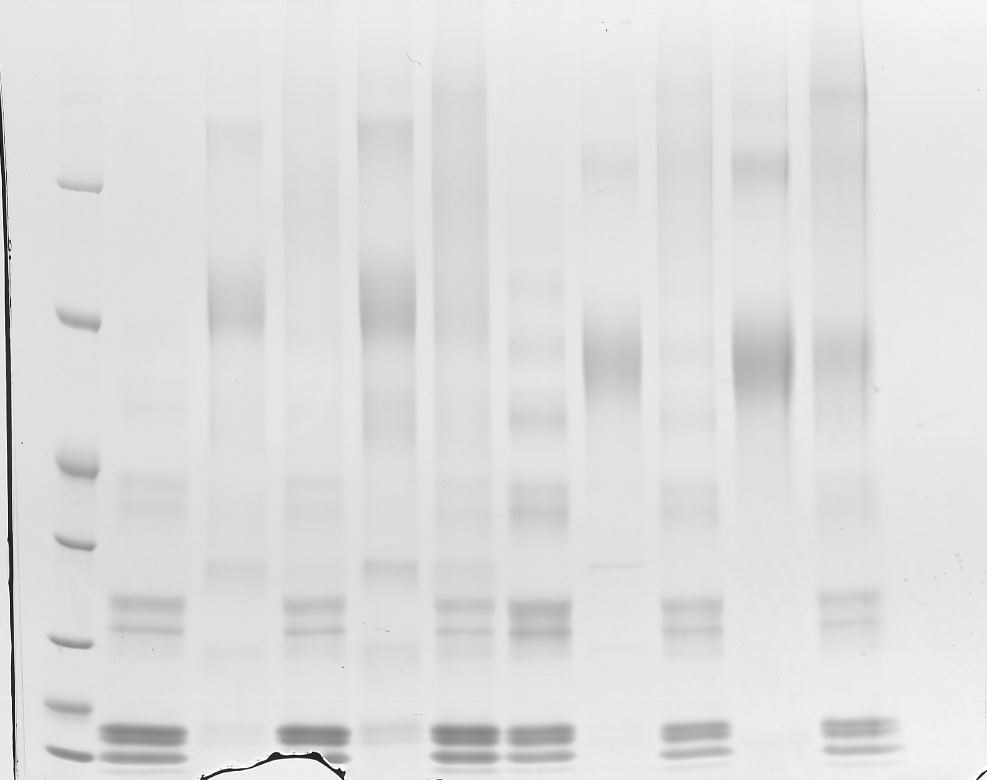

Supplement: Supplementary file 9 — EV Figure Source Data [file 44318_2025_594_MOESM9_ESM.zip › Source_Data_EV_Figures/Fig_EV6/Fig_EV6C_270325_CPC_NCP_EDC_crosslinking138.tif]

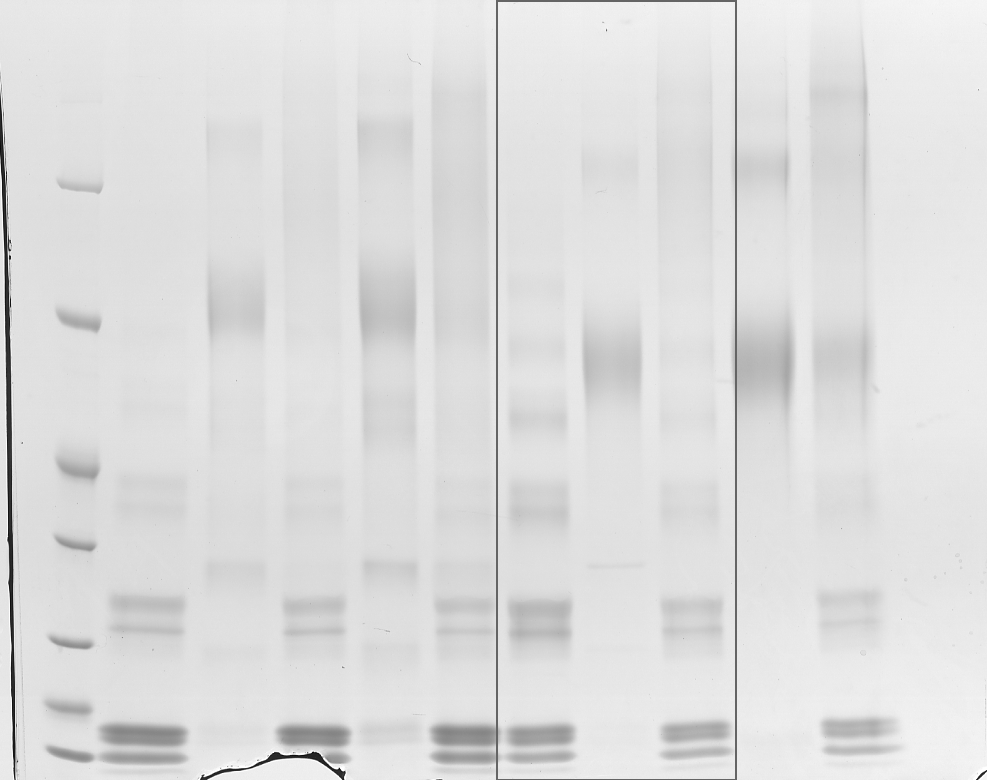

Supplement: Supplementary file 9 — EV Figure Source Data [file 44318_2025_594_MOESM9_ESM.zip › Source_Data_EV_Figures/Fig_EV6/Fig_EV6C_270325_CPC_NCP_EDC_crosslinking138_crop.tif]

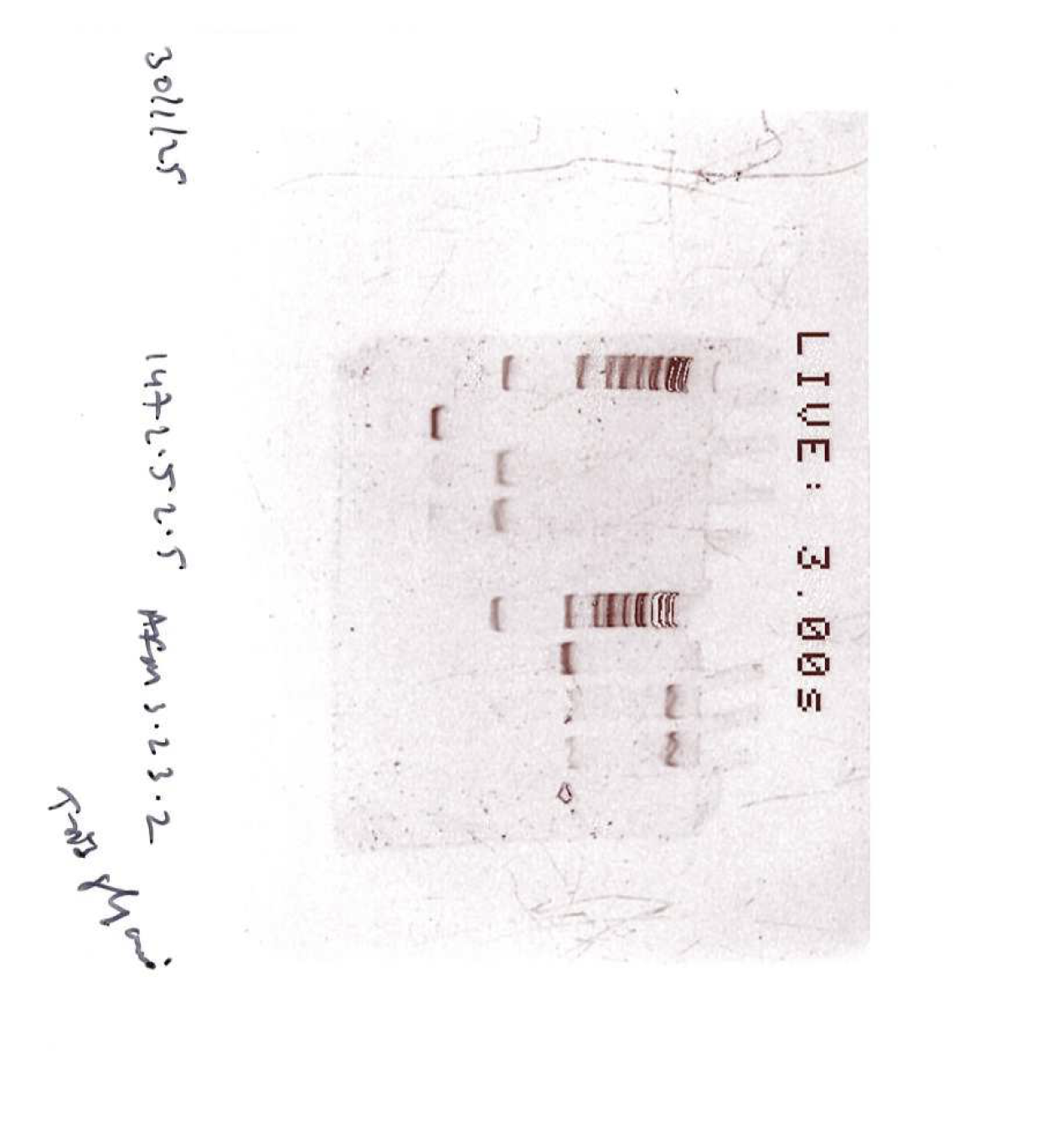

Supplement: Supplementary file 9 — EV Figure Source Data [file 44318_2025_594_MOESM9_ESM.zip › Source_Data_EV_Figures/Fig_EV7/Fig_EV7A/Fig_EV7A_AFMNCPs.png]

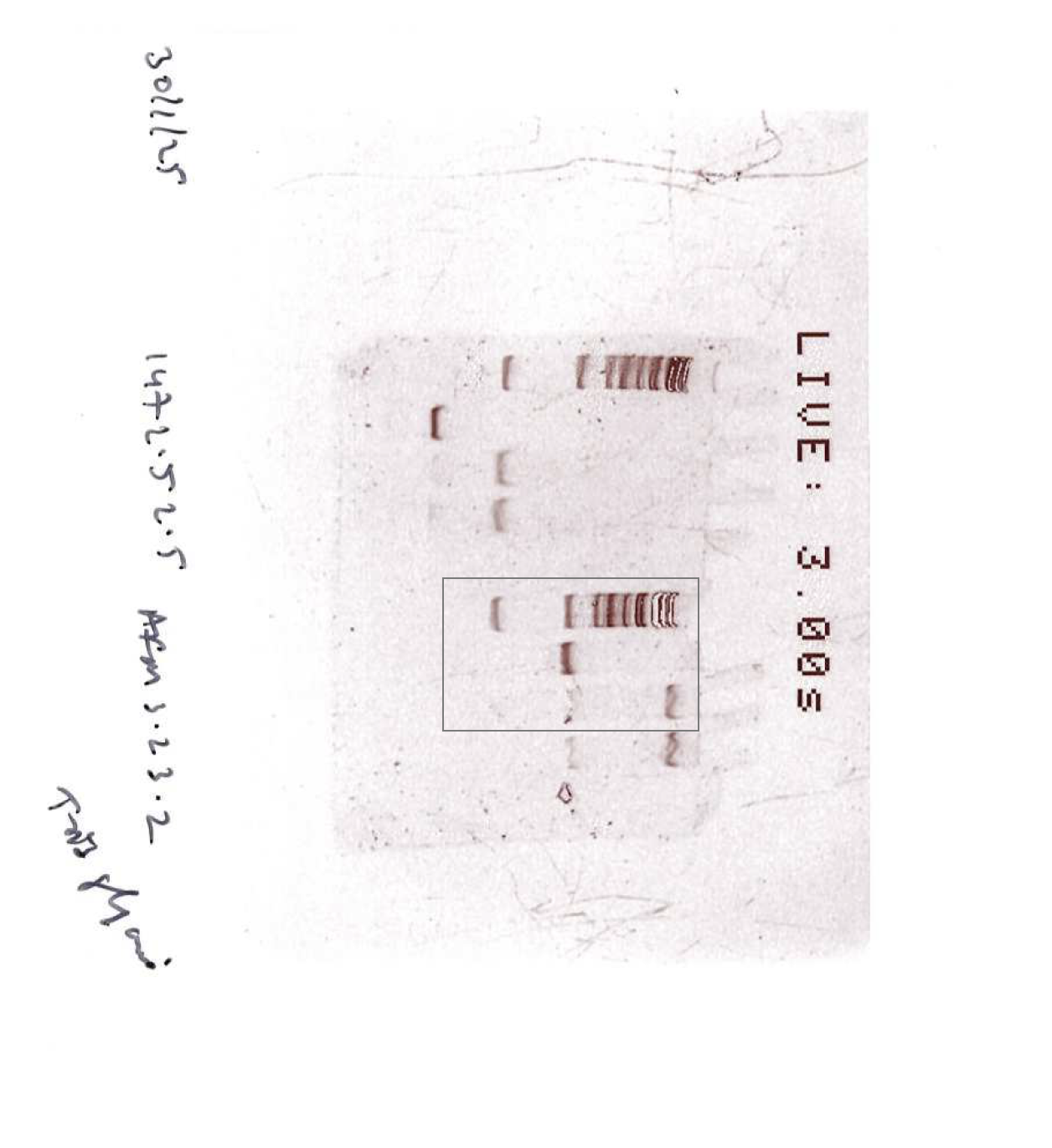

Supplement: Supplementary file 9 — EV Figure Source Data [file 44318_2025_594_MOESM9_ESM.zip › Source_Data_EV_Figures/Fig_EV7/Fig_EV7A/Fig_EV7A_AFMNCPs_crop.tif]

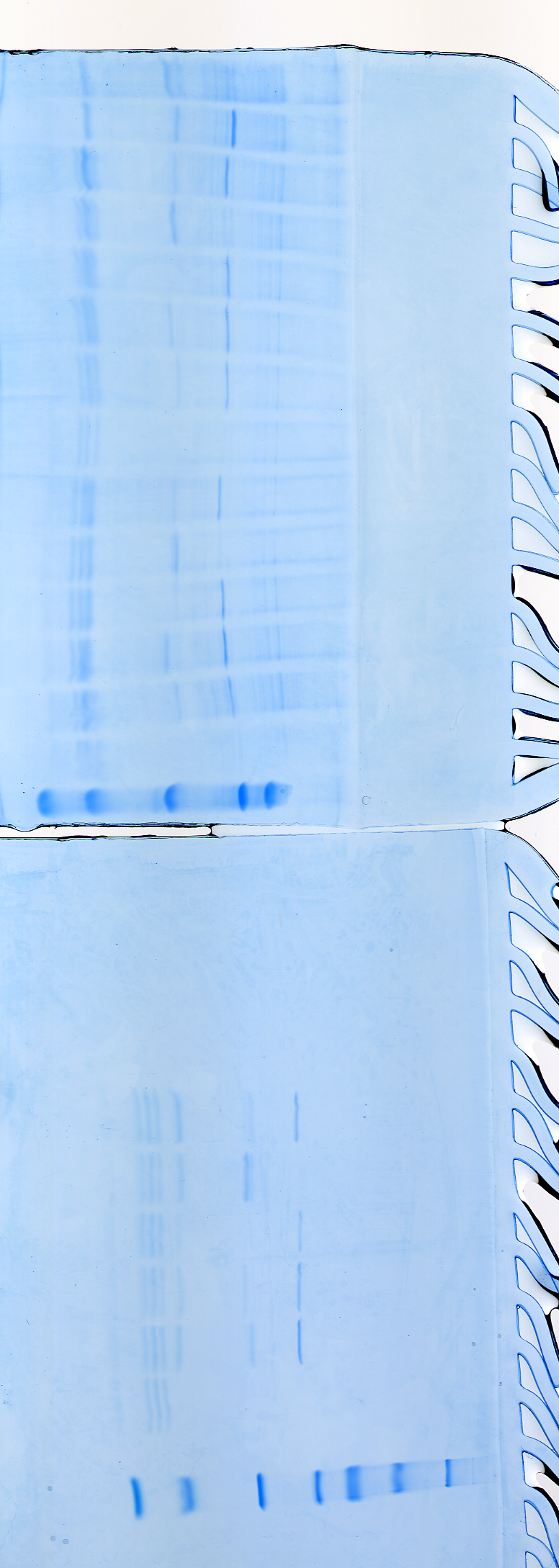

Supplement: Supplementary file 9 — EV Figure Source Data [file 44318_2025_594_MOESM9_ESM.zip › Source_Data_EV_Figures/Fig_EV8/Fig_EV8A/Fig_EV8A_090525_and_140525_mnase_inputs_17percent049.tif]

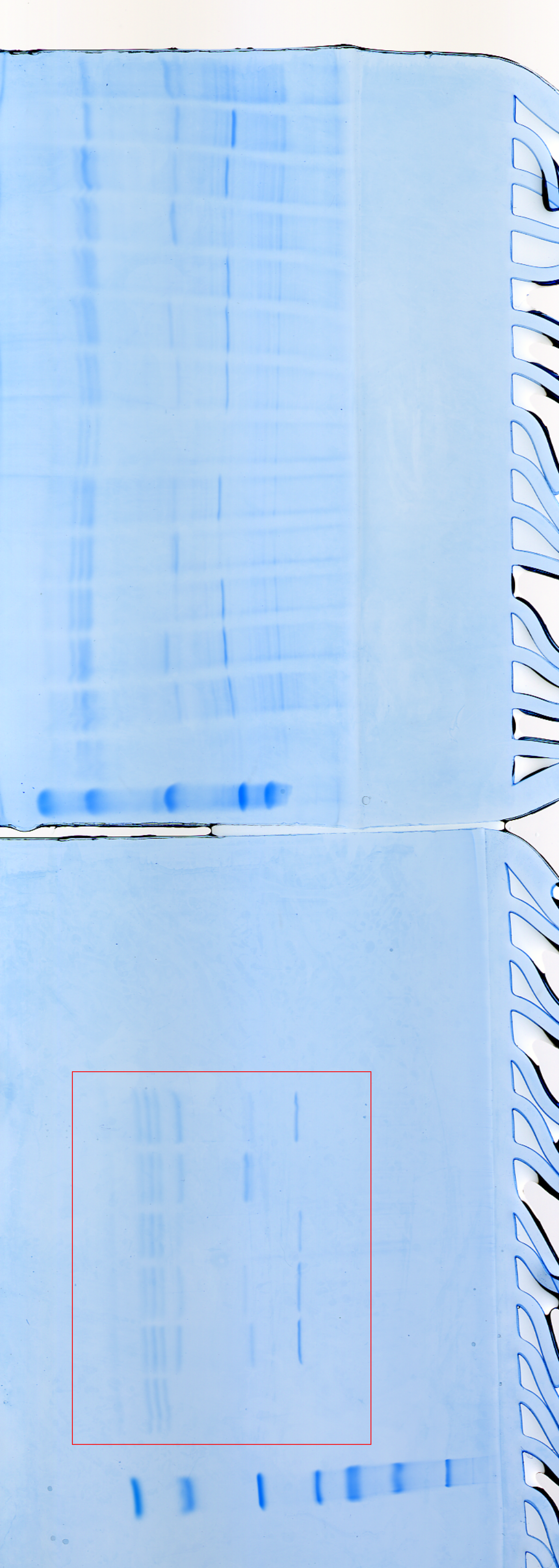

Supplement: Supplementary file 9 — EV Figure Source Data [file 44318_2025_594_MOESM9_ESM.zip › Source_Data_EV_Figures/Fig_EV8/Fig_EV8A/Fig_EV8A_090525_and_140525_mnase_inputs_17percent_crop.tiff]

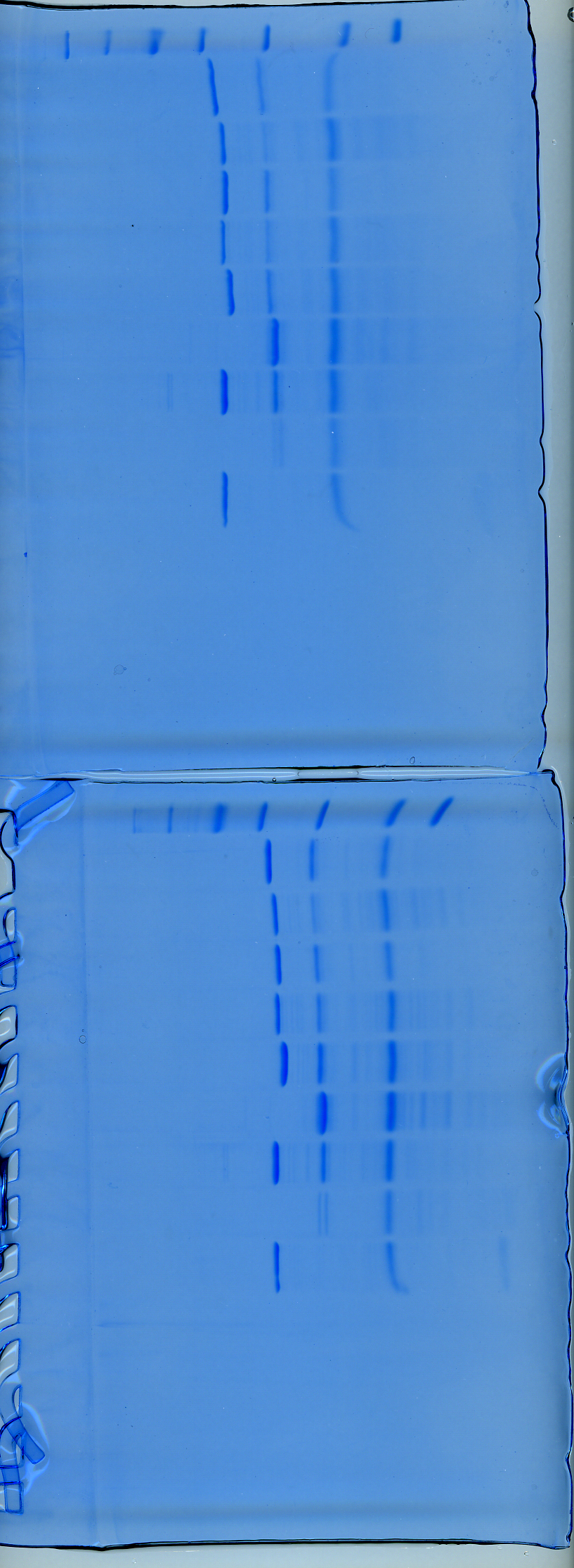

Supplement: Supplementary file 9 — EV Figure Source Data [file 44318_2025_594_MOESM9_ESM.zip › Source_Data_EV_Figures/Fig_EV8/Fig_EV8B/Fig_EV8B_02_09_and_03_09_25_CPC_MNase_controls_15percent001.tif]

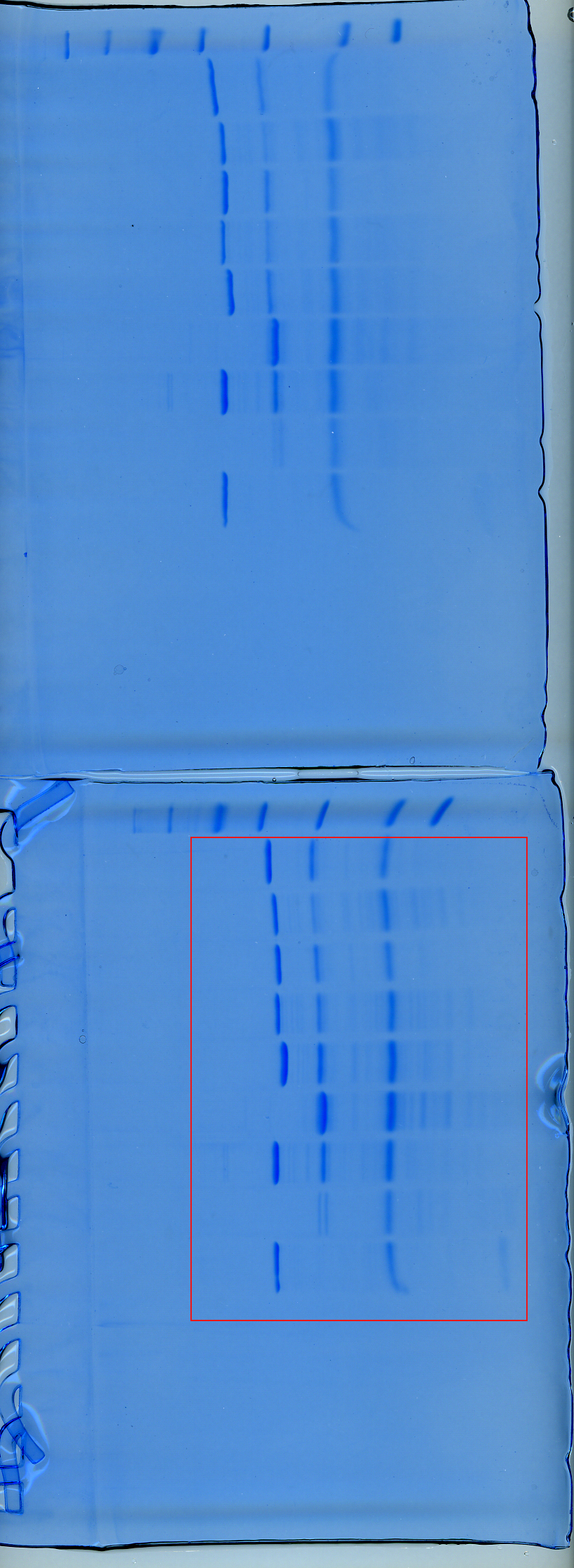

Supplement: Supplementary file 9 — EV Figure Source Data [file 44318_2025_594_MOESM9_ESM.zip › Source_Data_EV_Figures/Fig_EV8/Fig_EV8B/Fig_EV8B_02_09_and_03_09_25_CPC_MNase_controls_15percent001_crop.tif]

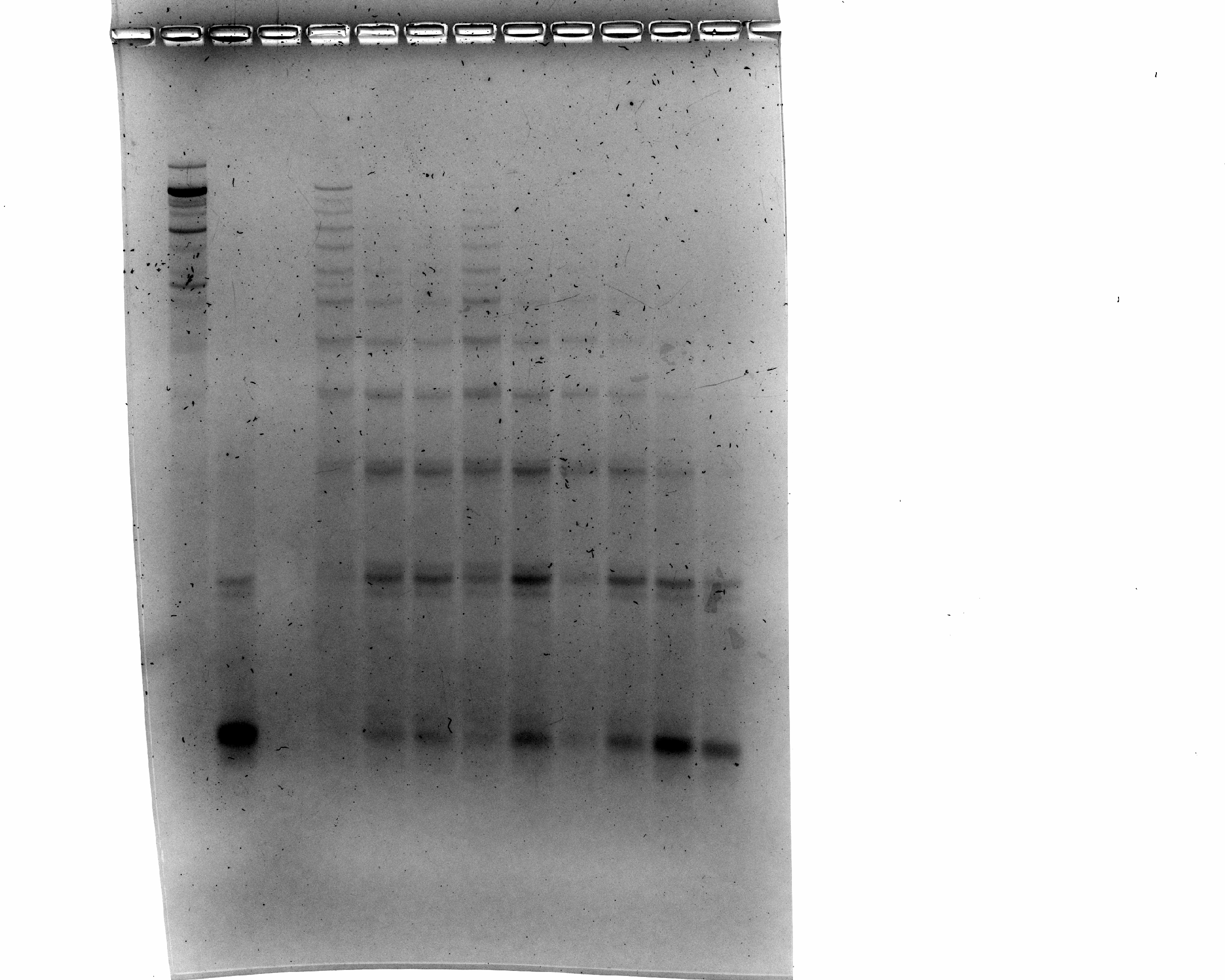

Supplement: Supplementary file 9 — EV Figure Source Data [file 44318_2025_594_MOESM9_ESM.zip › Source_Data_EV_Figures/Fig_EV8/Fig_EV8D/Fig_EV8D_Array_Agarose_JP lab 2025-09-03 15h27m15s(SYBR® Safe).tif]

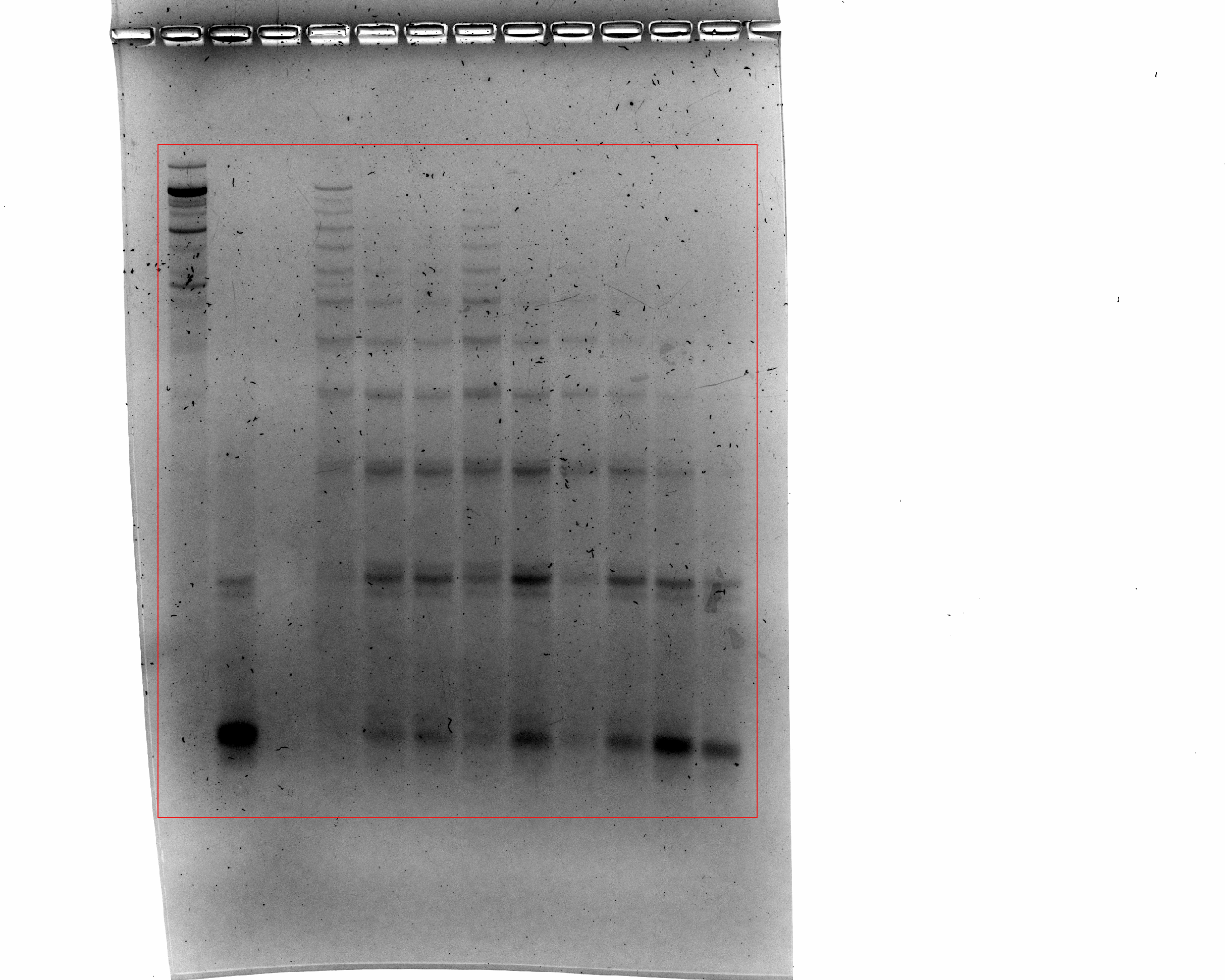

Supplement: Supplementary file 9 — EV Figure Source Data [file 44318_2025_594_MOESM9_ESM.zip › Source_Data_EV_Figures/Fig_EV8/Fig_EV8D/Fig_EV8D_Array_Agarose_JP lab 2025-09-03 15h27m15s(SYBR® Safe)_crop.tif]

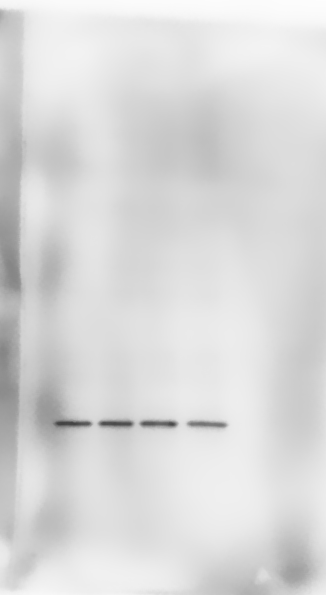

Supplement: Supplementary file 9 — EV Figure Source Data [file 44318_2025_594_MOESM9_ESM.zip › Source_Data_EV_Figures/Fig_EV9/Fig_EV9A/anti-Aurora B(EV9A).tif]

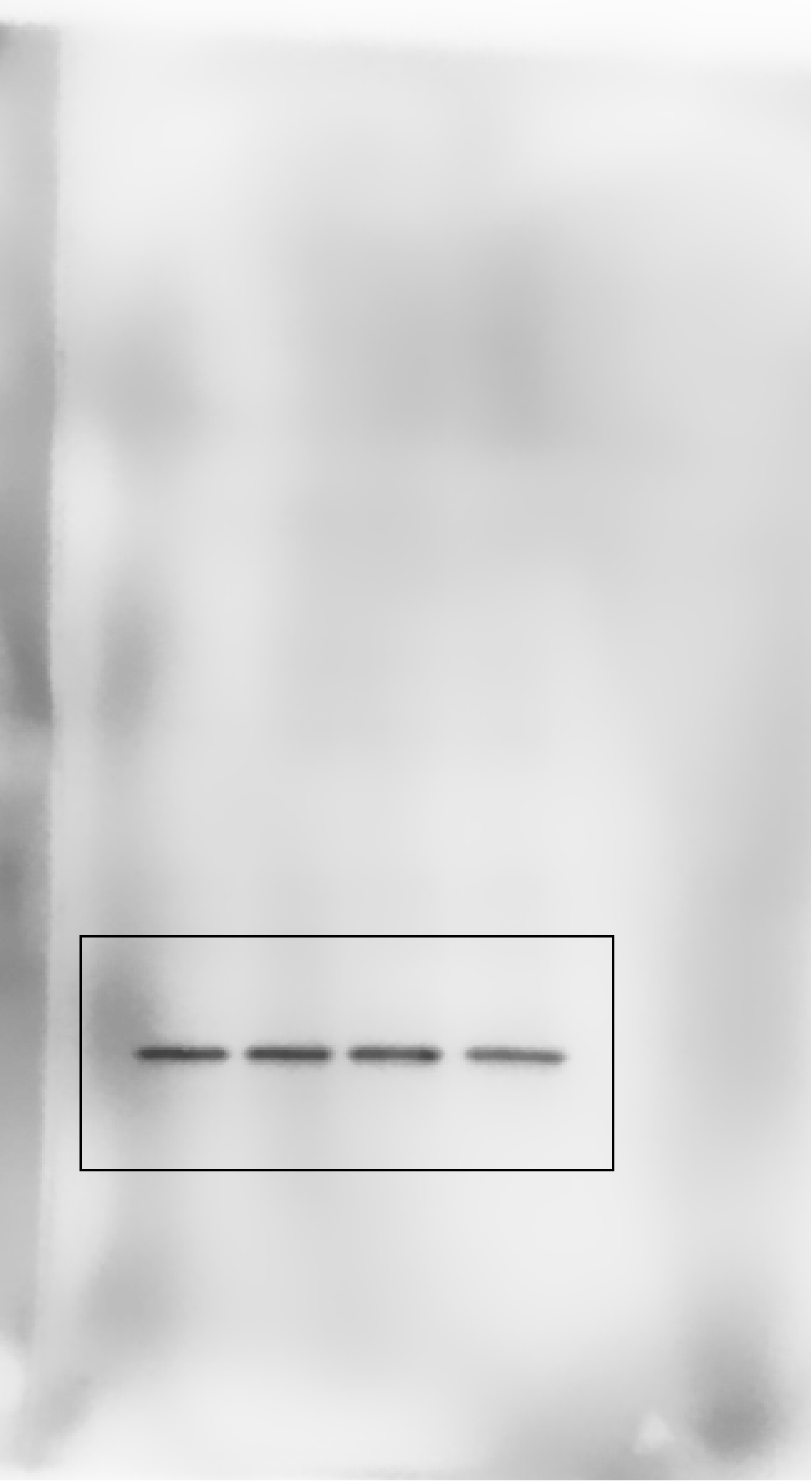

Supplement: Supplementary file 9 — EV Figure Source Data [file 44318_2025_594_MOESM9_ESM.zip › Source_Data_EV_Figures/Fig_EV9/Fig_EV9A/anti-Aurora B(EV9A)_crop.tif]

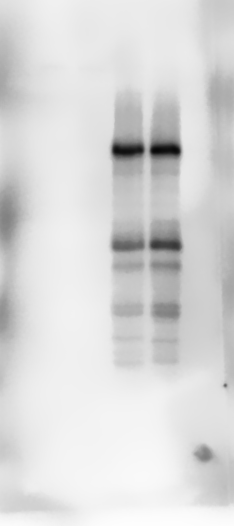

Supplement: Supplementary file 9 — EV Figure Source Data [file 44318_2025_594_MOESM9_ESM.zip › Source_Data_EV_Figures/Fig_EV9/Fig_EV9A/anti-FLAG(EV9A).tif]

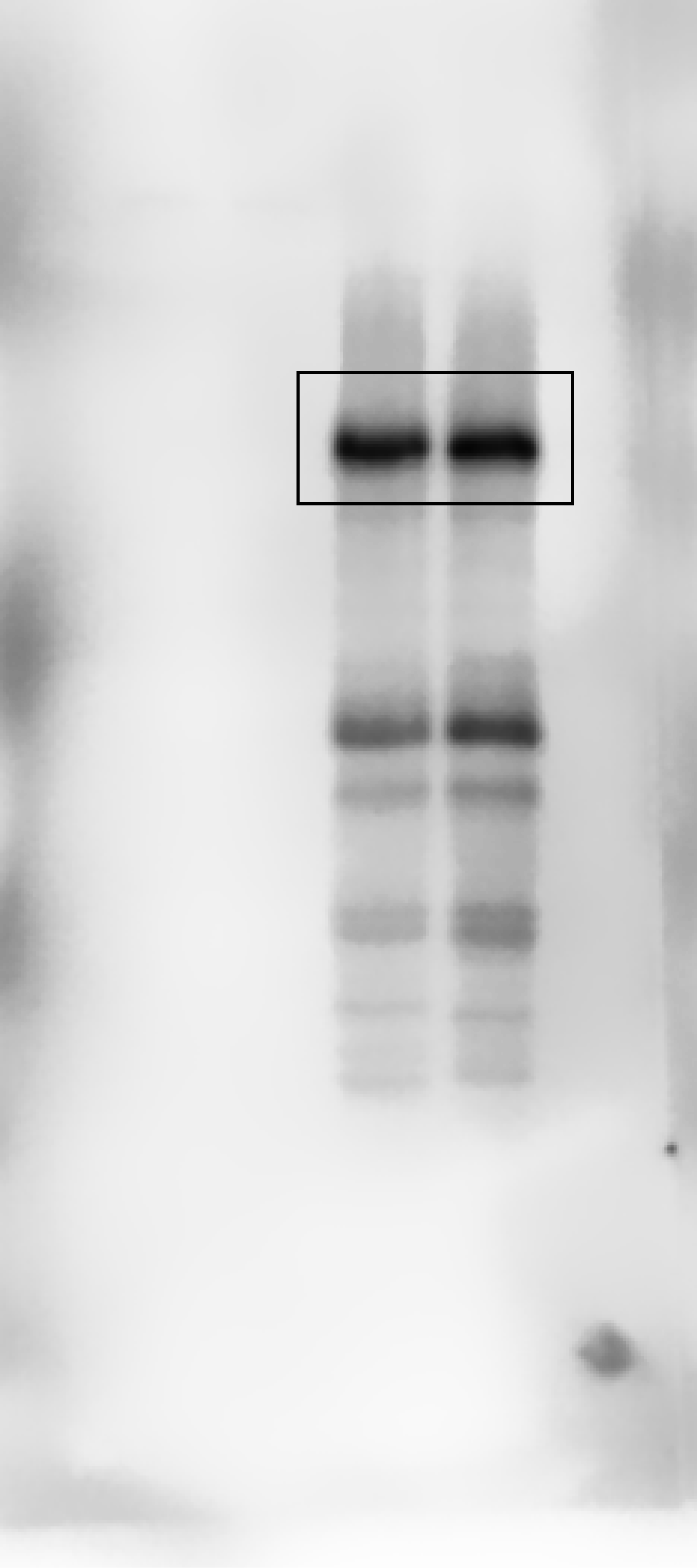

Supplement: Supplementary file 9 — EV Figure Source Data [file 44318_2025_594_MOESM9_ESM.zip › Source_Data_EV_Figures/Fig_EV9/Fig_EV9A/anti-FLAG(EV9A)_crop.tif]

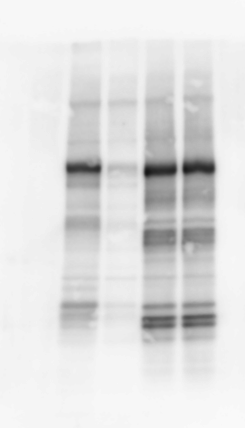

Supplement: Supplementary file 9 — EV Figure Source Data [file 44318_2025_594_MOESM9_ESM.zip › Source_Data_EV_Figures/Fig_EV9/Fig_EV9A/anti-INCENP(EV9A).tif]

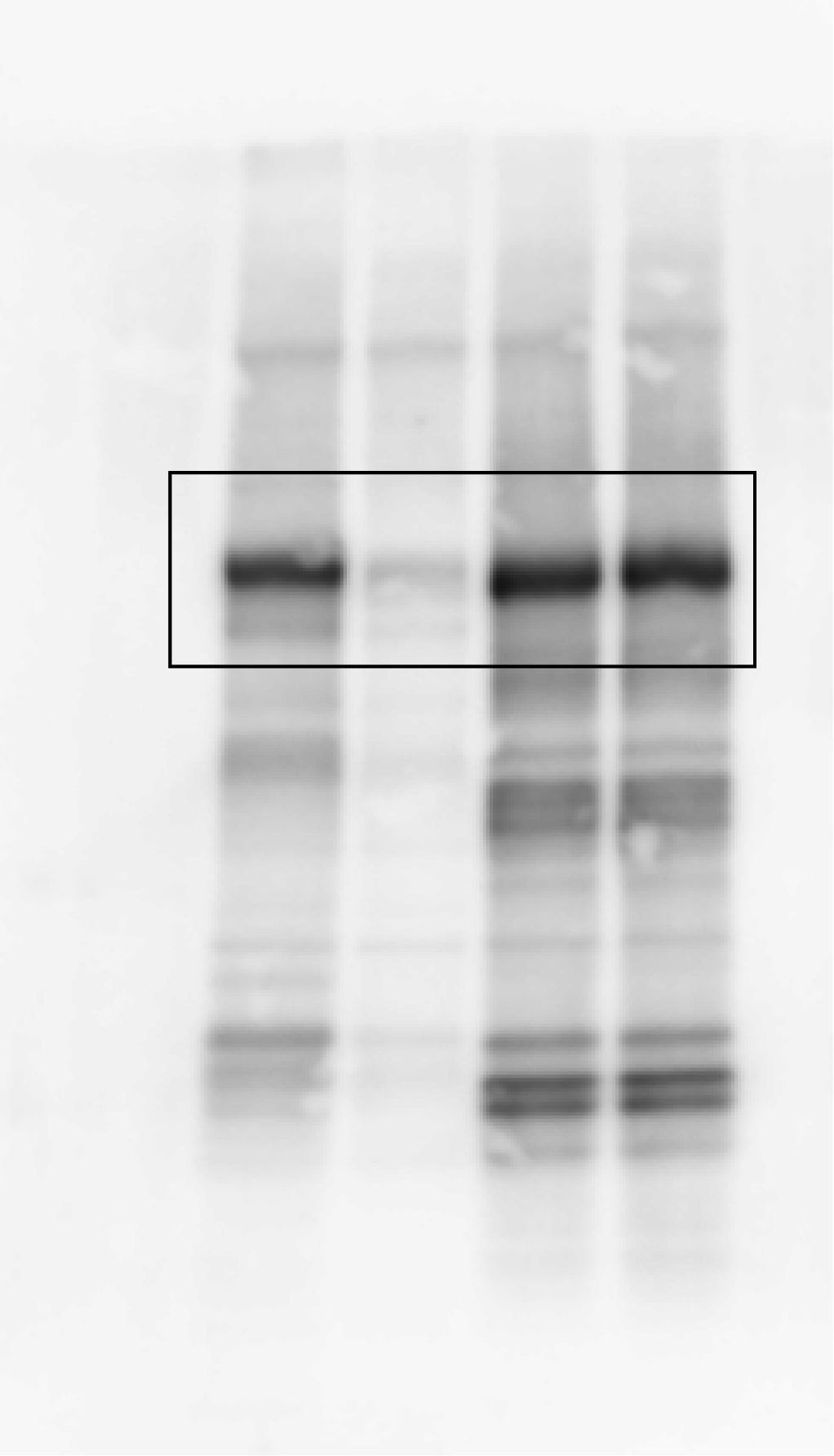

Supplement: Supplementary file 9 — EV Figure Source Data [file 44318_2025_594_MOESM9_ESM.zip › Source_Data_EV_Figures/Fig_EV9/Fig_EV9A/anti-INCENP(EV9A)_crop.tif]

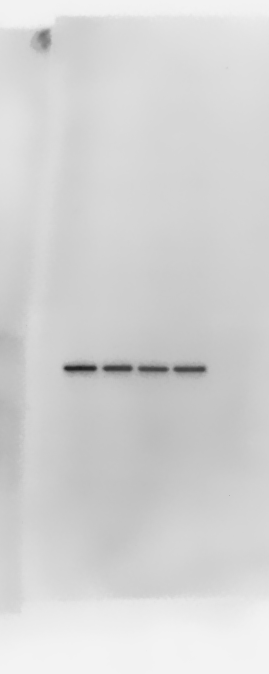

Supplement: Supplementary file 9 — EV Figure Source Data [file 44318_2025_594_MOESM9_ESM.zip › Source_Data_EV_Figures/Fig_EV9/Fig_EV9A/anti-Tubulin(EV9A).tif]

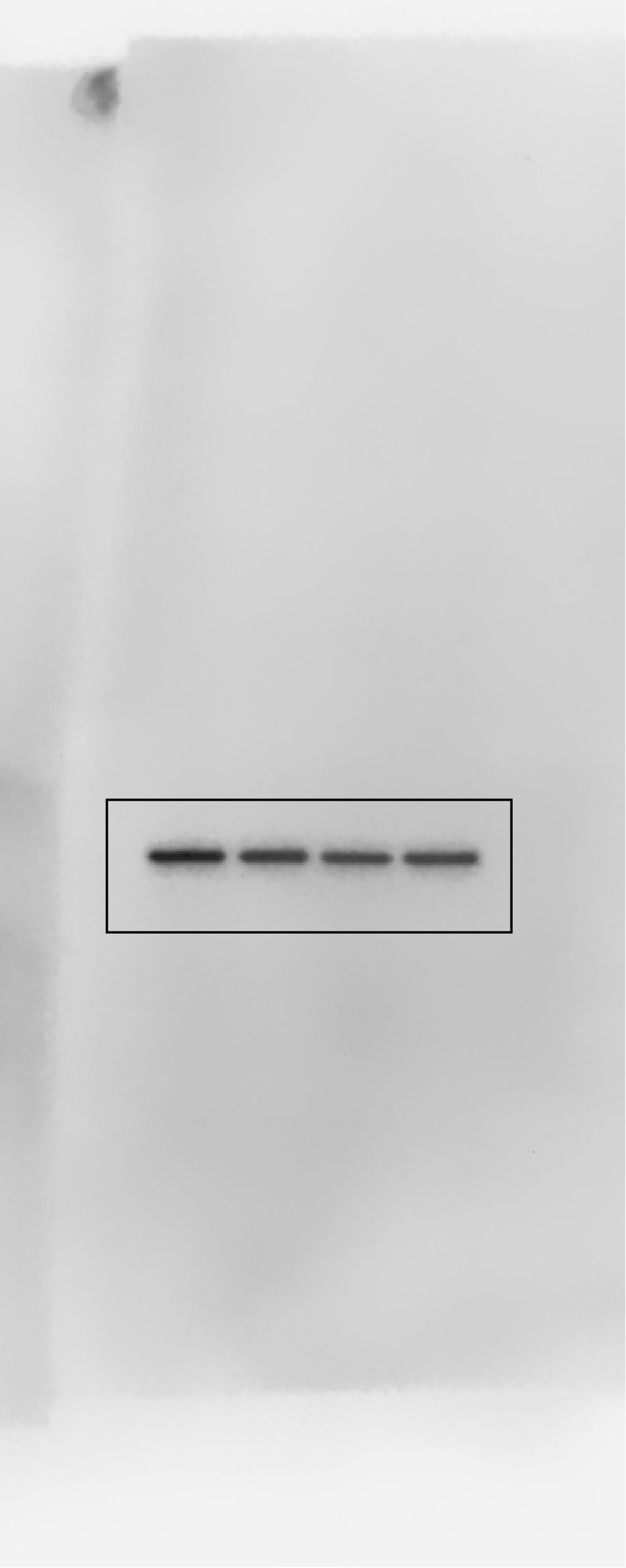

Supplement: Supplementary file 9 — EV Figure Source Data [file 44318_2025_594_MOESM9_ESM.zip › Source_Data_EV_Figures/Fig_EV9/Fig_EV9A/anti-Tubulin(EV9A)_crop.tif]

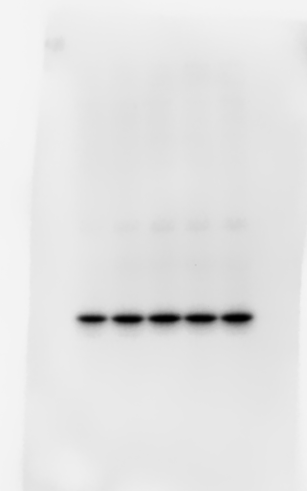

Supplement: Supplementary file 9 — EV Figure Source Data [file 44318_2025_594_MOESM9_ESM.zip › Source_Data_EV_Figures/Fig_EV9/Fig_EV9B/anti-Aurora B(EV9B).tif]

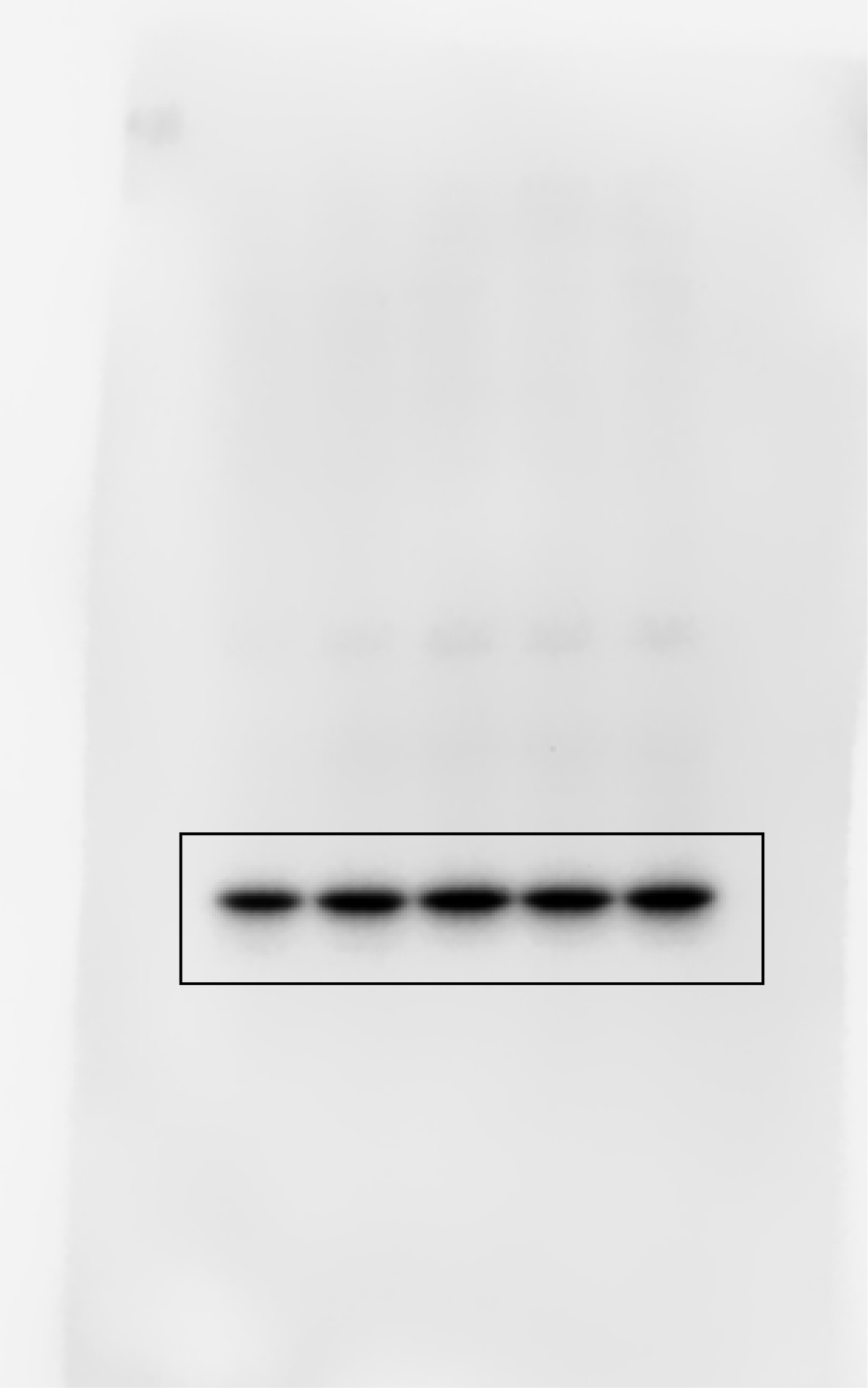

Supplement: Supplementary file 9 — EV Figure Source Data [file 44318_2025_594_MOESM9_ESM.zip › Source_Data_EV_Figures/Fig_EV9/Fig_EV9B/anti-Aurora B(EV9B)_crop.tif]

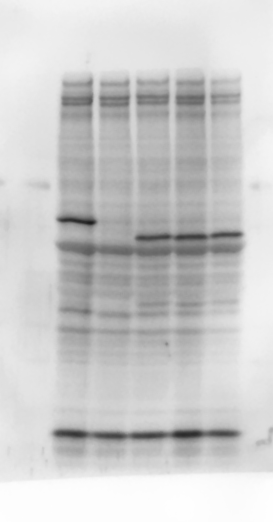

Supplement: Supplementary file 9 — EV Figure Source Data [file 44318_2025_594_MOESM9_ESM.zip › Source_Data_EV_Figures/Fig_EV9/Fig_EV9B/anti-Borealin(EV9B).tif]

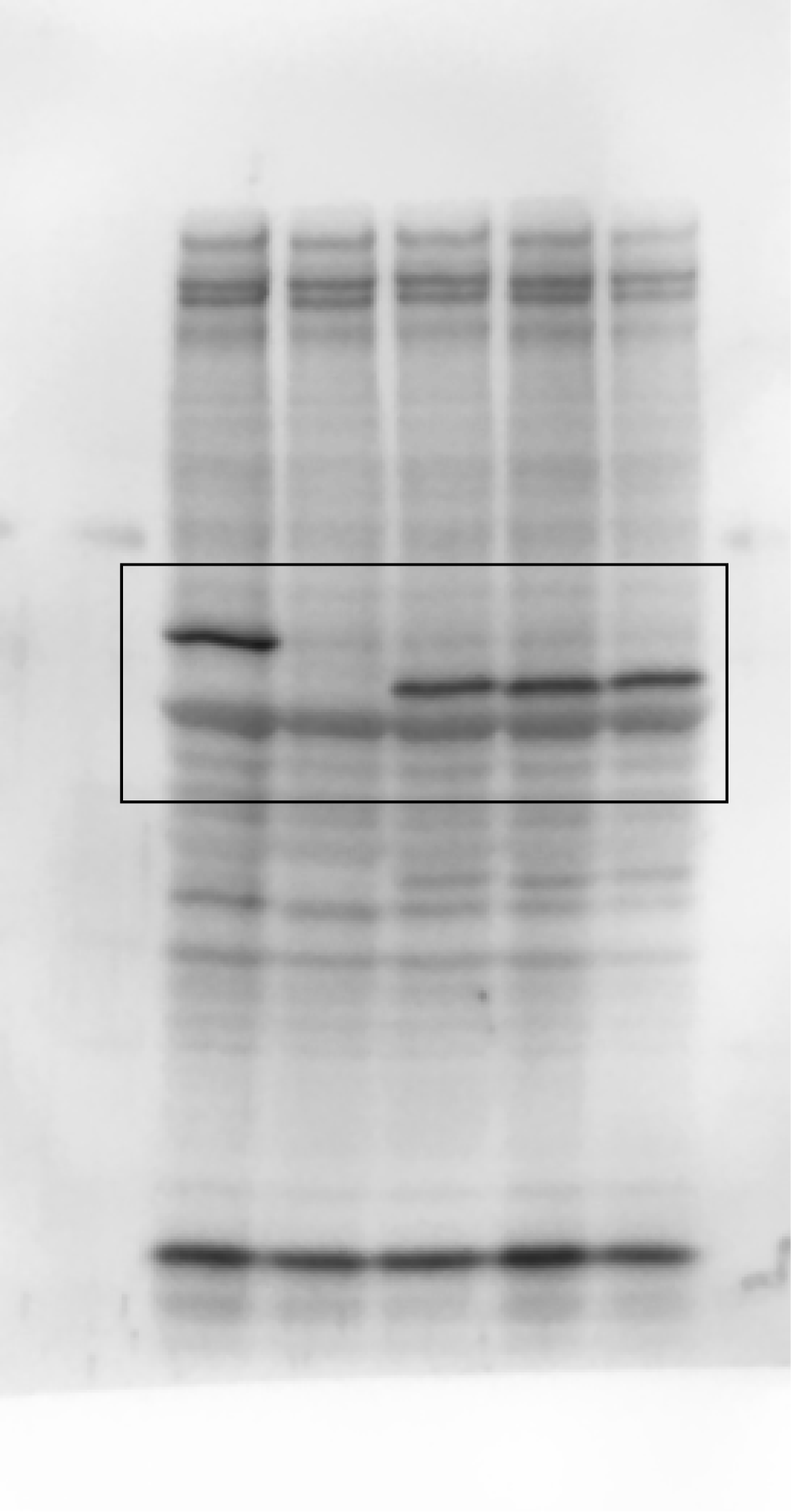

Supplement: Supplementary file 9 — EV Figure Source Data [file 44318_2025_594_MOESM9_ESM.zip › Source_Data_EV_Figures/Fig_EV9/Fig_EV9B/anti-Borealin(EV9B)_crop.tif]

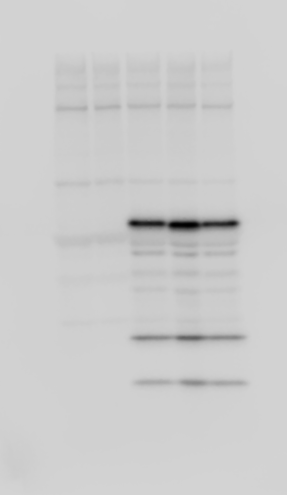

Supplement: Supplementary file 9 — EV Figure Source Data [file 44318_2025_594_MOESM9_ESM.zip › Source_Data_EV_Figures/Fig_EV9/Fig_EV9B/anti-mCherry(EV9B).tif]

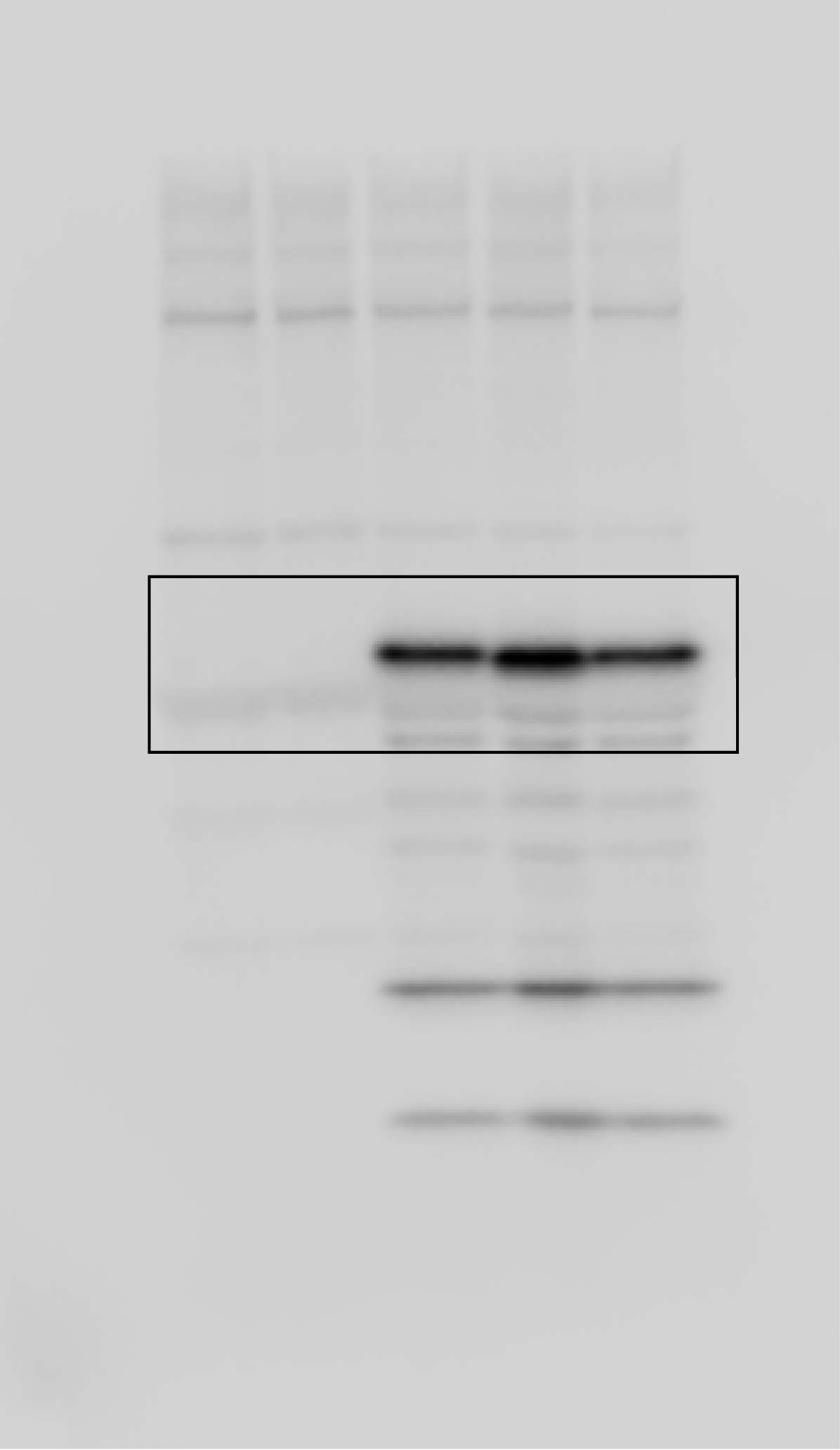

Supplement: Supplementary file 9 — EV Figure Source Data [file 44318_2025_594_MOESM9_ESM.zip › Source_Data_EV_Figures/Fig_EV9/Fig_EV9B/anti-mCherry(EV9B)_crop.tif]

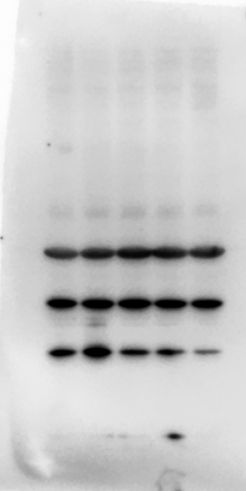

Supplement: Supplementary file 9 — EV Figure Source Data [file 44318_2025_594_MOESM9_ESM.zip › Source_Data_EV_Figures/Fig_EV9/Fig_EV9B/anti-Tubulin(EV9B).tif]

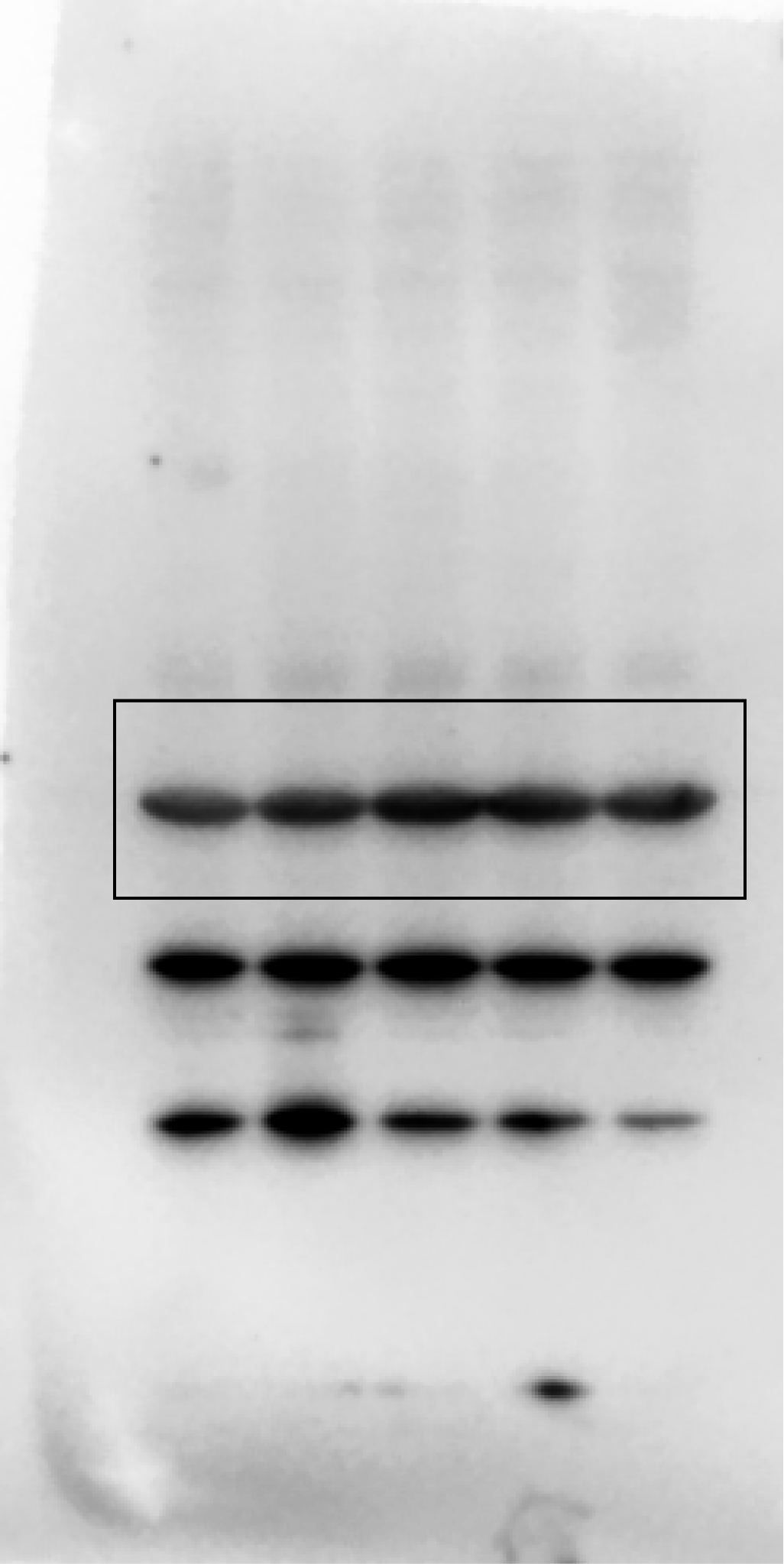

Supplement: Supplementary file 9 — EV Figure Source Data [file 44318_2025_594_MOESM9_ESM.zip › Source_Data_EV_Figures/Fig_EV9/Fig_EV9B/anti-Tubulin(EV9B)_crop.tif]

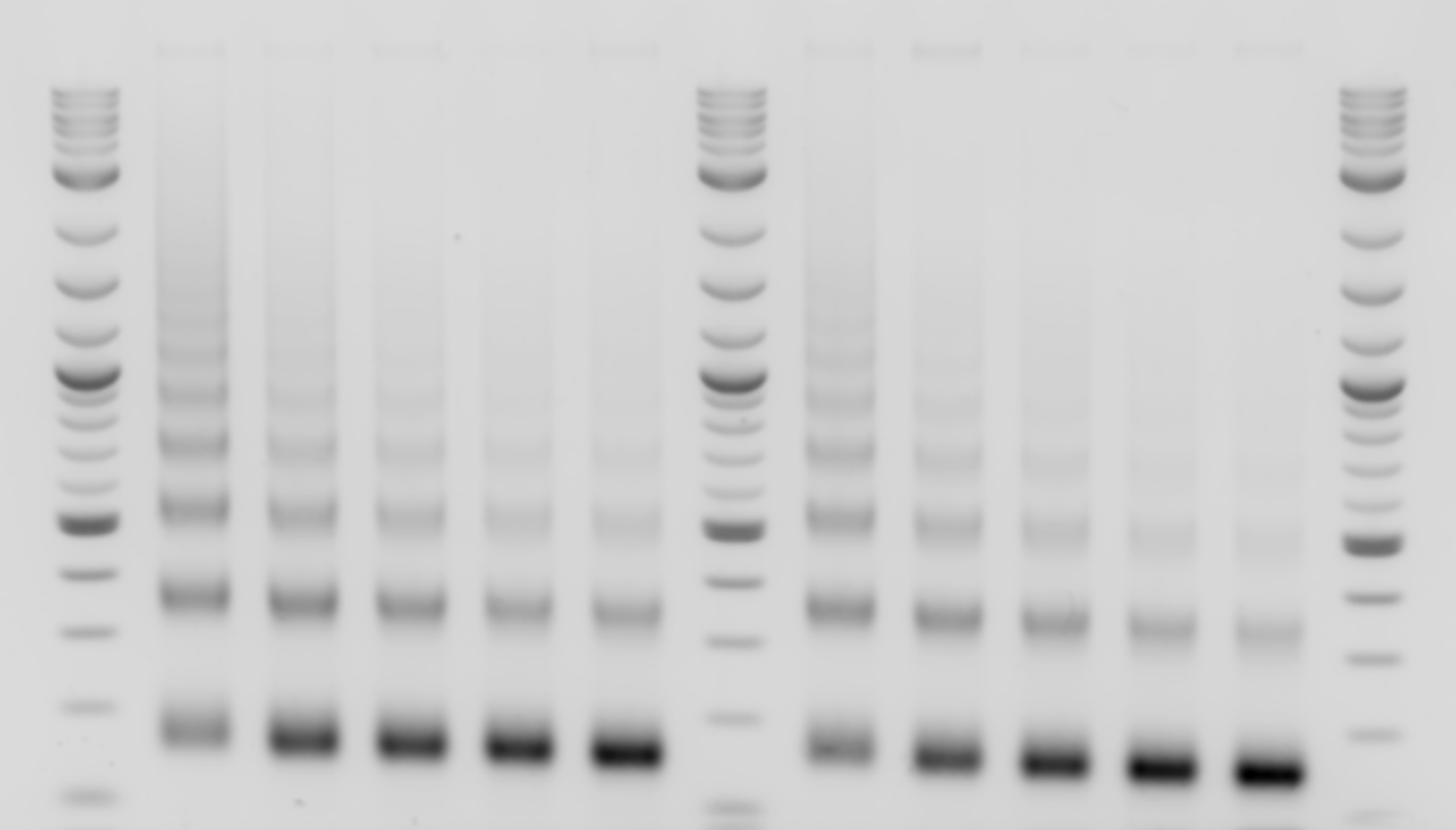

Supplement: Supplementary file 9 — EV Figure Source Data [file 44318_2025_594_MOESM9_ESM.zip › Source_Data_EV_Figures/Fig_EV9/Fig_EV9C/Fig_EV9C_EtBr_INCENP_MNase_3rd-trial.tif]

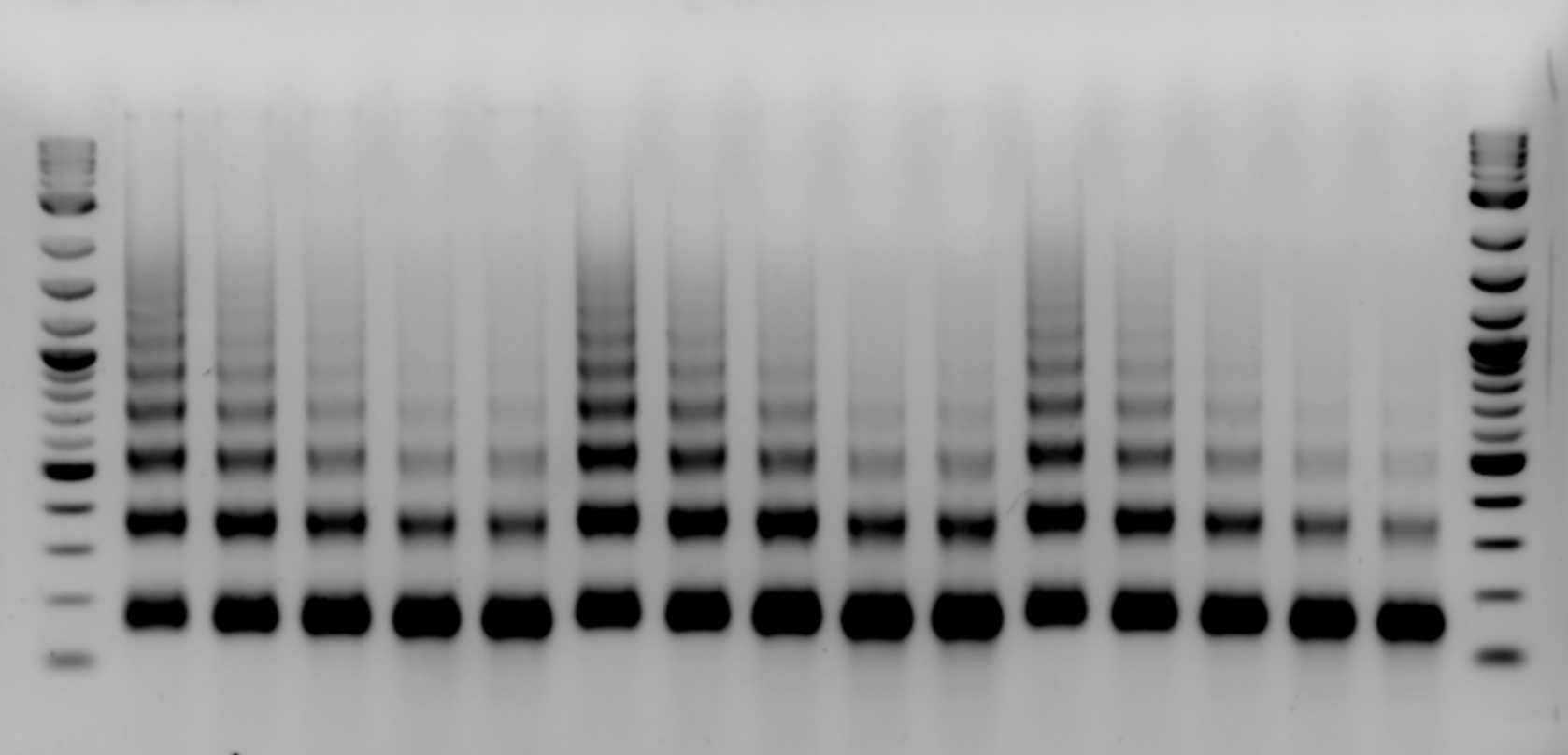

Supplement: Supplementary file 9 — EV Figure Source Data [file 44318_2025_594_MOESM9_ESM.zip › Source_Data_EV_Figures/Fig_EV9/Fig_EV9D/Fig_EV9D_EtBr_Borealin_rep1.tif]
