# Supplementary material for: Nucleosome interaction of the CPC secures centromeric chromatin integrity and chromosome segregation fidelity
Source: EMBO J. 2025 Oct 27;44(22):6556–97. doi: 10.1038/s44318-025-00594-y (PMC12624148; doi:10.1038/s44318-025-00594-y)
Supplement: Supplementary file 10 — Expanded View Figures [file 44318_2025_594_MOESM10_ESM.pdf]

Expanded View Figures

Figure EV1. Reconstitution and characterization of $CPC_{I1\text{-}190SB}$-NCP complex.

(**A**) Sequence alignment of Borealin orthologs from *Homo sapiens* (hs), *Bos taurus* (bt), *Mus musculus* (mm), *Gallus gallus* (gg), *Danio rerio* (dr), and *Xenopus laevis* (xl). The sequences are colored based on conservation with red being highly conserved and yellow being poorly conserved. The predicted secondary structure elements are shown below the sequence alignment. Multiple sequence alignment was performed with Clustal Omega (EMBL-EBI) and edited with Jalview 2.11.0 (Waterhouse et al, 2009). The secondary structure prediction was done using the web services in Jalview, using Jpred. The N-terminal tail, loop region and dimerization domain of Borealin are highlighted with boxes. (**B**) Sequence alignment of the first 190 amino acids of INCENP orthologs from *Homo sapiens* (hs), *Bos taurus* (bt), *Mus musculus* (mm), *Gallus gallus* (gg), *Danio rerio* (dr), and *Xenopus laevis* (xl). The sequences are colored based on conservation with red being highly conserved and yellow being poorly conserved. The predicted secondary structure elements are shown below the sequence alignment. Multiple sequence alignment was performed with Clustal Omega (EMBL-EBI) and edited with Jalview 2.11.0 (Waterhouse et al, 2009). The secondary structure prediction was done using the web services in Jalview, using Jpred. The basic IDR region of INCENP (including the "RRKKRR" motif and two additional positive stretches) and the PxVxI motif are highlighted with boxes in the alignment. (**C**) SEC chromatogram for $CPC_{I1\text{-}190SB}$-H3T3ph NCP complex formation and the corresponding SDS-PAGE gel for the run. The peak fractions were pooled and used to solve the cryo-EM structure of the complex. (**D**) Mass photometry analysis of the CPC-NCP complex. Population distribution of the CPC-NCP complex sample at 50 nM. Gaussian fitting identified three populations; 71 ± 13 kDa corresponding to CPC monomer (theoretically calculated 69.3 kDa), 122 ± 16 kDa corresponding to CPC dimer (theoretically calculated 138.6 kDa), and 340 ± 16.9 kDa which corresponds to the CPC-NCP complex with a stoichiometry of 2:1 (CPC:NCP; theoretically calculated 337.95 kDa). The event counts for each peak are also indicated below the mass estimation. (**E**) Representative cryo-EM micrograph with the scale bar corresponding to 120 nm. (**F**) Representative 2D-classes from the processing pipeline of the cryo-EM structure from cryoSPARC. Different orientations of NCP can be clearly identified from the classes. Scale bar equals 120 Å. Source data are available online for this figure.

© The Author(s)

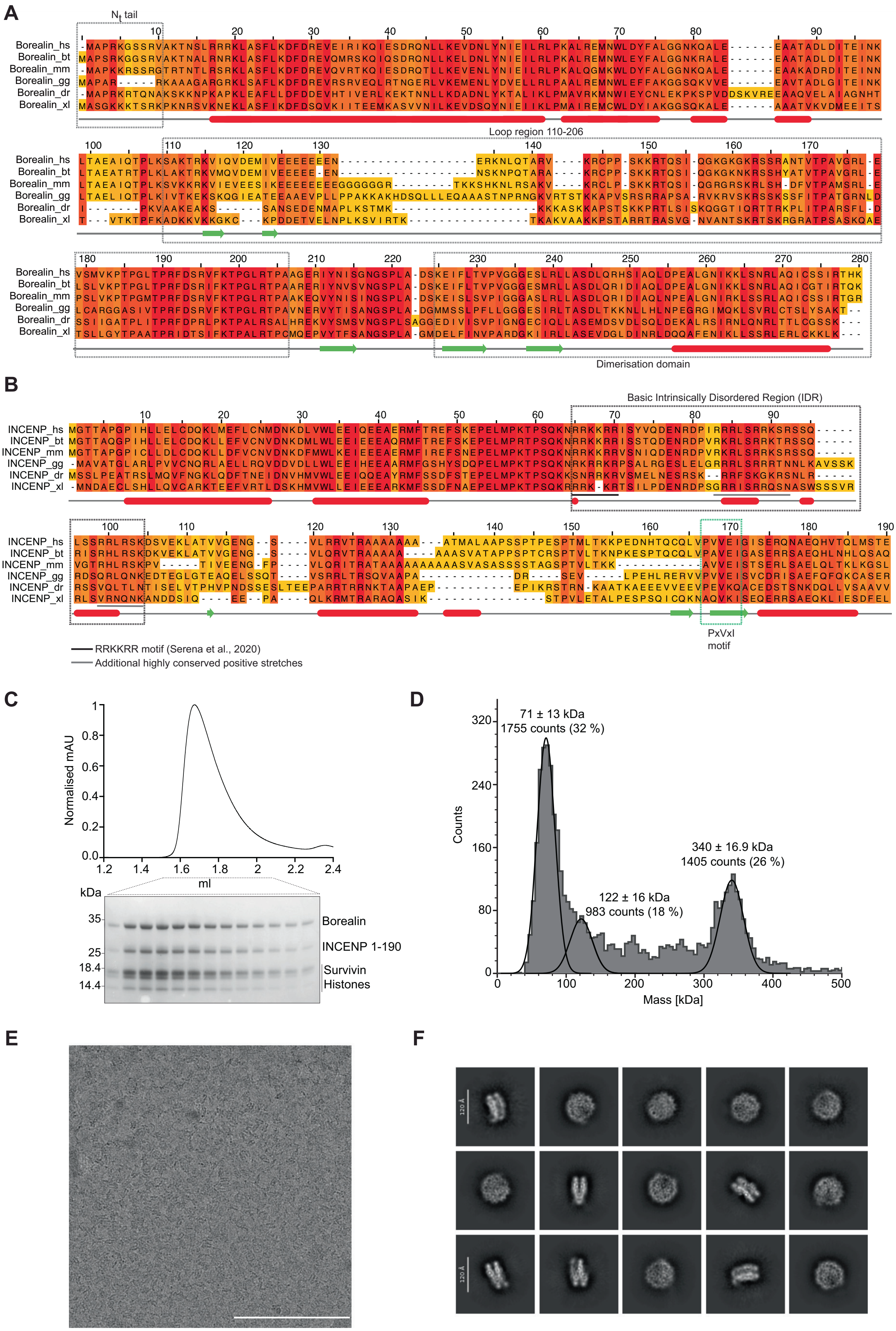
A
Nt tail
Borealin_hs
Borealin_bt
Borealin_mm
Borealin_gg
Borealin_dr
Borealin_xl
Loop region 110-206
Dimerisation domain
B
Basic Intrinsically Disordered Region (IDR)
INCENP_hs
INCENP_bt
INCENP_mm
INCENP_gg
INCENP_dr
INCENP_xl
PxVxI motif
RRKKRR motif (Serena et al., 2020)
Additional highly conserved positive stretches
C
Normalised mAU
ml
kDa
35
25
18.4
14.4
Borealin
INCENP 1-190
Survivin
Histones
D
71 ± 13 kDa
1755 counts (32 %)
122 ± 16 kDa
983 counts (18 %)
340 ± 16.9 kDa
1405 counts (26 %)
Counts
Mass [kDa]
E
F
120 Å

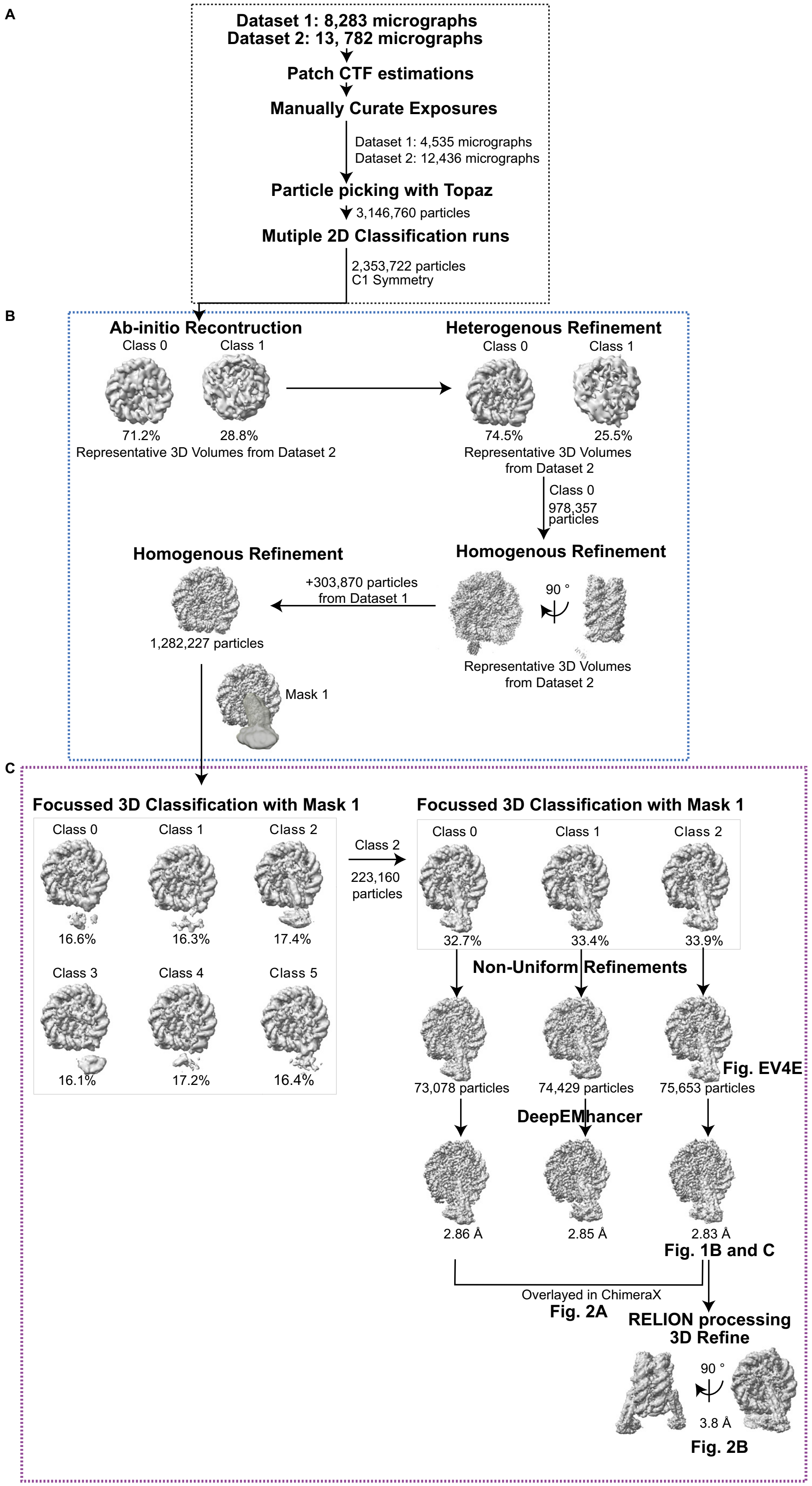
A
Dataset 1: 8,283 micrographs
Dataset 2: 13, 782 micrographs
Patch CTF estimations
Manually Curate Exposures
Dataset 1: 4,535 micrographs
Dataset 2: 12,436 micrographs
Particle picking with Topaz
3,146,760 particles
Mutiple 2D Classification runs
2,353,722 particles
C1 Symmetry
B
Ab-initio Recontruction
Class 0
Class 1
71.2%
28.8%
Representative 3D Volumes from Dataset 2
Heterogenous Refinement
Class 0
Class 1
74.5%
25.5%
Representative 3D Volumes from Dataset 2
Class 0
978,357 particles
Homogenous Refinement
90 °
Representative 3D Volumes from Dataset 2
+303,870 particles from Dataset 1
Homogenous Refinement
1,282,227 particles
Mask 1
C
Focussed 3D Classification with Mask 1
Class 0
Class 1
Class 2
16.6%
16.3%
17.4%
Class 3
Class 4
Class 5
16.1%
17.2%
16.4%
Class 2
223,160 particles
Focussed 3D Classification with Mask 1
Class 0
Class 1
Class 2
32.7%
33.4%
33.9%
Non-Uniform Refinements
Fig. EV4E
73,078 particles
74,429 particles
75,653 particles
DeepEMhancer
2.86 Å
2.85 Å
2.83 Å
Fig. 1B and C
Overlayed in ChimeraX
Fig. 2A
RELION processing
3D Refine
90 °
3.8 Å
Fig. 2B

 © The Author(s)

Figure EV2. The processing workflow of the $CPC_{I1-190SB}$-NCP cryo-EM dataset.

(**A**) Initial stages of processing involving micrograph import, CTF estimation, particle picking and 2D classifications. (**B**) Generation of 3D volumes using ab initio reconstruction, heterogeneous and homogeneous refinements. Particles with good nucleosome density were selected for downstream processing. (**C**) Focused 3D classification runs with a mask for CPC density were performed to select particles with well-defined CPC-NCP density. Further non-uniform refinement and DeepEMhancer runs improved the quality of the EM map (Sanchez-Garcia et al, 2021). Particles from Class 2 of the final focused 3D classification were exported to RELION 4.0, where 3D Refine with blush regularization was performed to obtain a map with double occupancy (Kimanius et al, 2024). The maps shown in the different main and expanded view (EV) figures are indicated clearly with corresponding figure numbers.

© The Author(s)

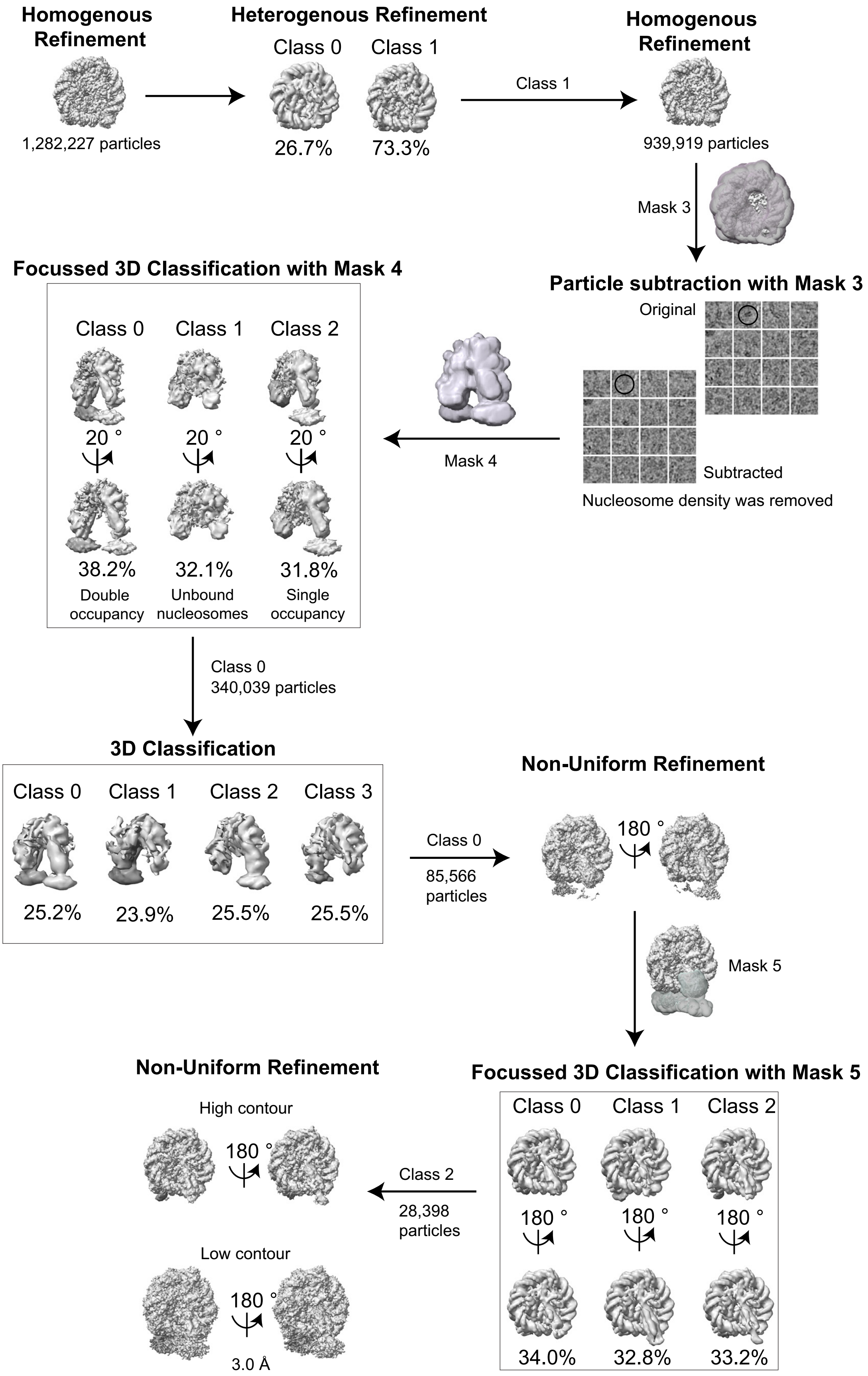
Homogenous Refinement
1,282,227 particles
Heterogenous Refinement
Class 0
Class 1
26.7%
73.3%
Class 1
Homogenous Refinement
939,919 particles
Mask 3
Particle subtraction with Mask 3
Original
Subtracted
Nucleosome density was removed
Mask 4
Focussed 3D Classification with Mask 4
Class 0
Class 1
Class 2
20 °
38.2%
32.1%
31.8%
Double occupancy
Unbound nucleosomes
Single occupancy
Class 0
340,039 particles
3D Classification
Class 0
Class 1
Class 2
Class 3
25.2%
23.9%
25.5%
25.5%
Class 0
85,566 particles
Non-Uniform Refinement
180 °
Mask 5
Focussed 3D Classification with Mask 5
Class 0
Class 1
Class 2
180 °
34.0%
32.8%
33.2%
Class 2
28,398 particles
Non-Uniform Refinement
High contour
Low contour
180 °
3.0 Å

Fig. EV4F

Figure EV3. The alternate processing workflow of the $CPC_{I1-190SB}$-NCP cryo-EM dataset to obtain a double occupancy map.

Particles from the homogeneous refinement job shown in Fig. EV2B were subjected to heterogeneous refinement and subsequently particle subtraction to remove the density for NCP. Focused 3D classification was performed to separate the doubly occupied NCPs from singly occupied and unbound NCPs. The selected class containing 85,566 particles (which makes up 6.67% of particles from the parent homogeneous refinement job) was further subjected to 3D classification runs and refinements to obtain the map depicted in Fig. EV4F.

© The Author(s)

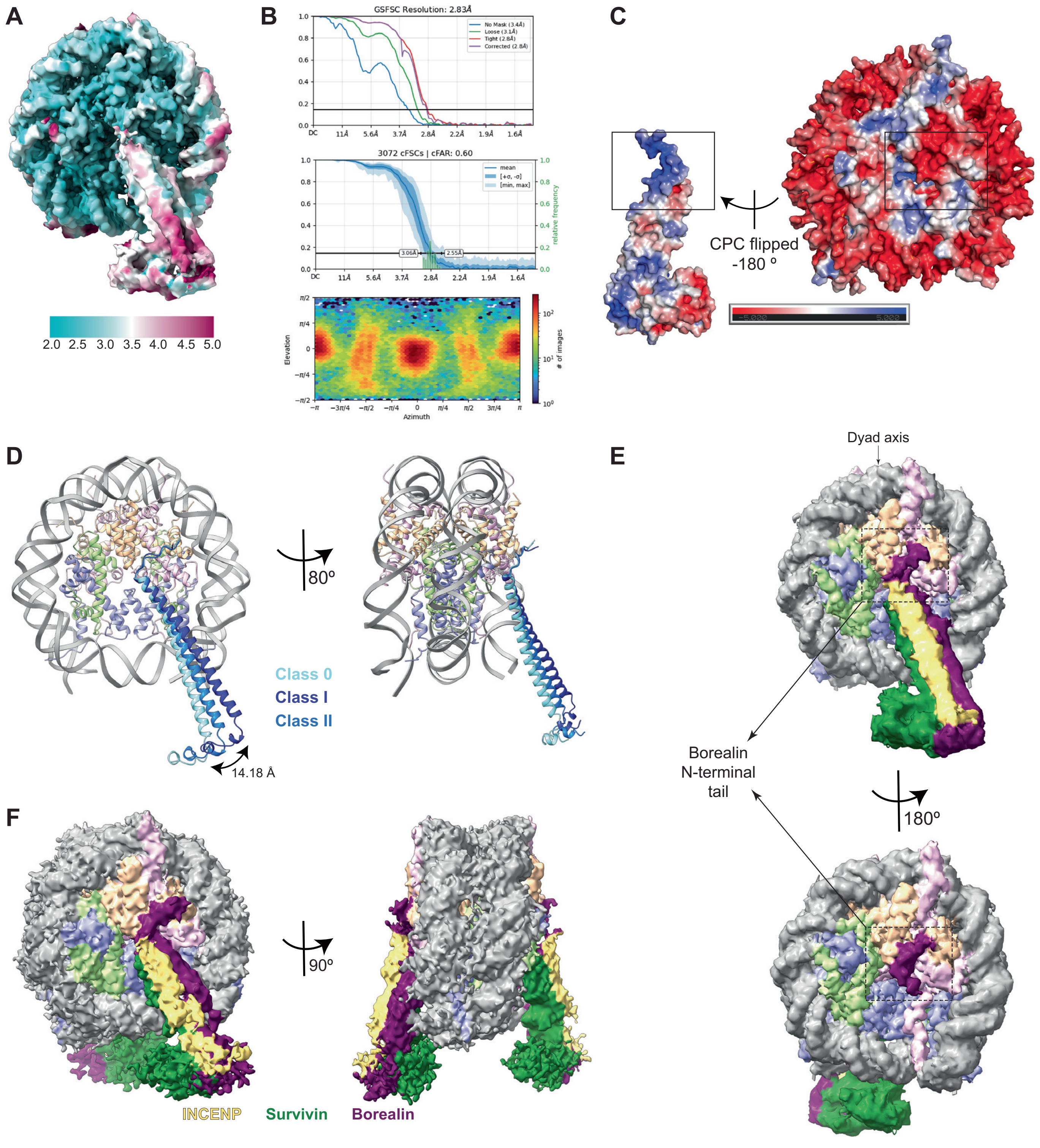
A
2.0 2.5 3.0 3.5 4.0 4.5 5.0
B
GSFSC Resolution: 2.83Å
No Mask (3.4Å)
Loose (3.1Å)
Tight (2.8Å)
Corrected (2.8Å)
3072 cFSCs | cFAR: 0.60
mean
relative frequency
3.06Å
2.55Å
Elevation
Azimuth
# of images
C
CPC flipped
-180 º
D
80º
Class 0
Class I
Class II
14.18 Å
E
Dyad axis
Borealin
N-terminal
tail
180º
F
90º
INCENP
Survivin
Borealin

 © The Author(s)

Figure EV4. Cryo-EM map and structure analysis of the $CPC_{I1\text{-}190SB}$-NCP complex.

(**A**) Local resolution map of the cryo-EM density (from Fig. 1B,C) is depicted in a cyan to pink color gradient. The scale bar represents the corresponding resolution in Å. (**B**) Corresponding GSFSC plot, cFSCs curve, and azimuth plot from cryoSPARC for the cryo-EM density. (**C**) Cryo-EM model surface colored by electrostatic potential (APBS using Pymol version 3.1), with CPC rotated −180 degrees around the *y*-axis to highlight the highly basic Borealin N-terminal tail (box, left), which binds to the acidic patch of the nucleosome (box, right). (**D**) Corresponding atomic model for the composite density map shown in Fig. 2A (only the Borealin from each class is shown for clarity). Histones are depicted in violet (Histone H3), light green (Histone H4), pink (Histone H2A), and orange (Histone H2B), and the DNA is shown in gray. Borealin from the three classes representing the separate positions of CPC are shown in different shades of blue. CPC has flexibility in both directions, horizontally and vertically, while being tethered to the nucleosome via the N-terminal tail of Borealin. The left image depicts the horizontal flexibility of the complex, while the right image shows the vertical motion of CPC. The displacement is 14.18 Å (measured: Cα to Cα distance of Borealin between Class 0 and Class 1) (**E**) Non-Uniform refinement map for Class 2 (parent to the DeepEMhancer map depicted in Fig. 1B,C) (top), and rotated 180 degrees across *y*-axis (bottom) to visualize the Borealin N-terminal tail occupancy on the opposite face of the nucleosome. (**F**) Cryo-EM density map obtained through the alternate processing pipeline detailed in Fig. EV3. CPC densities can be seen on both faces of the nucleosome.

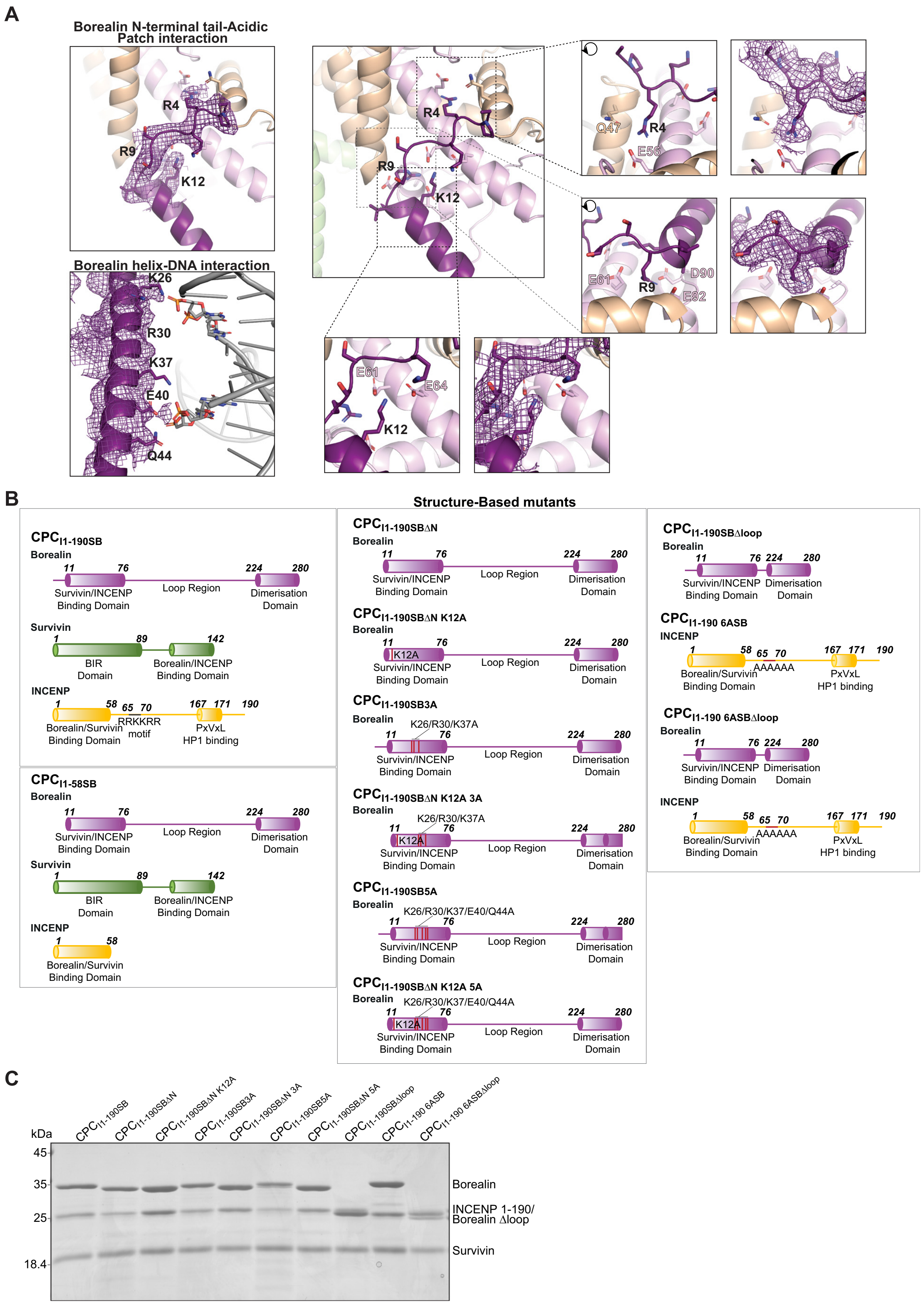
A
Borealin N-terminal tail-Acidic Patch interaction
R4
R9
K12
Borealin helix-DNA interaction
K26
R30
K37
E40
Q44
Q47
E56
E61
D90
E92
E64
B
Structure-Based mutants
CPC I1-190SB
Borealin
11 76 224 280
Survivin/INCENP Binding Domain
Loop Region
Dimerisation Domain
Survivin
1 89 142
BIR Domain
Borealin/INCENP Binding Domain
INCENP
1 58 65 70 167 171 190
Borealin/Survivin Binding Domain
RRKKRR motif
PxVxL HP1 binding
CPC I1-58SB
CPC I1-190SBΔN
CPC I1-190SBΔN K12A
K12A
CPC I1-190SB3A
K26/R30/K37A
CPC I1-190SBΔN K12A 3A
CPC I1-190SB5A
K26/R30/K37/E40/Q44A
CPC I1-190SBΔN K12A 5A
CPC I1-190SBΔloop
CPC I1-190 6ASB
AAAAAA
CPC I1-190 6ASBΔloop
C
kDa
45
35
25
18.4
CPC I1-190SB
CPC I1-190SBΔN
CPC I1-190SBΔN K12A
CPC I1-190SB3A
CPC I1-190SBΔN 3A
CPC I1-190SB5A
CPC I1-190SBΔN 5A
CPC I1-190SBΔloop
CPC I1-190 6ASB
CPC I1-190 6ASBΔloop
Borealin
INCENP 1-190/ Borealin Δloop
Survivin

 © The Author(s)

Figure EV5. Quality of the Cryo-EM map at key interaction interfaces and cartoons of $CPC_{I1\text{-}190SB}$ mutants highlighting the location of the mutations.

(**A**) The corresponding zoomed-in image of the N-terminal tail-acidic patch interaction (left, top), and the Borealin helix-DNA interaction (left, bottom) of Fig. 1B,C, respectively, with the EM density depicted as a mesh. The zoomed-in image of the N-terminal tail-acidic patch interaction involving the Borealin residues Arg 4, Arg 9, and Lys 12 is depicted in the center. The three interactions: Borealin Arg 4 interaction with Gln 47 of H2B and Glu 56 of H2A (right, top), Borealin Arg 9 interaction with Glu 61, Asp 90, and Glu 92 of H2A (right, bottom), and Borealin Lys 12 interaction with Glu 61 and Glu 64 of H2A (center, bottom). The panels adjacent to each depict the EM density as a mesh. (**B**) Schematic diagram depicting the domain architectures of $CPC_{I1\text{-}190SB}$ and $CPC_{I1\text{-}58SB}$. The domain architecture for the mutated proteins is depicted for the structure-based mutants ($CPC_{I1\text{-}190SB\Delta N}$, $CPC_{I1\text{-}190SB\Delta N\ K12A}$, $CPC_{I1\text{-}190SB3A}$, $CPC_{I1\text{-}190SB\Delta N\ K12A\ 3A}$, $CPC_{I1\text{-}190SB5A}$, and $CPC_{I1\text{-}190SB\Delta N\ K12A\ 5A}$) and displayed in the center panel. Additionally, the domain architecture for $CPC_{I1\text{-}190SB\Delta loop}$, $CPC_{I1\text{-}190\ 6ASB}$, and $CPC_{I1\text{-}190\ 6ASB\Delta loop}$ constructs is depicted on the right. Point mutations are depicted with red bars. (**C**) Representative SDS-PAGE gel with the $CPC_{I1\text{-}190SB}$, $CPC_{I1\text{-}190SB\Delta N}$, $CPC_{I1\text{-}190SB\Delta N\ K12A}$, $CPC_{I1\text{-}190SB3A}$, $CPC_{I1\text{-}190SB\Delta N\ K12A\ 3A}$, $CPC_{I1\text{-}190SB5A}$, and $CPC_{I1\text{-}190SB\Delta N\ K12A\ 5A}$, $CPC_{I1\text{-}190SB\Delta loop}$, $CPC_{I1\text{-}190\ 6ASB}$, and $CPC_{I1\text{-}190\ 6ASB\Delta loop}$ proteins used for SPR and EMSA experiments shown in Figs. 3, 4, and EV6. Source data are available online for this figure.

© The Author(s)

A

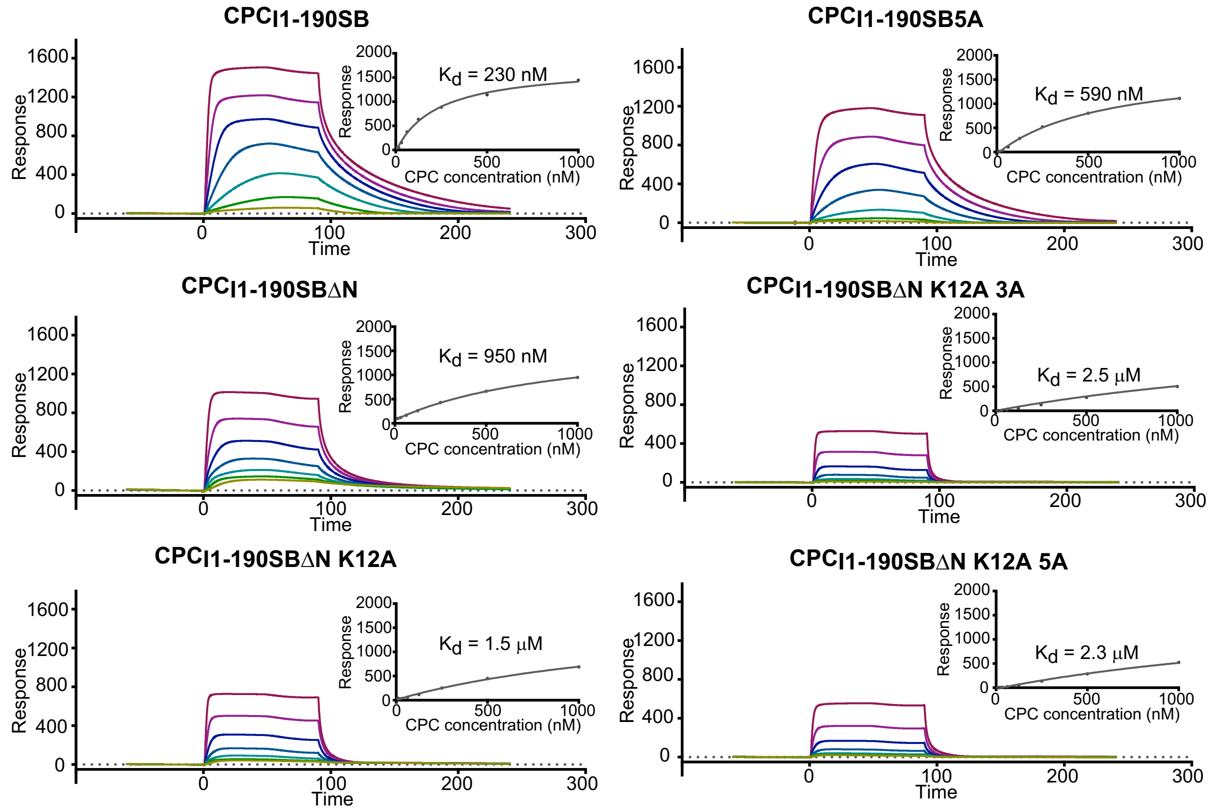

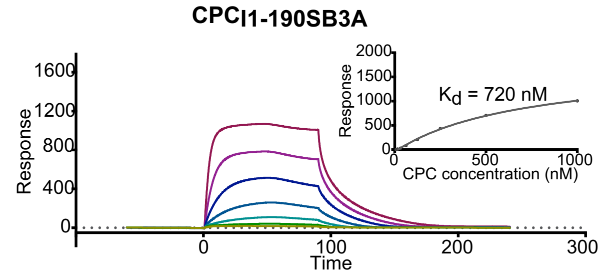

B

Sample	Kd ± SEM (nM)
$CPC_{I1\text{-}190SB}$	243.3 ± 6.7
$CPC_{I1\text{-}190SB\Delta N}$	1150 ± 200
$CPC_{I1\text{-}190SB\Delta N\ K12A}$	1350 ± 150
$CPC_{I1\text{-}190SB3A}$	725 ± 5
$CPC_{I1\text{-}190SB5A}$	590 ± 3
$CPC_{I1\text{-}190SB\Delta N\ K12A\ 3A}$	2767 ± 425.6
$CPC_{I1\text{-}190SB\Delta N\ K12A\ 5A}$	3050 ± 750

C

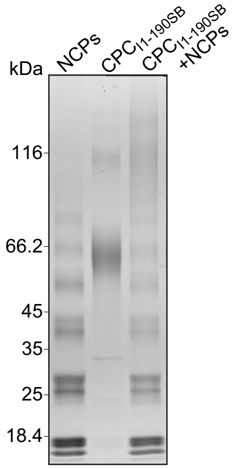

D

Protein	Peptide	-UV	+ UV	Modification	Position
Borealin	$_{61}$LPKALREMNWLDYFALGGNK$_{80}$		✓	d	w/o loc.
	$_{64}$ALREMNWLDYFALGGNK$_{80}$		✓	CC-H3N1-H2O1	D72
	$_{64}$ALREMNWLDYFALGGNK$_{80}$	✓	✓	AT-H3O4P1	N79
	$_{64}$ALREMNWLDYFALGGNKQALEEAATADLDITEINK$_{98}$		✓	dC-H3N1-H3O4P1	w/o loc.
	$_{81}$QALEEAATADLDITEINK$_{98}$		✓	C-H3O4P1	L83
	$_{81}$QALEEAATADLDITEINK$_{98}$		✓	dG-H3N1	A87
	$_{81}$QALEEAATADLDITEINK$_{98}$		✓	dG-H3N1	D90
	$_{99}$LTAEAIQTPLK$_{109}$		✓	CT-H2O1	A101
	$_{116}$VIQVDEMIVEEEEEEENER$_{134}$		✓	GT	w/o loc.
	$_{116}$VIQVDEMIVEEEEEEENERK$_{135}$		✓	GT	V119
	$_{116}$VIQVDEMIVEEEEEEENERK$_{135}$	✓	✓	T	V119

Protein	Peptide	-UV	+ UV	Modification	Position
INCENP	$_{1}$MGTTAPGPIHLLELCDQK$_{18}$		✓	GT-H3O4P1	H10
	$_{30}$DLVWLEEIQEEAER$_{43}$		✓	TC-H3N1-H1O3P1	w/o loc.
	$_{71}$ISYVQDENRDPIR$_{83}$		✓	CT	D76
	$_{71}$ISYVQDENRDPIR$_{83}$	✓	✓	CT	E77
	$_{155}$KPEDNHTQCQLVPVVEIGISER$_{176}$		✓	T-H1O3P1	V166
	$_{155}$KPEDNHTQCQLVPVVEIGISER$_{176}$		✓	T	w/o loc.
	$_{156}$PEDNHTQCQLVPVVEIGISER$_{176}$		✓	d-H3O4P1	w/o loc.
	$_{177}$QNAEQHVTQLMSTE$_{190}$		✓	AG	S188

© The Author(s)

Figure EV6. SPR and UV crosslinking/MS analysis of the $CPC_{I1\text{-}190SB}$-NCP complex.

(**A**) Representative SPR sensorgrams of the interaction between different CPC complexes ($CPC_{I1\text{-}190SB}$, $CPC_{I1\text{-}190SB\Delta N}$, $CPC_{I1\text{-}190SB\Delta N\ K12A}$, $CPC_{I1\text{-}190SB3A}$, $CPC_{I1\text{-}190SB\Delta N\ K12A\ 3A}$, $CPC_{I1\text{-}190SB5A}$, and $CPC_{I1\text{-}190SB\Delta N\ K12A\ 5A}$) and H3T3ph NPCs immobilized on the surface of a neutravidin sensor chip. Minimum of two biological replicates. (**B**) Mean values determined for the equilibrium K_d are shown in the table. (**C**) Representative SDS-PAGE of the EDC-crosslinked sample from Fig. 4A. (**D**) Sequences of the DNA-modified Borealin and INCENP peptides identified in the UV crosslinking experiments. Presence or absence in the non-UV crosslinked (control) and the UV crosslinked conditions are shown, together with the modification assigned and the position. Source data are available online for this figure.

© The Author(s)

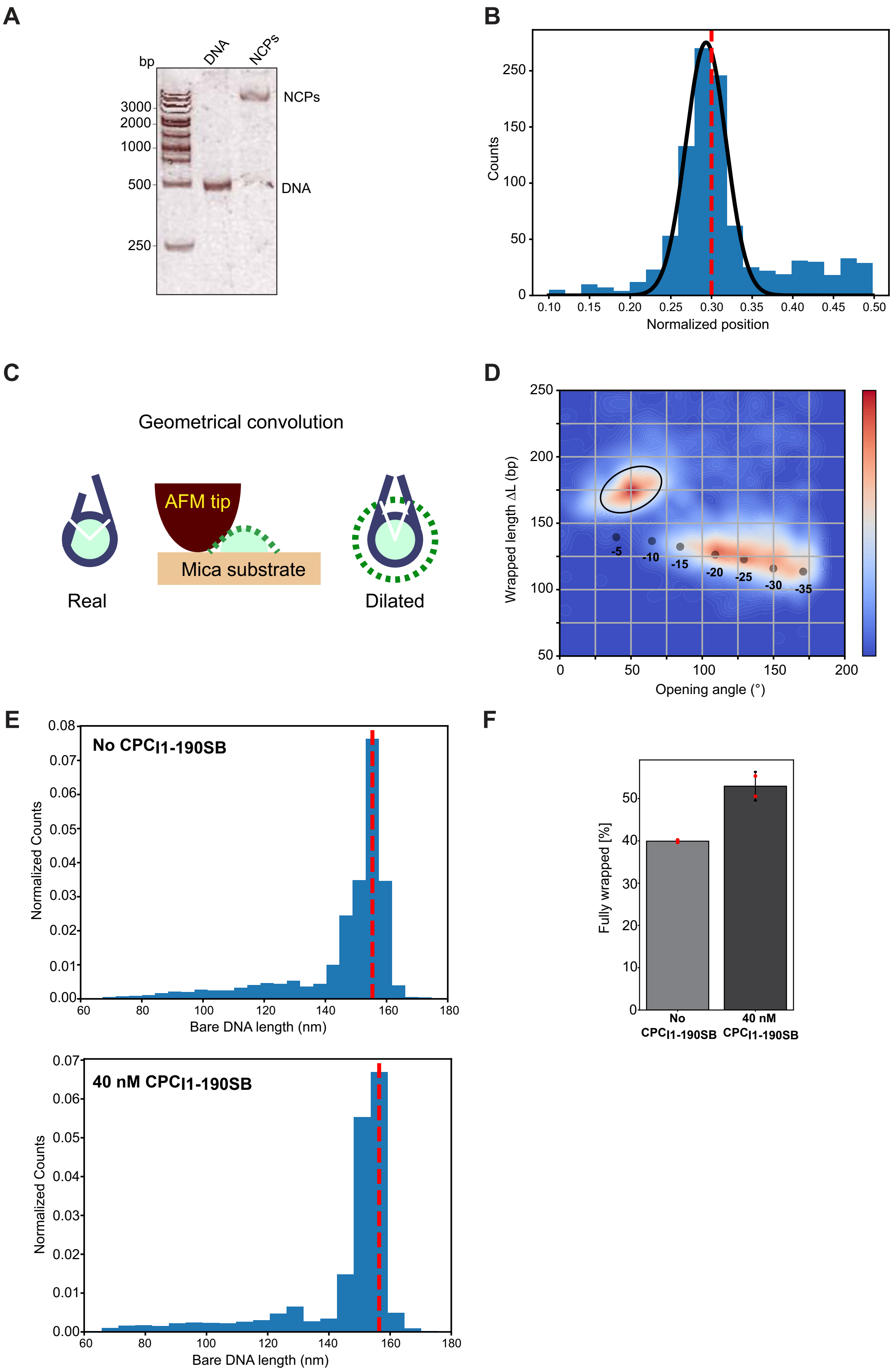
A
bp
DNA
NCPs
3000
2000
1000
500
250
NCPs
DNA
B
Counts
Normalized position
C
Geometrical convolution
AFM tip
Mica substrate
Real
Dilated
D
Wrapped length ΔL (bp)
Opening angle (°)
-5
-10
-15
-20
-25
-30
-35
E
No CPCI1-190SB
Normalized Counts
Bare DNA length (nm)
40 nM CPCI1-190SB
Normalized Counts
Bare DNA length (nm)
F
Fully wrapped [%]
No CPCI1-190SB
40 nM CPCI1-190SB

 © The Author(s)

Figure EV7. Characterization of $CPC_{I1\text{-}190SB}$-NCP complex by AFM.

(**A**) Native gel with DNA construct used for AFM, before and after reconstitution with H3T3ph octamers. (**B**) Distribution of normalized nucleosome positions, quantified as the ratio of the short arm length l_1 over the sum of the arm lengths $l_1 + l_2$. Only fully wrapped nucleosomes are taken into account because asymmetric nucleosome unwrapping shifts the distribution. The red dashed line indicates the nucleosome position as expected from the DNA construct design. The full black line is a Gaussian fit to the data with a mean value of 0.29. (**C**) Schematic depiction indicating that geometrical convolution by the AFM tip distorts the real structure and leads to an underestimation of the nucleosome opening angle. (**D**) Two-dimensional kernel density estimate with indicated positions of different unwrapping states (numbers of bp unwrapped with respect to the fully wrapped state), as deduced from AFM image simulations (Konrad et al, 2022). (**E**) AFM characterization of bare DNA constructs in H3T3ph nucleosomes nucleosome samples in the absence (top) and presence (bottom) of CPC and reconstituted DNA contour length distribution as measured via automated readout. The mode of the distribution (red dashed line) is at contour length 155.4 nm in the absence and 156.4 nm in the presence of CPC, corresponding to 0.32 nm/bp, in good agreement with previous AFM measurements of DNA length (Rivetti et al, 1996; Konrad et al, 2021b). The experimental rise per base pair is used to convert the units of wrapped length ΔL from nm to bp. (**F**) The fraction of fully wrapped nucleosomes is significantly different in the absence and presence of 40 nM $CPC_{I1\text{-}190SB}$. Red datapoints represents calculated fractions for each biological repeat, indicating good agreement and reproducibility. Source data are available online for this figure.

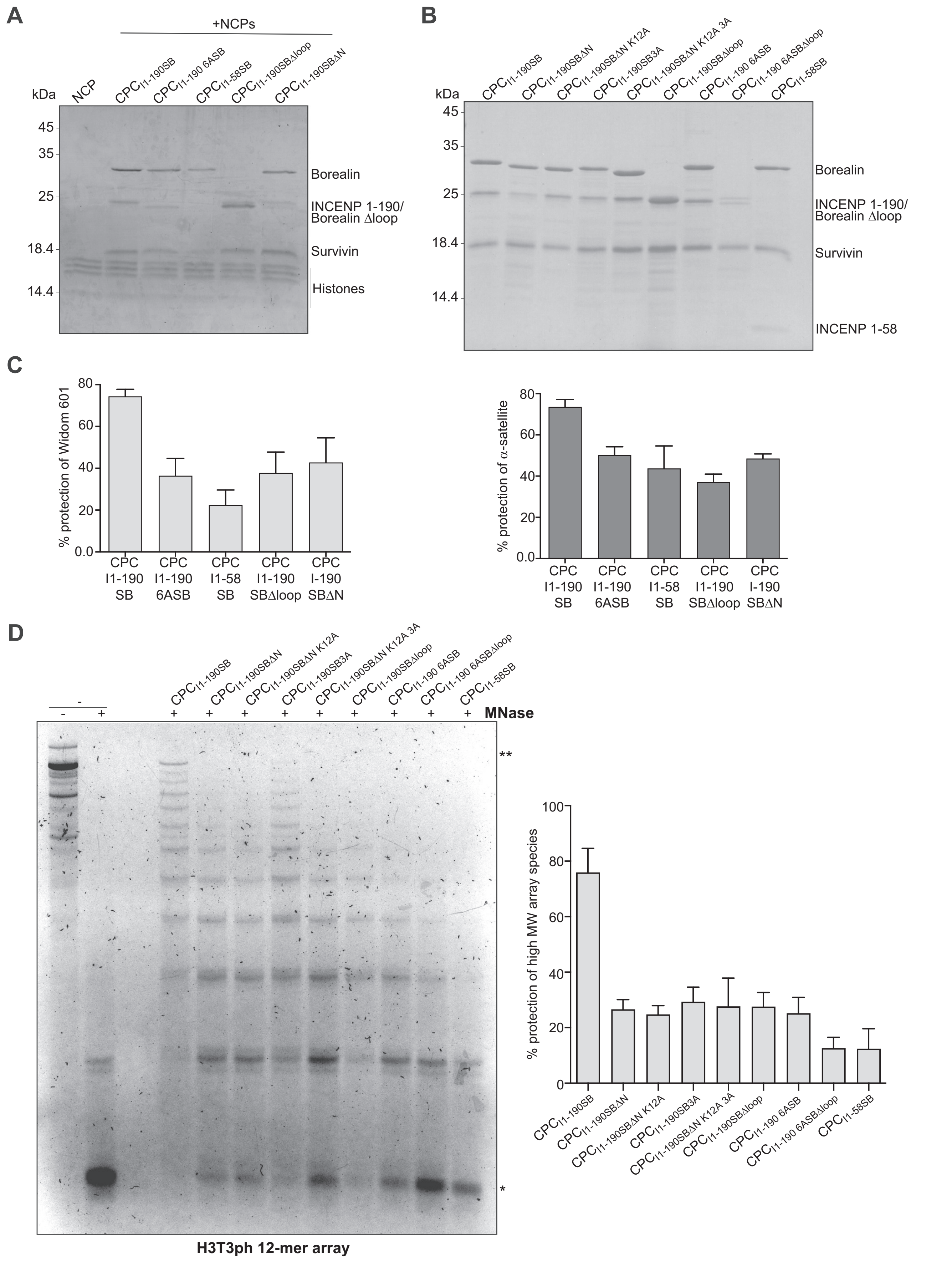
A
+NCPs
kDa
NCP
CPCI1-190SB
CPCI1-190 6ASB
CPCI1-58SB
CPCI1-190SBΔloop
CPCI1-190SBΔN
45
35
25
18.4
14.4
Borealin
INCENP 1-190/ Borealin Δloop
Survivin
Histones
B
kDa
CPCI1-190SB
CPCI1-190SBΔN
CPCI1-190SBΔN K12A
CPCI1-190SB3A
CPCI1-190SBΔN K12A 3A
CPCI1-190SBΔloop
CPCI1-190 6ASB
CPCI1-190 6ASBΔloop
CPCI1-58SB
45
35
25
18.4
14.4
Borealin
INCENP 1-190/ Borealin Δloop
Survivin
INCENP 1-58
C
% protection of Widom 601
80
60
40
20
0.0
CPC I1-190 SB
CPC I1-190 6ASB
CPC I1-58 SB
CPC I1-190 SBΔloop
CPC I-190 SBΔN
% protection of α-satellite
80
60
40
20
0.0
CPC I1-190 SB
CPC I1-190 6ASB
CPC I1-58 SB
CPC I1-190 SBΔloop
CPC I-190 SBΔN
D
CPCI1-190SB
CPCI1-190SBΔN
CPCI1-190SBΔN K12A
CPCI1-190SB3A
CPCI1-190SBΔN K12A 3A
CPCI1-190SBΔloop
CPCI1-190 6ASB
CPCI1-190 6ASBΔloop
CPCI1-58SB
-
- + + + + + + + + + +
MNase
**
*
H3T3ph 12-mer array
% protection of high MW array species
100
80
60
40
20
0
CPCI1-190SB
CPCI1-190SBΔN
CPCI1-190SBΔN K12A
CPCI1-190SB3A
CPCI1-190SBΔN K12A 3A
CPCI1-190SBΔloop
CPCI1-190 6ASB
CPCI1-190 6ASBΔloop
CPCI1-58SB

 © The Author(s)

Figure EV8. MNase protection assays with $CPC_{I1\text{-}190SB}$ mutants.

(**A**) SDS-PAGE gel with the inputs for the mono-nucleosome MNase assay shown in Fig. 5E. (**B**) SDS-PAGE gel with the CPC inputs of the MNase assay with 12-mer nucleosomal arrays shown in Fig. 5F. (**C**) Quantification of the mono-nucleosome MNase assay for Widom 601 NCP (left), and α-satellite NCP (right) from Fig. 5E ($n = 3$, mean ± SEM; data representative of three biological replicates). (**D**) Agarose gel for the MNase assay depicted in Fig. 5F. 12-mer array DNA is highlighted with double asterisks, and monomer DNA is highlighted with a single asterisk. Percentage of protection of higher MW array (4-mer to 12-mer) against MNase degradation quantified in the bar graph on the right ($n = 3$; mean ± SEM; data representative of three biological replicates). Source data are available online for this figure.

© The Author(s)

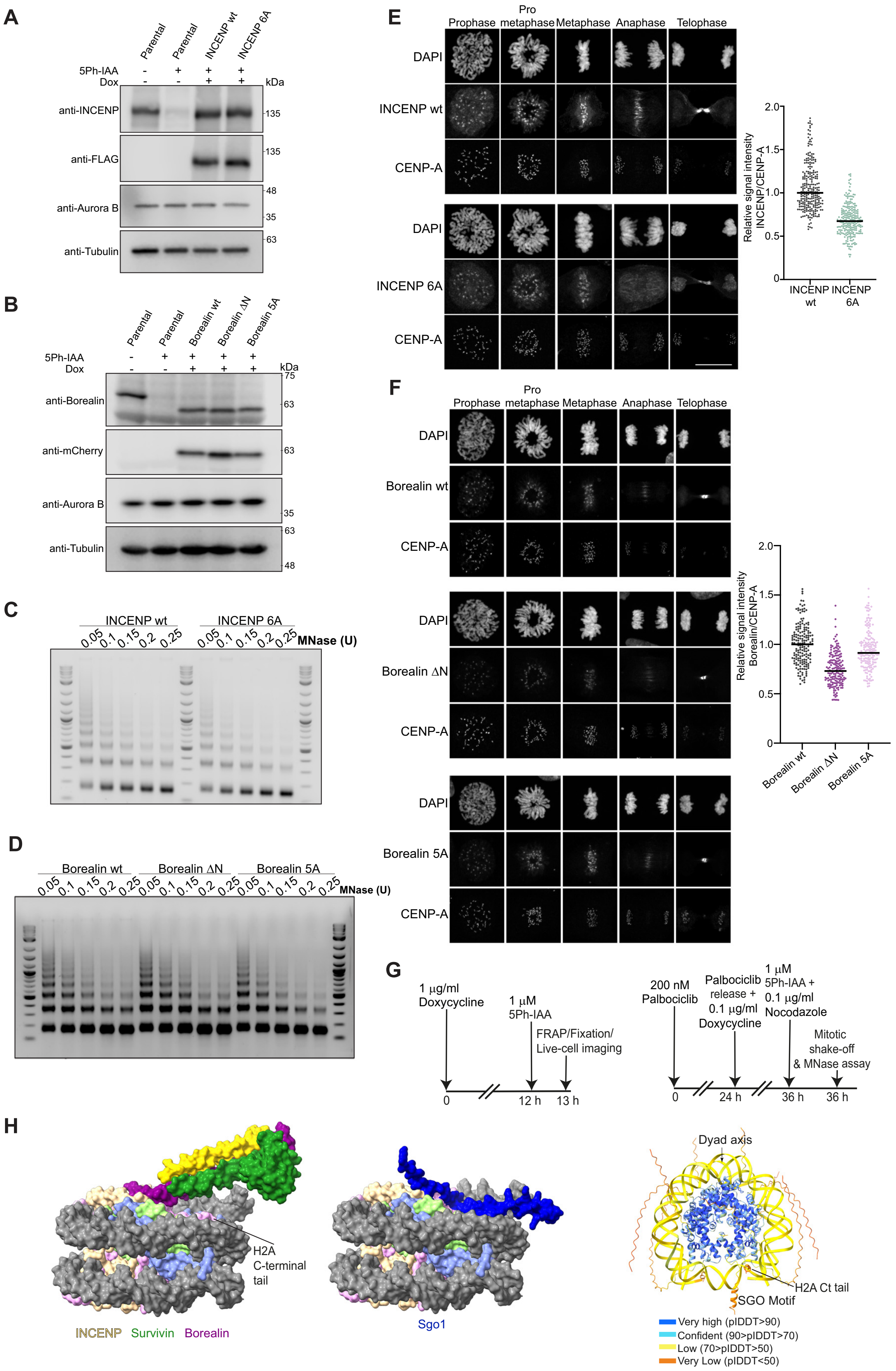
A
Parental
Parental
INCENP wt
INCENP 6A
5Ph-IAA
Dox
kDa
anti-INCENP
anti-FLAG
anti-Aurora B
anti-Tubulin
135
135
48
35
63
B
Parental
Parental
Borealin wt
Borealin ΔN
Borealin 5A
5Ph-IAA
Dox
kDa
anti-Borealin
anti-mCherry
anti-Aurora B
anti-Tubulin
75
63
63
35
63
48
C
INCENP wt
INCENP 6A
0.05 0.1 0.15 0.2 0.25
MNase (U)
D
Borealin wt
Borealin ΔN
Borealin 5A
0.05 0.1 0.15 0.2 0.25
MNase (U)
E
Prophase
Pro metaphase
Metaphase
Anaphase
Telophase
DAPI
INCENP wt
CENP-A
INCENP 6A
Relative signal intensity INCENP/CENP-A
INCENP wt
INCENP 6A
F
Borealin wt
Borealin ΔN
Borealin 5A
Relative signal intensity Borealin/CENP-A
G
1 μg/ml Doxycycline
1 μM 5Ph-IAA
FRAP/Fixation/ Live-cell imaging
0
12 h
13 h
200 nM Palbociclib
Palbociclib release + 0.1 μg/ml Doxycycline
1 μM 5Ph-IAA + 0.1 μg/ml Nocodazole
Mitotic shake-off & MNase assay
24 h
36 h
H
H2A C-terminal tail
INCENP Survivin Borealin
Sgo1
Dyad axis
H2A Ct tail
SGO Motif
Very high (pIDDT>90)
Confident (90>pIDDT>70)
Low (70>pIDDT>50)
Very Low (pIDDT<50)

 © The Author(s)

Figure EV9. Implications of CPC-NCP interaction on the centromeric levels of CPC and Sgo1 binding.

(**A**) Representative immunoblot for RPE1 cell lines expressing either INCENP wt or INCENP 6A mutant showing the expression levels of the different INCENP constructs used in Fig. 6. (**B**) Representative immunoblot for RPE1 cell lines expressing either Borealin wt, Borealin ΔN, and Borealin 5A showing the expression levels of the different INCENP constructs used in Fig. 6. (**C**) Ethidium bromide-stained agarose gel image corresponding to the MNase assay presented in Fig. 6A. (**D**) Ethidium bromide-stained agarose gel image corresponding to the MNase assay presented in Fig. 6B. (**E**) (Left panel) Representative fluorescence images for INCENP wt and INCENP 6A centromere localization at different stages of mitosis. DAPI was used for DNA staining and CENP-A for centromere staining. Scale bar, 10 µm. (Right panel) Scatter plot for the quantification of INCENP wt and INCENP 6A centromere localization during prometaphase. Relative fluorescence intensities of INCENP wt (black) and INCENP 6A (green), normalized to CENP-A, were measured in at least ten cells from two biological replicates. Individual data points are plotted. Horizontal black lines indicate median values. (**F**) (Left panel) Representative fluorescence images for Borealin wt, Borealin ΔN, and Borealin 5A centromere localization at different stages of mitosis. DAPI was used for DNA staining and CENP-A for centromere staining. Scale bar, 10 µm. (Right panel) Scatter plot for the quantification of Borealin wt, Borealin ΔN, and Borealin 5A centromere localization during prometaphase. Relative fluorescence intensities of Borealin wt (black), Borealin ΔN (purple), and Borealin 5A (pink), normalized to CENP-A, were measured in at least ten cells from two biological replicates. Individual data points are plotted. Horizontal black lines indicate median values. (**G**) (left panel) Diagram with the experimental timeline followed for the RPE1 experiments shown in Fig. 6C–E. (Right panel) Diagram with the experimental timeline followed for the RPE1 experiment shown in Fig. 6A,B. (**H**) Atomic model for the CPC-NCP complex (Class 0) with the H2A C-terminal tail indicated (left). AlphaFold 3 prediction of SGO motif (amino acids 466–527) of Sgo1 binding to H2AT120ph NCP (Abramson et al, 2024). The prediction colored by plDDT values is depicted on the right. The SGO motif-H2A tail interaction is predicted with low confidence ($70 > \text{plDDT} > 50$). The predicted model agrees with previously available biochemistry on the interfaces involved in SGO motif-NCP binding (Kawashima et al, 2010; Liu et al, 2013, 2015). CPC binding depicted on the left, occludes H2A C-terminal tail, likely making it inaccessible for Sgo1 binding. Source data are available online for this figure.

© The Author(s)

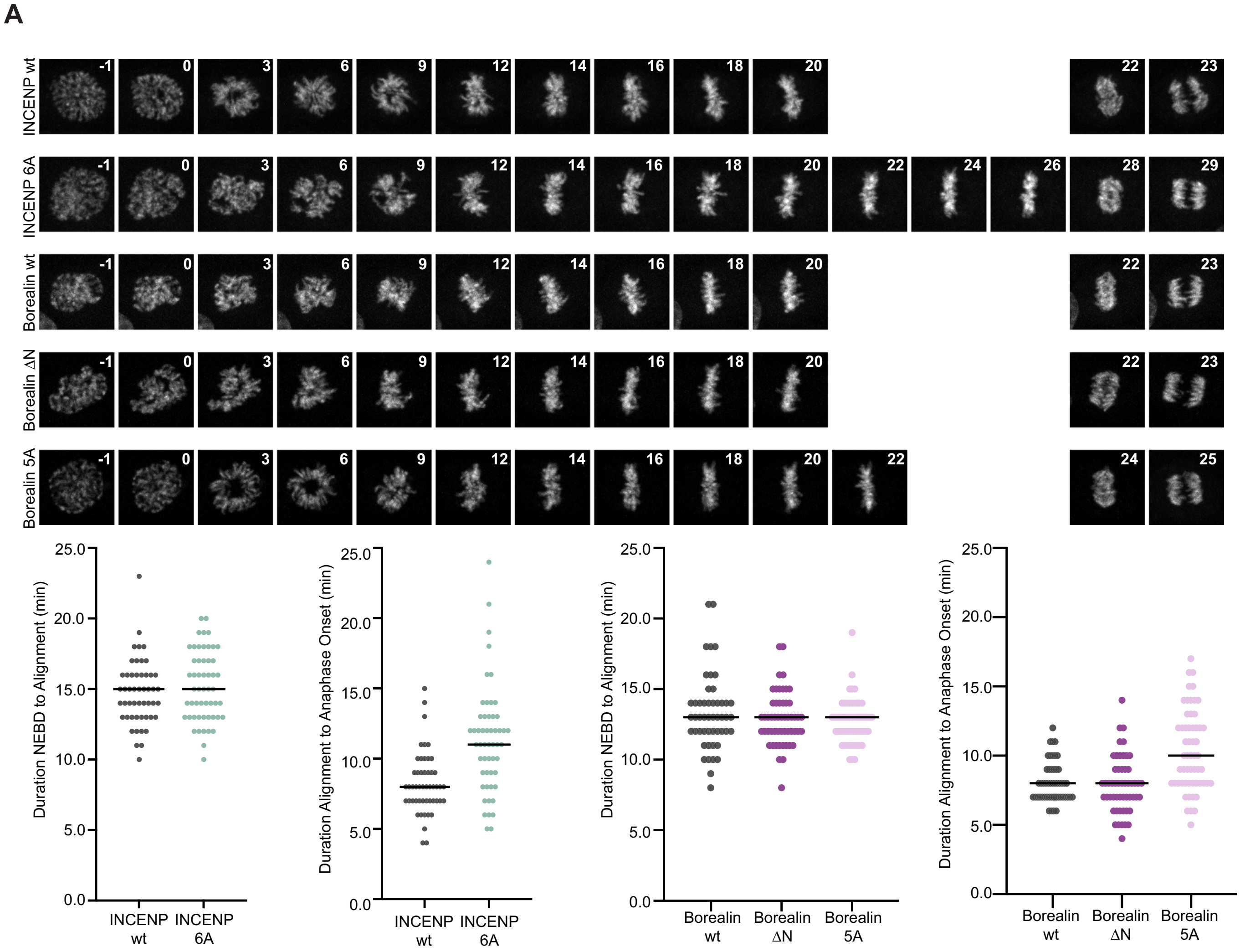

Figure EV10. Impact of perturbing CPC-NCP interaction on the timing of anaphase onset.

(**A**) Representative images from live-cell imaging experiments assessing mitotic timing in at least 50 cells from two biological replicates for INCENP wt (black), INCENP 6A (green), Borealin wt (black), Borealin ΔN (purple), and Borealin 5A mutant (pink). Scatter plots show the elapsed time (in minutes) from nuclear envelope breakdown (NEBD) to metaphase alignment and from metaphase alignment to anaphase onset for individual cells. Individual data points are plotted. Horizontal black lines indicate median values. Source data are available online for this figure.

 © The Author(s)
